# Changes in Speech Intelligibility, Health-Related Quality of Life, Depressive Symptoms, Anxiety, Perceived Stress, and Tinnitus-Induced Distress, in a Cohort of 227 Adults One Year After Cochlear Implantation: A Decade of Experience from a Single Tertiary Center

**DOI:** 10.3390/jcm14228143

**Published:** 2025-11-17

**Authors:** Heidi Olze, Moritz Gröschel, Agnieszka J. Szczepek

**Affiliations:** 1 Department of Otorhinolaryngology, Head and Neck Surgery, Charité—Universitätsmedizin Berlin, Corporate Member of Freie Universität Berlin and Humboldt Universität zu Berlin, 10117 Berlin, Germany; 2 Faculty of Medicine and Health Sciences, University of Zielona Góra, 65-046 Zielona Gora, Poland

**Keywords:** sensorineural hearing loss, cochlear implantation, tinnitus, anxiety, stress, depression, speech intelligibility

## Abstract

**Background/Objectives:** The purpose of this study was to analyze changes in speech intelligibility, health-related quality of life, and the degree of comorbidities (depressive and anxiety symptoms and tinnitus-related distress) in a large cohort of 227 adults who underwent auditory rehabilitation with a cochlear implant (CI). The second goal was to identify the factors that influence the health-related quality of life in this cohort. **Methods:** Pre- and one-year post-CI data were collected on speech intelligibility (Freiburg Monosyllabic Test, FS), subjective hearing ability (Oldenburg Inventory, OI), health-related quality of life (Nijmegen Cochlear Implant Questionnaire, NCIQ), depressive symptoms (General Depression Scale, ADS-L), anxiety (Generalized Anxiety Disorder 7, GAD-7), perceived stress (Perceived Stress Questionnaire, PSQ), and tinnitus-related distress (Tinnitus Questionnaire, TQ). **Results:** The Wilcoxon matched-pairs signed-rank test showed significant improvements across the entire cohort in speech intelligibility, subjective hearing, and the quality of life. The scores indicating anxiety, depressiveness, perceived stress, and tinnitus-related distress decreased. The Spearman correlation showed that before implantation, quality of life positively related to subjective hearing, while depression, anxiety, stress, and tinnitus distress were negatively correlated. After a year, these links persisted but grew stronger. Regression analyses found subjective hearing (OI) as a positive predictor, and depression (ADS-L) and tinnitus distress (TQ) as negative predictors of life quality, especially in patients with low or median NCIQ scores. **Conclusions:** In a substantial cohort of adult patients with diverse CI indications, auditory rehabilitation enhances speech intelligibility and subjective hearing, improves health-related quality of life, and reduces the severity of depressive and anxiety symptoms, as well as tinnitus-related distress. Subjective hearing contributes positively to quality of life, whereas depressive symptoms and tinnitus distress negatively impact quality of life in patients with low NCIQ scores post-CI, highlighting the importance of monitoring and psychological intervention.

## 1. Introduction

Hearing loss is the most common communication disorder worldwide and is expected to affect 2.5 billion people by 2050 [1]. Of those, 700 million should be undergoing auditory rehabilitation. One of the means of such rehabilitation is the surgical implantation of a cochlear implant (CI), which is now considered the standard treatment for severe or profound hearing loss caused by sensorineural hearing impairment. Currently, Germany and many other countries approve CI for pre- and post-lingual bilateral, unilateral, or asymmetric sensorineural hearing loss in both children and adults [2]. The main aim of CI is to support auditory rehabilitation [3], with success measured through audiological tests and patient questionnaires that assess speech understanding in various environments [2]. Beyond traditional audiometric measures, evaluating hearing-related quality of life offers valuable insight into the patient’s perspective during rehabilitation. A widely used tool is the Nijmegen Cochlear Implant Questionnaire (NCIQ), developed by Hinderink and colleagues [4]. Since its creation, the NCIQ has become a gold standard internationally for assessing changes in physical, psychological, and social aspects of life in hearing-impaired individuals before and after CI [5,6,7,8].

Several clinical studies evaluated the changes in quality of life related to hearing loss after auditory rehabilitation with CI. For instance, Cuda et al. found a significant improvement in the quality of life for CI recipients over 60 [9], while Baungaard et al. indicated that for patients aged 37 to 83, the effect lasts over two years [10]. A recent systematic review and meta-analysis examined data from 27 articles involving 1285 CI patients and found that implantation leads to an improved quality of life, as shown by an increase in the NCIQ score of more than 12 points [11]. This result reinforces the rationale for monitoring the subjective quality of life of implanted patients and for clinicians to respond to those who experience no or low improvement in this area.

Subjective speech intelligibility (SI) tests evaluate a patient’s personal perception of their hearing abilities in various everyday situations and are a valued tool during the process of auditory rehabilitation [2]. In English-speaking countries, the Speech, Spatial, and Qualities of Hearing Scale (SSQ) is often used as an SI test [12]. In Germany, a widely used SI test is the self-reported Oldenburg Inventory (OI), which assesses hearing performance in quiet conditions, background noise, and directional hearing [13]. One study investigating the correlation between the SSQ and NCIQ tests found the correlation to be statistically significant, although it did not report the correlation coefficient [12]. In contrast, another study confirmed these results and reported the correlations to be weak to moderate [14]. The OI was previously shown to positively correlate with NCIQ (moderate correlation) [15], but its predictive value for NCIQ was not yet assessed.

It has long been recognized that hearing loss in patients is linked to other conditions, such as tinnitus, depressive symptoms, or anxiety and stress. A global study in the USA estimated that in 2019, 28.1% of people in the USA had hearing impairment, with the prevalence increasing with age [16]. Of those with hearing loss, 32.5% reported having tinnitus. In line with this, a multicenter study by Chen et al. reported a 57.1% prevalence of tinnitus in older patients (mean age, 69.9; SD, 7.9) with confirmed hearing loss [17]. Furthermore, the severity of hearing loss is positively correlated with the level of tinnitus-related distress [18]. Among patients undergoing cochlear implantation, the prevalence of tinnitus was estimated to be 86% [19]. Similar to tinnitus, depressive symptoms are common among hearing-impaired adults, with a prevalence of 17% [20]. A South Korean population-based follow-up study of a quarter of a million individuals showed that persons with hearing impairment had a significantly higher risk of depression than the age-matched control group without hearing loss [21]. Interestingly, people under 65 were at a higher risk of developing depression than those over 65. Additionally, an Italian study of 1332 adults over 65 showed that greater severity of hearing loss is linked to more severe depressive symptoms [22], which was confirmed by a recent meta-analysis [23]. Also, anxiety often occurs alongside hearing impairment [24], and a meta-analysis showed the prevalence of anxiety disorders among people with sensorineural hearing loss to be 40% [20]. Furthermore, emotional stress has also been associated with hearing loss, and an elevated serum concentration of the stress hormone ACTH has been found to correlate with hearing thresholds in patients with sudden idiopathic sensorineural hearing loss [25].

Because the above comorbidities often occur among CI candidates, tests assessing their severity are frequently included in self-report test batteries used to monitor patients undergoing cochlear implantation. Our group has developed a “Berlin test battery” that includes NCIQ, OI, ADS-L, GAD-7, PSQ, and TQ to screen patients before and after implantation for the presence and severity of all of these comorbidities. Using that test battery, we have determined changes occurring in the variables following rehabilitation with CI, findings which were confirmed by others [11,26]. Additionally, significant correlations were found between the NCIQ and scores on tests for these conditions [26]. However, little is known about the factors that influence (either positively or negatively) the health-related quality of life of implanted patients. Such knowledge could enhance the process of auditory rehabilitation and improve the overall well-being of patients.

Over the past years, we have reported the outcomes of auditory rehabilitation with CI from various prospective studies, in which the CI candidates were grouped based on the type of hearing loss [26,27,28,29,30] or age [27,31]. In these reports, some or all of the variables of current interest—such as health-related quality of life, speech intelligibility, self-reported speech intelligibility, tinnitus-related distress, depressive symptoms, anxiety, and perceived stress—were used as primary or secondary outcome measures alongside audiological measurements. Here, we analyzed the entire cohort of adult CI patients, regardless of their age or type of hearing loss. The primary goal of our study was to evaluate the changes in speech intelligibility, health-related quality of life, tinnitus-related distress, depressive symptoms, anxiety, and perceived stress one year after cochlear implantation. Additionally, we hypothesized that certain variables might have influenced the NCIQ, predicting its outcome. Therefore, the second goal was to identify which (if any) of these variables affect the health-related quality of life.

## 2. Materials and Methods

The data were prospectively collected between 2013 and 2022 in a tertiary healthcare setting. The permit to conduct this study was issued by a local Ethics Committee (permit EA2/030/13).

The study design employed a cohort study, utilizing a retrospective analysis of prospectively collected data. The investigations were conducted in accordance with the principles outlined in the Declaration of Helsinki, and all participants provided written informed consent prior to participation. Out of 1060 patients admitted for cochlear implantation during that period, 227 patients (Table 1) were included in this study.

The inclusion criteria were:

- age over 18;
- willingness to participate;
- postlingually acquired severe or profound sensorineural hearing loss;
- compliance with questionnaire completion;
- written consent.

The exclusion criteria were:

- mental or cognitive impairment;
- inability to sign consent.

Patients with various types of hearing loss were included (asymmetric hearing loss, AHL—58 patients, single-sided (unilateral) deafness, SSD—43 patients, and DSD double-sided (bilateral) deafness—119 patients), and 7 patients with an undetermined type of hearing loss. The sample consisted of 99 men (43.6%) and 128 women (56.4%). Only data from patients for whom a full set of data was available before CI were used.

Cochlear implants and speech processors from three manufacturers were used: Cochlear (Cochlear Deutschland GmbH & Co. KG, Hannover, Germany): implants: CI24RE, CI512, CI612; speech processors: Nucleus 6, Nucleus 7, Kanso, Kanso 2; Med-El (Med-El Elektromedizinische Geräte Deutschland GmbH, Starnberg, Germany): implants: Concerto, Synchrony, Synchrony 2; speech processors: Opus 2, Sonnet, Sonnet 2, Rondo, Rondo 2, Rondo 3; and Advanced Bionics (Advanced Bionics GmbH, Hannover, Germany): implants: HiRes 90K, HiRes Ultra, HiRes Ultra 3D; speech processors: Naída CI Q70, Naída CI Q90, Naída CI M90. The latest models of implants and speech processors were always used.

All patients participated in outpatient cochlear implant rehabilitation at either our clinic or affiliated rehabilitation centers. The rehabilitation program spanned approximately 1.5 years and consisted of 20 sessions, focusing on auditory rehabilitation and speech processor fitting, followed by annual follow-up visits for processor adjustments. Thus, consistent auditory rehabilitation was maintained throughout the entire study period.

The data was gathered using standardized forms. The anonymized and coded test results were stored in a database on the central platform of Charité Universitätsmedizin Berlin. The data were validated and audited regularly.

### 2.1. Freiburg Monosyllabic Test (FS)

FS is a standard German speech audiometry test [32], during which lists of monosyllabic words are presented to the patients. The FS score indicates the percentage of words correctly repeated on a scale of 0–100%.

### 2.2. Oldenburg Inventory (OI): For Subjective Assessment of Hearing Ability

OI is a self-assessment questionnaire that measures the impact of hearing loss and rehabilitation on patients’ daily lives [13]. It assesses three areas: understanding speech in quiet environments (OI quiet), understanding speech in noisy environments (OI noise), and identifying the direction of a sound source (OI directional hearing). A higher score indicates better subjective hearing ability.

### 2.3. Nijmegen Cochlear Implant Questionnaire (NCIQ)

NCIQ consists of three domains: physical, psychological, and social functioning. The physical domain includes basic sound perception (NCIQ1), advanced sound perception (NCIQ2), and speech production (NCIQ3). The psychological domain includes self-esteem (NCIQ4), whereas the social domain includes limitations in social activities (NCIQ5) and social interactions (NCIQ6). A higher total or sub-domain score indicates better health-related quality of life related to the use of CI.

### 2.4. General Depression Scale (Allgemein Depression Skala)—Long Form (ADS-L)

ADS-L identifies the presence and severity of depressive symptoms [33,34]. It includes 20 items, and its score is determined by adding the responses to each item, with a range of 0 to 3. The higher the score, the greater the severity of depressive symptoms. The ADS-L has a cut-off value for depressive disorders of ≥22.

### 2.5. Generalized Anxiety Disorder 7 (GAD-7)

GAD-7 is a concise self-report questionnaire utilized for screening and evaluating the severity of symptoms associated with generalized anxiety disorder (GAD) [35]. It consists of seven questions with closed answers, each ranked on a scale of 0 to 3 points. The results of 0–4 points indicate minimal anxiety; 5–9 points, mild anxiety; 10–14 points, moderate anxiety; and 14–21 points, severe anxiety.

### 2.6. Perceived Stress Questionnaire (PSQ)

The PSQ was developed in 1993 [36] and validated in German in 2005 [37]. It consists of four subscales (worries, tension, joy, and demands) and 30 items, each with four possible answers, scored from 1 to 4. Higher scores indicate greater levels of stress.

### 2.7. Tinnitus Questionnaire (TQ)

TQ was created in 1988 [38] and adopted in Germany in 1992 [39]. TQ assesses how tinnitus affects quality of life and includes six subscales: cognitive and emotional distress, intrusiveness, auditory perceptual difficulties, sleep disturbances, and somatic complaints. The TQ score ranges from 0 to 84, with a cut-off value of 47, indicating unhabituated (decompensated) tinnitus.

### 2.8. Statistical Analyses and Graphical Presentation

The statistical analyses were performed with the IBM SPSS Statistics version 29.0 (IBM Deutschland GmbH, Böblingen, Germany). Because most of the data exhibited a non-normal distribution, as indicated by the Kolmogorov–Smirnov test, the nonparametric Wilcoxon paired sample test was employed to assess the direction and significance of the pre-post changes. To calculate the effect size for a Wilcoxon test, the absolute value of the *Z*-statistic was divided by the square root of the sample size and reported as an “*r*” value. The effect size estimates for *r* were as follows: *r* < 0.10, no effect; *r* = 0.10–0.30, small effect size; *r* = 0.3–0.50, medium effect size; *r* = 0.50–0.70, large effect size; *r* > 0.70, very large effect size [40]. To identify correlations between variables, a non-parametric Spearman rho test was employed. Multiple linear regression was applied to determine the predictors of NCIQ in the entire sample. Due to the large sample size, despite the non-normal data distribution, this type of regression is permitted [41]. A quantile regression analysis, which does not assume the normal data distribution, was conducted to identify the factors predicting health-related quality of life within the 0.25, 0.5, and 0.75 quantiles of the NCIQ. The plots and graphics were generated using Biorender.com.

## 3. Results

### 3.1. Speech Intelligibility Improves Significantly After One Year of Using CI

**Figure 1:**
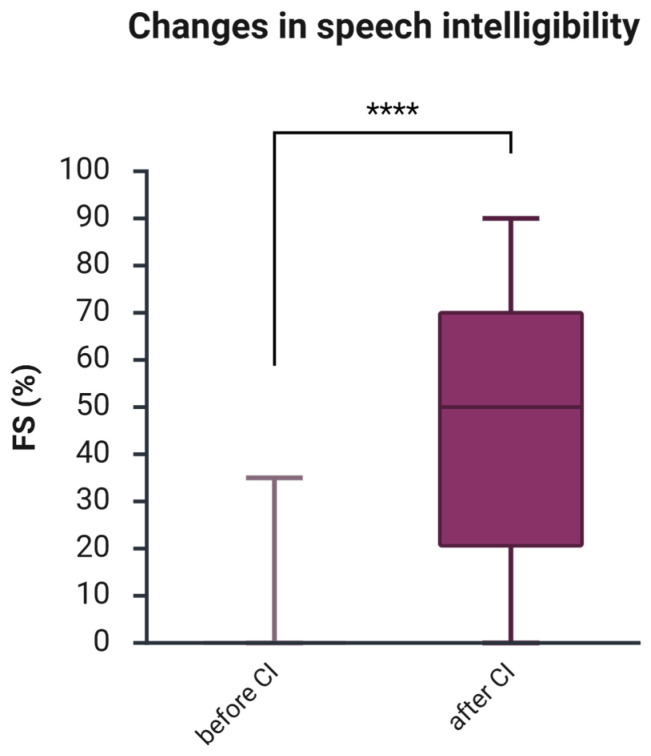
Changes in speech intelligibility one year after CI. The figure displays boxplots with the median as a line inside the box, and the 5th and 95th percentiles as the ends of the whiskers. The Wilcoxon signed-rank test was used to determine if there is a significant difference between paired samples before and after CI. FS, Freiburg Monosyllabic Test; CI, cochlear implant; ****, *p* < 0.001.

The average speech intelligibility measured by FS before CI was 4.9% (Table 1). One year after CI, FS increased to an average of 46.3% (SD 28.4). A Wilcoxon signed-rank test was conducted to compare the FS scores before and one year after CI. The median score before CI was 0, and after CI, 50 (Figure 1). The test indicated a significant difference between the groups (z = −8.86, *p* = 8.1596 × 10^−19^, *r* = −0.57—a large effect size), indicating a clinically significant, notably higher median score after CI.

A separate analysis conducted within the three subgroups of various hearing loss revealed that speech intelligibility significantly improved after cochlear implantation (CI) in all three groups of hearing loss (Table 2). The effect size was large for the DSD and SSD groups and medium for the AHL group.

### 3.2. Improvements in the Subjectively Assessed Hearing Ability (OI)

**Figure 2:**
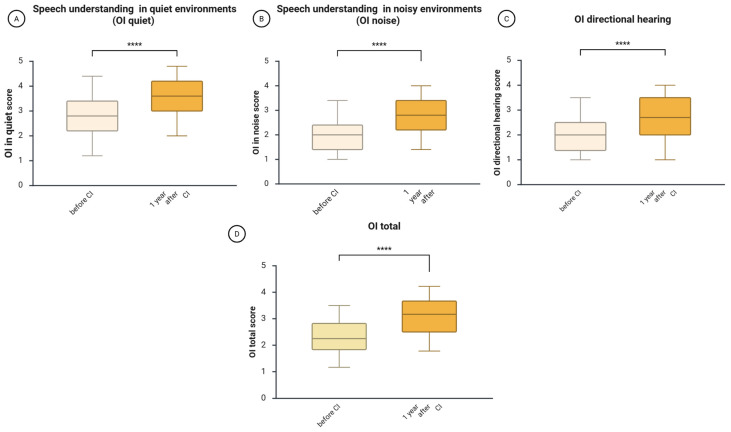
Changes in subjectively assessed hearing ability (OI) one year after CI. The figure displays boxplots with the median as a line inside the box, and the 5th and 95th percentiles as the ends of the whiskers. (**A**), changes in OI quiet (speech understanding in quiet); (**B**), changes in OI noise (speech understanding in noisy environment); (**C**), changes in OI directional hearing; (**D**), changes in the total OI score. The Wilcoxon signed-rank test was used to determine if there is a significant difference between paired samples before and after CI. OI, Oldenburger Inventory; CI, cochlear implantation; ****, *p* < 0.001.

One year after CI, there was a notable improvement in the self-assessed ability to understand speech in quiet environments (OI quiet), understand speech in noisy environments (OI noise), and identify the direction of a sound source (OI directional hearing). In detail, the Wilcoxon test showed that 1 year after CI, the median score of understanding speech in quiet increased from 2.8 before CI to 3.6 after CI (z = −10.14, *p* = 3.5164 × 10^−24^, *r* = 0.48—medium effect size, Figure 2A); understanding speech in noisy environments increased from 2.0 to 2.8 (z = −10.44, *p* = 1.6534 × 10^−25^, *r* = −0.49—medium effect size, Figure 2B); the median directional hearing improved from 2.0 to 2.7 (z = −8.77, *p* = 1.7895 × 10^−18^, *r* = −0.41—medium effect size, Figure 2C); and the whole score of the Oldenburg Inventory rose from 2.25 to 3.15 7 (z = −11.01, *p* = 3.5681 × 10^−28^, *r*= −0.52—large effect size, Figure 2D).

Analysis of the hearing loss subgroups (AHL, DSD, SSD) also showed significant improvements in subjective hearing across all groups (Table 3). The effect sizes ranged from medium to large, with medium effects in the AHL group, predominantly large in the DSD group, and mixed in the SSD group.

### 3.3. The Health-Related Quality of Life Increases Significantly One Year After CI

**Figure 3:**
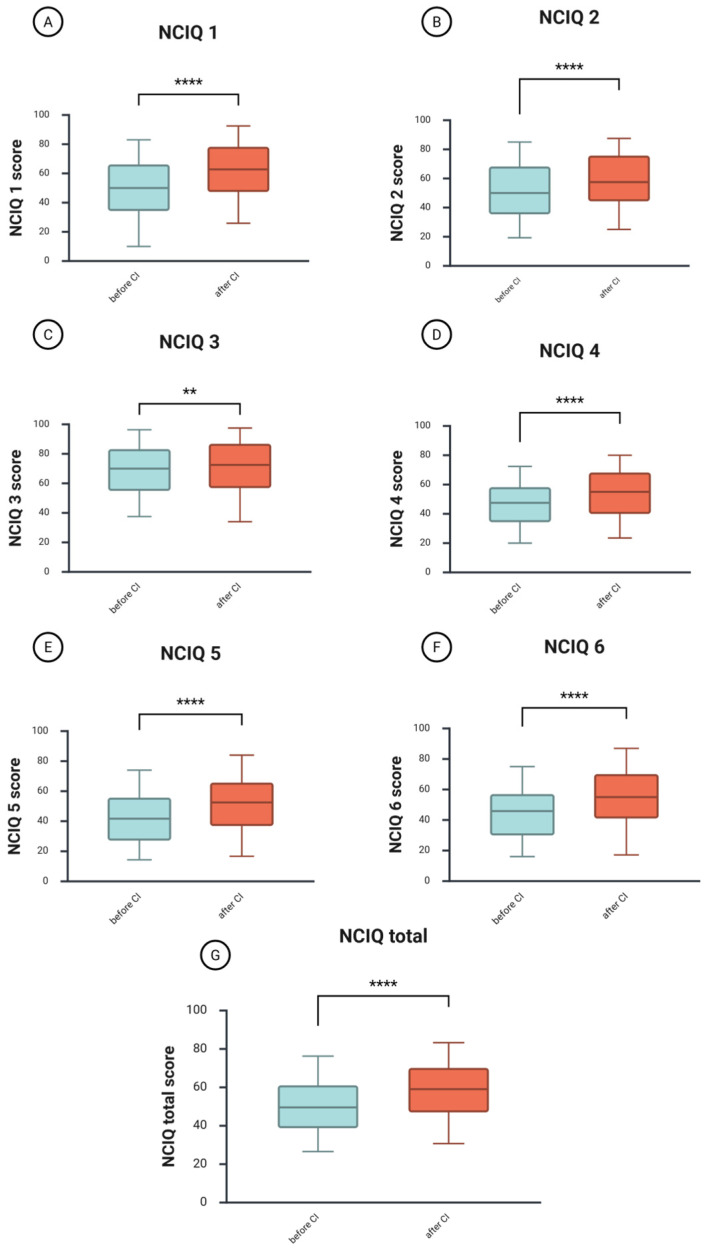
Changes in the health-related quality of life one year after CI. (**A**), changes in NCIQ1 (basic sound perception); (**B**), changes in NCIQ2 (advanced sound perception); (**C**), changes in NCIQ3 (speech production); (**D**), changes in NCIQ4 (self-esteem); (**E**), changes in NCIQ5 (social activities); (**F**), changes in NCIQ6 (social interactions); (**G**), changes in the total score of NCIQ. The figure displays boxplots with the median as a line inside the box, and the 5th and 95th percentiles as the ends of the whiskers. The Wilcoxon signed-rank test was used to determine if there is a significant difference between paired samples before and after CI. NCIQ, Nijmegen Cochlear Implant Questionnaire; CI, cochlear implantation; ****, *p* < 0.001; **, *p* < 0.01.

A Wilcoxon signed-rank test was conducted to compare the NCIQ scores before and one year after CI (Figure 3). The total median NCIQ score before CI was 50, and after CI, 59.4 (Figure 3G). The test indicated a significant difference between the groups (z = −7.39, *p* = 4.4508 × 10^−15^, *r* = −0.35—medium effect size), suggesting a significantly higher score after CI.

Improvements were observed across all areas of patients’ health-related quality of life. The physical domain includes basic sound perception (NCIQ1, Figure 3A), with a median score before CI of 50 and an increase to 62.8 after CI. The difference between the groups was significant (z = −7.87, *p* = 3.6186 × 10^−15^, *r* = −0.37—medium effect size). The median score for advanced sound perception (NCIQ2, Figure 3B) changed from 50 before CI to 57.5 after CI (z = −5.94, *p* =2.9343 × 10^−9^, *r* = –0.28—a small effect size). Finally, the speech production (NCIQ3, Figure 3C) improved from 70 to 72.5, with this small improvement being significant (z = −2.91, *p* < 0.004, *r* = −0.14—a small effect size).

The psychological domain includes self-esteem (NCIQ4, Figure 3D), which increased from a median score of 47.5 before CI to 55.0 after CI (z = −6.38, *p* = 1.7841 × 10^−10^, *r* = −0.30—medium effect size).

The social domain includes limitations in social activities (NCIQ5, Figure 3E) and social interactions (NCIQ6, Figure 3F). Both improved, with NCIQ5 increasing from 41.7 to 52.5 after CI (z = −6.76, *p* = 1.4285 × 10^−11^, *r* = −0.32—medium effect size) and NCIQ6 increasing from 45.83 to 55.0 after CI (z = −7.17, *p* = 7.6916 × 10^−13^, *r* = −0.34—medium effect size).

Analyses in subgroups revealed that the DSD group had the largest benefit in terms of health-related quality of life, followed by the AHL group, whereas the SSD patients showed the smallest improvements (Table 4). However, a between-group analysis (Kruskal–Wallis test) before CI indicated that the SSD patients had significantly higher scores in almost all domains of the NCIQ before implantation, whereas the DSD group had the lowest scores (Supplementary S1).

### 3.4. Influence of CI on Depressive and Anxiety Symptoms and on the Perceived Stress Level

Although the median depressive symptoms score ADS-L (14) remained unchanged one year after implantation, it showed a small yet significant decrease (z = −2.66, *p* = 0.0079, *r* = −0.13), indicating a small effect size. Likewise, the median value of anxiety symptoms, as measured by the GAD-7, declined slightly but significantly from 5 to 4 (z = −2.23, *p* = 0.0261, *r* = −0.11—a small effect size).

The median PSQ score before CI (0.33) changed slightly after CI (0.32), z = −2.04, *p* = 0.042, *r* = −0.09), indicating no effect.

There were no differences between the groups in terms of scores before and after CI (Supplementary S2).

### 3.5. Decrease in Tinnitus-Related Distress

**Figure 4:**
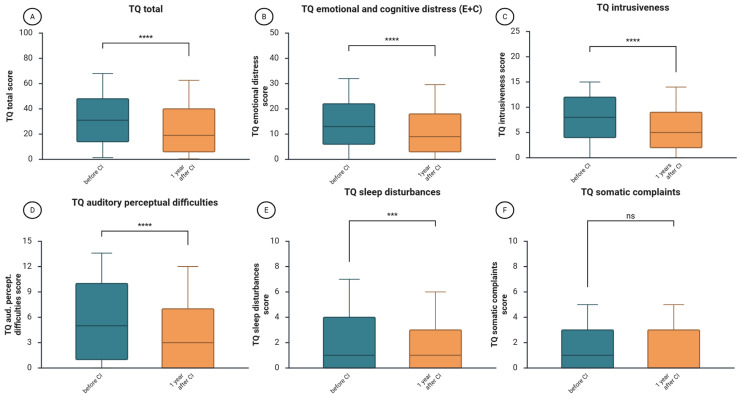
Changes in tinnitus-related distress one year after CI. (**A**), changes in the TQ total score; (**B**), changes in tinnitus-related cognitive and emotional distress; (**C**), changes in tinnitus intrusiveness; (**D**), changes in tinnitus-induced auditory perceptional difficulties; (**E**), changes in tinnitus-related sleep disturbances; (**F**), changes in tinnitus-related somatic complaints. The figure displays boxplots with the median as a line inside the box, and the 5th and 95th percentiles as the ends of the whiskers. The Wilcoxon signed-rank test was used to determine if there is a significant difference between paired samples before and after CI. TQ, Tinnitus Questionnaire; CI, cochlear implantation; ****, *p* < 0.001, ***, *p* < 0.01; ns, not significant.

The median value of the total score of TQ, indicating the general level of tinnitus-related distress, decreased significantly from 31 before CI to 19 points one year after CI (*z* = −6.45, *p* = 1.1366 × 10^−10^, *r* = −0.30—medium effect size, Figure 4A). The scores of individual TQ subscales also decreased, indicating improvement of cognitive and emotional distress (*z* = −5.55, *p* = 9.5089 × 10^−9^, *r* = 0.27, Figure 4B), decrease of tinnitus intrusiveness (*z* = −6.89, *p* = 5.5674 × 10^−12^, *r* = 0.32, Figure 4C), reduction in auditory perceptual difficulties (*z* = −5.64, *p* = 1.7164 × 10^−8^, *r* = 0.26, Figure 4D), and lower level of sleep disturbances difficulties (*z* = −3.98, *p* = 0.000120398154684, *r* = 0.18, Figure 4E). No significant change was noted in the subscale “somatic complaints” (*z* = 1.28, *p* = 0.201107832471672).

The analysis of tinnitus-related distress in individual hearing loss groups (AHL, DSD, SSD) showed differences in effect sizes between groups (Table 5). There were no significant differences between the groups before or after CI in terms of subdomain or total scores (Supplementary S3). There was a medium effect size regarding the emotional and cognitive impact of tinnitus (TQEC), tinnitus intrusiveness (TQ I), and auditory perceptual difficulties induced by tinnitus (TQ A) in the AHL and SSD groups, but in the DSD group, that effect was small.

### 3.6. The Health-Related Quality of Life in CI Patients Correlates with the Subjective Hearing Abilities (OI) and the Severity of Tinnitus-Related Distress (TQ)

In the next step of our analysis, we used the Spearman non-parametric correlation to examine the relationship between the total scores from all questionnaires, namely NCIQ, OI, PSQ, ADS-L, GAD-7, and TQ. Two time points were analyzed: before and one year after CI. The analysis at the first time point revealed several relationships between NCIQ and the other variables (Table 6); however, most of these relationships were negative and weak (ranging from 0.20 to 0.29). One moderate negative correlation was found between NCIQ and the depressive symptoms ADS-L. Additionally, a very strong, positive, and significant relationship was observed between NCIQ and OI.

Analysis of variables one year after CI revealed an increase in the strength of the previously observed relationships, with the direction of the associations remaining unchanged (Table 7). The correlation between NCIQ and ADS-L indicated a strong relationship, and that between NCIQ and GAD, PSQ, and TQ—a moderate relationship. The correlation between OI and NCIQ remained unchanged (very strong relationship).

The correlation pattern among the AHL, DSD, and SSD groups was largely consistent with that of the entire sample (Supplementary S4).

### 3.7. Predictors of Health-Related Quality of Life for CI Patients

First, we used a multiple linear regression to identify the impact of the variables studied on the health-related quality of life (HRQL) measured before and after auditory rehabilitation with CI. The following hypotheses were proposed:

**H1.**  *There is a significant positive impact of subjective hearing (OI) on the NCIQ score*.

**H2.**  *There is a significant negative impact of depressive symptoms (ADSL) on the NCIQ score*.

**H3.**  *There is a significant negative impact of anxiety (GAD) on the NCIQ score*.

**H4.**  *There is a significant negative impact of the perceived stress (PSQ) on the NCIQ score*.

**H5.**  *There is a significant negative impact of tinnitus-related distress (TQ) on the NCIQ score*.

The dependent variable (NCIQ) was regressed on predictor variables, including OI, ADLS, GAD, OSQ, and TQ.

Before implantation, the only variable that significantly affected the NCIQ was the OI (F(5, 217) = 65.32, *p* < 0.001, R^2^ = 0.60; B = 14.526, t = 16.535, *p* < 0.001), confirming hypothesis H1. The other hypotheses (H2–H5) were not supported for the time point before CI.

One year after auditory rehabilitation with CI, the independent variables significantly predicted health-related quality of life, F(5, 215) = 74.57, *p* < 0.001, with R^2^ = 0.634, indicating that the model explains 63.4% of the variance in NCIQ. Additionally, coefficients were examined to assess the impact of each variable on NCIQ. The results showed that subjective hearing (OI) has a significantly positive effect on NCIQ (B = 13.229 (standardized β = 0.620), t = 13.175, *p* < 0.001), thus confirming hypothesis H1. The depressive symptoms had a significantly negative effect on NCIQ (B = −0.214, t = −1.968, *p* = 0.05), supporting hypothesis H2. Hypotheses H3 and H4 were not supported by the regression results, thus eliminating anxiety and perceived stress as possible predictors. Finally, there was a significant negative effect of TQ on NCIQ (B = −0.112, t = −2.737, *p* = 0.007), confirming Hypothesis H5.

To further clarify how predictors influence different parts or quantiles of a response variable’s (NCIQ) distribution, we used quantile regression (QR) and estimated the coefficients of predictor variables across various NCIQ outcome quantiles (Table 8). The pseudo R-squared values indicated a good fit (0.411 for the 25th, 0.424 for the median, and 0.449 for the 75th percentile). The results showed a strong positive impact of OI (subjective assessment of hearing ability) across the entire sample, regardless of the NCIQ level. Tinnitus-related distress (TQ) negatively affected the NCIQ in the 0.25 and 0.5 quantiles, but was no longer impacting the NCIQ in the 0.75 quantile. Moreover, depressive symptoms (ADSL) had a negative effect on patients with the lowest NCIQ scores.

## 4. Discussion

This study explored the changes in speech intelligibility, health-related quality of life, tinnitus-related distress, depressive symptoms, anxiety, and perceived stress one year after cochlear implantation in a cohort of 227 adult CI patients. We found that after one year of auditory rehabilitation with CI, there was a significant improvement in speech intelligibility across the entire cohort (Figure 1) and within the subgroups based on hearing loss type (Table 2). Additionally, the total score of the self-assessment OI test and each of its subdomains showed significant improvement in the cohort (Figure 2) and subgroups (Table 3), consistent with the results of a systematic review that examined auditory rehabilitation outcomes related to sound quality and speech understanding using OI and other self-report tests [42]. Furthermore, health-related quality of life improved (Figure 3), while depressive and anxiety symptoms decreased, and tinnitus-related distress was reduced (Figure 4). Finally, we observed a positive effect of speech intelligibility as measured by OI, and a negative effect of tinnitus-related distress and depressive symptoms on the NCIQ.

Our cohort’s health-related quality of life, as measured by the NCIQ, demonstrated significant improvements across all domains and the overall score one year after CI (Figure 3), aligning with results from earlier studies that employed this measure [42]. The only study in which no significant improvement was observed in one domain (NCIQ3—speech production) is our own study conducted with a group of 17 patients aged 80 and over [43]. We explained this by the relatively high NCIQ3 scores before CI in this particular group of patients. Subgroup analyses by hearing loss type confirmed overall improvements in the NCIQ score, with variations in significance and effect size across subscales (Table 4). In SSD, no significant gains were observed in basic and advanced sound perception (NCIQ1, NCIQ2) or speech production (NCIQ3). However, improvements were noted in self-esteem (NCIQ4), social interactions (NCIQ5), and activities (NCIQ6). The AHL group reported no effect on NCIQ3, NCIQ4, and NCIQ5. Our results partially align with those of Lassaletta et al., who found CI-related improvements in all NCIQ subscales for the AHL patients after one year, with notable gains in basic sound perception (NCIQ1) and social activities (NCIQ6), but no change in self-esteem (NCIQ4) among SSD patients [44]. However, there were fewer patients who completed the NCIQ in the study by Lassaletta et al., possibly accounting for the differences observed (SSD, 12—our study, 43; AHL, 19, our study, 58).

Our cohort’s estimated depressive symptoms (ADS-L) were mild, with a median score of 14, well below the cutoff of 22, indicating no depressive disorder. Although a significant decrease was observed in the entire cohort one year after rehabilitation with CI, the effect size was very small. We noted a similar trend regarding the anxiety symptoms (GAD-7). A recent study using the Hospital Anxiety and Depression Scale (HADS) to compare depression and anxiety scores between 53 CI users and a control group matched by age and sex (without hearing impairment) found no significant differences between the groups [45], supporting our finding. In another study using the HADS, the authors noted a decrease in depression scores one year after CI; however, three years after CI, the scores returned to baseline [46]. Moreover, the present results showing no significant changes in ADS-L and GAD-7 after CI agree with our previously published studies focusing on patients with asymmetric hearing loss [29], single-sided deafness [47], or an older group with bilateral hearing loss [43]. However, in another group of younger patients (average age 57.63 years) with bilateral hearing loss, we did observe a small but significant decrease in anxiety (but not depressive) symptoms after the first implantation [15], but the score returned to baseline after the second CI. Taken together, cochlear implant auditory rehabilitation shows little or only short-term benefits for anxiety and depression.

The questionnaire used to assess perceived stress level (PSQ) measures worries about the future, difficulty relaxing, reduced pleasure, and feeling overwhelmed, and is not directly linked to a health condition. A median PSQ score of 0.33 shows that patients experienced moderate stress levels before their CI surgery and continued to do so (0.32) one year later (size effect below 0.1, indicating no effect). Notably, after one year, the correlation between PSQ and other variables remained in the same direction, although the strength of these relationships increased. The negative correlation between PSQ and NCIQ was minimal before CI and became low-positive after, indicating that the association between the ranked variables is strengthening. The negative correlation between PSQ and OI followed the same trend. Conversely, the strong positive correlations between PSQ and ADS-L or GAD shifted to moderately positive after implantation, suggesting a weakening of these associations. A similar pattern was observed with TQ, which showed a moderate positive correlation with PSQ before surgery; however, this coefficient became insignificant after CI. Therefore, it can be concluded that auditory rehabilitation with CI alters the strength of the relationship between perceived stress and other variables. The lack of changes between pre- and post-CI PSQ scores has been previously observed [43,48], and the decrease in perceived stress was also observed [49], clearly depending on the baseline value of the PSQ score being 0.48 in that group of patients and decreasing to the same level observed in the present study (0.33). One can explain the discrepancies in these results by noting that, in the first decade of this century (the time when the study [48] was performed), cochlear implantation was not a routine type of surgery as it is today, and the patients who qualified for it might have been under higher psychological pressure than nowadays.

The presence of tinnitus and tinnitus-related distress in the CI patients has been a topic of intense research. In our cohort, there is a significant decrease in the total TQ score one year after CI rehabilitation, dropping from a median of 31 to 19. This decline is clinically meaningful, as research shows that a 5-point decrease in the TQ score is considered significant [50]. Moreover, another study suggested that a 12-point change is also clinically relevant [51], and our results are consistent with this. This is in agreement with our previously published data [26,49,52] and the numerous data from other research groups [11,19,53,54]. A recent meta-analysis of research involving patients with SSD found that, after auditory rehabilitation with a CI, most patients experienced a significant reduction in tinnitus-related distress by 11.66 points according to the Tinnitus Handicap Inventory (THI) [55]. In another mixed (qualitative/quantitative) study of 414 patients with CIs, the tinnitus-silencing effect was found to depend on having a switched-on CI sound processor, but only in 30% of the patients [56]. Importantly, with the sound processor on, most implanted patients reported that their tinnitus was not a problem or only a minor problem, while only 10% considered it a serious issue. Regardless of the mechanism suppressing the tinnitus (restoring auditory deprivation, electrical stimulation of auditory neurons, or both), these results support our findings and highlight the beneficial effect of auditory rehabilitation with CI on tinnitus.

The final part of our analysis involved correlations and regressions to assess the direction, strength, and predictive value of individual variables on the health-related quality of life in CI patients. The correlation analyses revealed a strong positive relationship between NCIQ and OI, both before (Table 2) and after CI (Table 3). There was also a weak negative correlation between NCIQ and ADS-L before CI, which increased to a moderate level after CI. The remaining correlation coefficients were negligible. After CI, a weak negative correlation appeared between NCIQ and TQ. To better understand the relationship between variables and predict outcomes by modeling how changes in one or more variables (independent variables) influence the dependent variable NCIQ, we performed regression analyses using both linear and quantile regressions. We have identified OI as a strong positive predictor of NCIQ after CI, together with two negative predictors: TQ and ADSL. Importantly, subjective hearing (OI) influenced all NCIQ quantiles equally, whereas TQ affected the 0.25 and 0.5, but not the highest quantile. ADSL affected only the lower quantile and had no effect on the median and the highest quantile of NCIQ. The clinical significance of our findings is that subjective hearing is heteroscedastic, as it consistently and significantly impacts health-related quality of life, regardless of whether it is low, median, or high. To enhance the quality of life for all CI patients, clinicians should consider not only audiometric results but also the patients’ personal opinions regarding their hearing abilities. In contrast, patients with a low or average quality of life after CI should be specifically evaluated for tinnitus-related distress and depressive symptoms, as they may benefit from psychological counseling.

**Figure 5:**
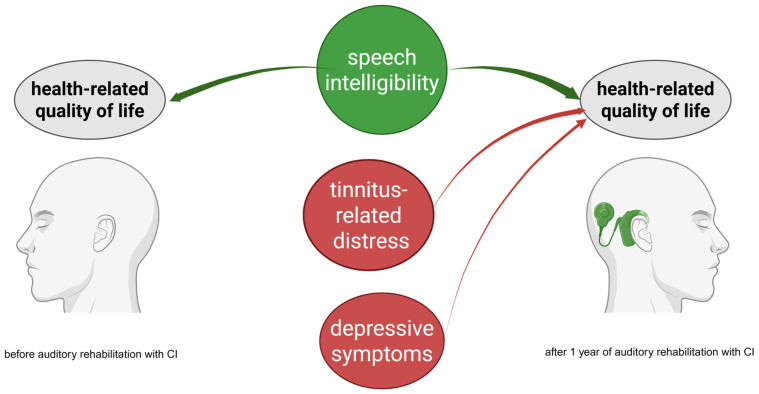
Schematic representation of variables influencing health-related quality of life (NCIQ) before (**left**) and after (**right**) auditory rehabilitation with CI. The subjective, patient-reported outcome of speech comprehension/intelligibility OI positively impacts the quality of life before and after CI. Tinnitus-related distress (TQ) and depressive symptoms (ADS) have a negative impact on NCIQ but only after CI. Red indicates a negative impact, green indicates a positive impact.

Overall, our analysis indicates that one year of auditory rehabilitation with cochlear implants has a positive impact on speech intelligibility (FS and OI) and health-related quality of life, as measured by the NCIQ (Figure 5). It also reduces the discomfort caused by tinnitus. This information is valuable when advising patients before and during auditory rehabilitation. It is also important to note that although the levels of depression and anxiety were low among the cohort and the reduction in depressive and anxiety symptoms was modest, depressive symptoms had a significant effect on the NCIQ after implantation in the group of patients with a low NCIQ (0.25 quantile). Importantly, tinnitus has a negative impact on patients with low and median NCIQ scores. This indicates that tinnitus and depressive symptoms should be monitored during auditory rehabilitation with CI, and the information collected should guide audiological and psychological interventions.

Although our study collected and analyzed data on a large group of patients with cochlear implants, research of this kind conducted within CI registries [57,58,59,60] could offer more information on NCIQ predictors. Therefore, it is advisable that, in the future, anonymized data from individual institutions be incorporated into these registries.

Our study has limitations. The first is the uneven distribution of patients with AHL, DSD, and SSD in our sample: more than half had bilateral hearing loss, while the rest had unilateral or asymmetric hearing loss. This distribution of different types of hearing loss could potentially skew the results toward those more typical of bilateral deafness. The second limitation is that we used tests specific to German-speaking countries (e.g., OI or the German version of TQ), which makes it difficult to compare our findings with those from studies using other outcome measures. Introducing internationally accepted, language- and country-validated outcome measures into our test battery would change this situation; however, it would also cause discontinuity in our database.

Another limitation of this study is the heterogeneity of the devices used during the study period. Technological developments occurred during the study period, resulting in differences in speech processor front-end processing and stimulation strategies. However, recent analyses of large patient cohorts have shown stable mean postoperative speech comprehension values in quiet and in noise since 2012 [61]. Moreover, although the rehabilitation process at our tertiary center and affiliated rehabilitation centers was standardized, it was not always identical; rather, it was tailored to each patient’s specific needs. This could also have influenced the outcomes measured in our study.

The final limitation of this study is that we did not account for confounding variables, such as the duration of hearing loss, the intensity of the rehabilitation process, socioeconomic status, and comorbid conditions, in our analysis. A recent study of adult CI recipients showed that socioeconomic status (employment status and household income) was associated with improved health-related quality of life [62]. We had no information on both variables, but we plan to include it in our future studies. The same study has shown that a longer duration of hearing loss is positively associated with a better quality of life after implantation—a finding that surprised the authors. We only had data on hearing loss duration from 173 patients (Table 1), so we excluded this variable from our analysis to prevent a significant reduction in sample size. Finally, our database did not record any comorbidities besides tinnitus, depression, and anxiety symptoms. Lee et al. found that comorbid conditions such as cancer or cardiac issues in CI recipients are linked to poorer audiological performance one year after implantation [63] but seemed not to affect the quality of life. However, the sample size in that study was small (28 patients), which may have influenced the study results. The mentioned limitations will be addressed in the future by expanding the database to include the listed variables.

## 5. Conclusions

This study found that a large, diverse group of adult patients who underwent auditory rehabilitation with CI benefited not only through improved hearing measures but also by experiencing lower levels of psychological comorbidities. Importantly, the depressive symptoms negatively affect patients’ quality of life, especially for those who perceive it as low, in the 0.25 quantile of NCIQ. Furthermore, tinnitus-related distress also harms the quality of life of patients in the first and median quartiles of NCIQ. These findings underscore the importance of ongoing monitoring of parameters such as ADS-L and TQ, as well as the necessity for targeted clinical interventions during the rehabilitation process.

## Figures and Tables

**Table 1:** Demographic and auditory characteristics of study participants.

Variable	Mean	*n*	Min–Max	SD
Age at the surgery (years)	61.1	227	18–86	14.0
Duration of deafness (years)	17.0	173	0–72	20.5
Speech intelligibility (FS) before CI in the ear scheduled for implantation (%)	4.9	176	0–55	11.1

*n*, number of patients; SD, standard deviation.

**Table 2:** Changes in speech intelligibility among subgroups AHL, DSD, and SSD one year after CI, calculated with the Wilcoxon signed-rank test.

AHL				DSD				SSD			
Z	*p* (2-Tailed)	*r*	Effect Size Estimates	Z	*p* (2-Tailed)	*r*	Effect Size Estimates	Z	*p* (2-Tailed)	*r*	Effect Size Estimates
−3.986	6.72043 × 10^−5^	−0.46	medium	−7.254	4.04 × 10^−13^	−0.55	large	−3.062	0.0022	−0.50	large

Z, the degree of difference between the groups; *p*, asymptotic significance; *n*, sample size; *r*, size effect.

**Table 3:** Improvement in subjective hearing among subgroups AHL, DSD, and SSD one year after CI, calculated with the Wilcoxon signed-rank test.

		OI Silence	OI Noise	OI Directional	OI Total
AHL	Z	−4.391	−4.794	−3.584	−4.932
	*p* (2-tailed)	1.13061 × 10^−5^	1.63647 × 10^−6^	0.000338928	8.15 × 10^−7^
	*n*	58			
	*r*	−0.41	−0.45	−0.33	−0.46
	effect size estimates	medium	medium	medium	medium
DSD	Z	−8.215	−7.733	−6.553	−8.407
	*p* (2-tailed)	2.1287 × 10^−16^	1.0468 × 10^−14^	5.6413 × 10^−11^	4.1998 × 10^−17^
	*n*	119			
	*r*	−0.53	−0.5	−0.42	−0.55
	effect size estimates	large	large	medium	large
SSD	Z	−3.308	−4.933	−4.303	−4.792
	*p* (2-tailed)	0.000940993333402	8.0815 × 10^−7^	0.000016834729178	0.00000164827778
	*n*	42			
	*r*	−0.36	−0.54	−0.47	−0.52
	effect size estimates	medium	large	medium	large

Z, the degree of difference between the groups; *p*, asymptotic significance; *n*, sample size; *r*, size effect.

**Table 4:** Improvement in the health-related quality of life among subgroups AHL, DSD, and SSD one year after CI, calculated with the Wilcoxon signed-rank test.

		NCIQ1 Basic Sound Perception	NCIQ2 Advanced Sound Perception	NCIQ3 Speech Production	NCIQ4 Self-Esteem	NCIQ5 Social Activities	NCIQ6 Social Interactions	NCIQ Total Score
AHL	Z	−3.677	−2.834	−0.649	−1.546	−1.13	−2.207	−2.93
	*p* (2-tailed)	0.000236	0.0046	0.516558	0.122027	0.258508	0.02734	0.003384
	*n*	58						
	*r*	−0.34	−0.26				−0.2	−0.28
	effect size estimates	medium	medium				small	small
DSD	Z	−6.829	−5.518	−2.978	−6.025	−6.562	−6.201	−6.977
	*p* (2-tailed)	8.52 × 10^−12^	3.43 × 10^−8^	0.002904	1.69 × 10^−9^	5.3 × 10^−11^	5.59 × 10^−10^	3.02 × 10^−12^
	*n*	119						
	*r*	−0.44	−0.35	−0.19	−0.39	−0.42	−0.4	−0.45
	effect size estimates	medium	medium	small	medium	medium	medium	medium
SSD	Z	−1.42	−0.5	−0.773	−2.145	−2.625	−3.479	−2.427
	*p* (2-tailed)	0.155591	0.61739	0.439519	0.031979	0.008675	0.000503	0.015222
	*n*	43						
	*r*				−0.23	−0.28	−0.37	−0.26
	effect size estimates				small	small	medium	small

NCIQ, Nijmegen Cochlear Implant Questionnaire; NCIQ1, basic sound perception; NCIQ2, advanced sound perception; NCIQ3, speech production; NCIQ4, self-esteem; NCIQ5, social activities; NCIQ6, social interactions; Z, the degree of difference between the groups; *p*, asymptotic significance; *n*, sample size; *r*, size effect.

**Table 5:** Changes in tinnitus-related distress among subgroups AHL, DSD, and SSD one year after CI, calculated with the Wilcoxon signed-rank test.

		TQ EC	TQ I	TQ A	TQ SI	TQ SO	TQ Total
AHL	Z	−3.635	−4.104	−3.678	−2.136	−1.405	−4.023
	*p* (2-tailed)	0.000278	4.06 × 10^−5^	0.000235	0.032649	0.160069	5.74 × 10^−5^
	*n*	58					
	*r*	−0.34	−0.38	−0.34	−0.20		−0.37
	effect size estimates	medium	medium	medium	small		medium
DSD	Z	−3.197	−3.743	−2.827	−2.312	−1.092	−3.429
	*p* (2-tailed)	0.00139	0.000182	0.004693	0.020753	0.275045	0.000605
	*n*	119					
	*r*	−0.2	−0.24	−0.18	−0.15		−0.22
	effect size estimates	small	small	small	small		small
SSD	Z	−3.051	−4.096	−4.046	−1.987	−0.197	−4.047
	*p* (2-tailed)	0.002284	4.21 × 10^−5^	5.21 × 10^−5^	0.046969	0.843488	5.2 × 10^−5^
	*n*	43					
	*r*	−0.33	−0.44	−0.44	−0.21		−0.44
	effect size estimates	medium	medium	medium	small		medium

TQ, Tinnitus Questionnaire; TQ EC, tinnitus-related cognitive and emotional distress; TQ I, tinnitus intrusiveness; TQ A, tinnitus-induced auditory perceptual difficulties; TQ Sl, tinnitus-related sleep disturbances; TQ, SO, tinnitus-related somatic complaints; Z, the degree of difference between the groups; *p*, asymptotic significance; *n*, sample size; *r*, size effect.

**Table 6:** Non-parametric Spearman correlations between the variables before implantation.

		NCIQ	OI	ADSL	GAD	PSQ	TQ
NCIQ	Correlation Coefficient	1					
	Sig. (2-tailed)	.					
	N	227					
OI	Correlation Coefficient	0.747 **	1				
	Sig. (2-tailed)	<0.001	.				
	N	227	227				
ADSL	Correlation Coefficient	−0.317 **	−0.213 **	1			
	Sig. (2-tailed)	<0.001	0.001	.			
	N	225	225	225			
GAD	Correlation Coefficient	−0.240 **	−0.107	0.700 **	1		
	Sig. (2-tailed)	<0.001	0.111	<0.001	.		
	N	225	225	223	225		
PSQ	Correlation Coefficient	−0.274 **	−0.137 *	0.735 **	0.732 **	1	
	Sig. (2-tailed)	<0.001	0.039	<0.001	<0.001	.	
	N	227	227	225	225	227	
TQ	Correlation Coefficient	−0.255 **	−0.183 **	0.459 **	0.443 **	0.450 **	1
	Sig. (2-tailed)	<0.001	0.006	<0.001	<0.001	<0.001	.
	N	227	227	225	225	227	227

**, correlation is significant at the 0.01 level (two-tailed); *, correlation is significant at the 0.05 level (2-tailed). Sig, significance; N, number of patients included in the analysis.

**Table 7:** Non-parametric Spearman correlations between the variables one year after auditory rehabilitation with CI.

		1 y NCIQ	1 y OI	1 y ADSL	1 y GAD	1 y PSQ	1 y TQ
1 y NCIQ	Correlation Coefficient	1					
	Sig. (2-tailed)	.					
	N	227					
1 y OI	Correlation Coefficient	0.729 **	1				
	Sig. (2-tailed)	<0.001	.				
	N	226	226				
1 y ADSL	Correlation Coefficient	−0.525 **	−0.410 **	1			
	Sig. (2-tailed)	<0.001	<0.001	.			
	N	223	222	223			
1 y GAD	Correlation Coefficient	−0.373 **	−0.256 **	0.703 **	1		
	Sig. (2-tailed)	<0.001	<0.001	<0.001	.		
	N	226	225	222	226		
1 y PSQ	Correlation Coefficient	−0.466 **	−0.320	0.782	0.728	1	
	Sig. (2-tailed)	<0.001	<0.001	<0.001	<0.001	.	
	N	226	225	222	226	226	
1 y TQ	Correlation Coefficient	−0.444 **	−0.353 **	0.435 **	0.415 **	0.280 **	1
	Sig. (2-tailed)	<0.001	<0.001	<0.001	<0.001	<0.001	.
	N	227	226	223	226	226	227

**. Correlation is significant at the 0.01 level (2-tailed). 1 y, 1 year after cochlear implantation; Sig, significance; N, number of patients included in the analysis.

**Table 8:** Results of quantile regression showing coefficients of the predictor variables at various NCIQ outcome quantiles.

Parameter Estimates by Different Quantiles ^a,b^			
Parameter	q = 0.25	q = 0.5 (Median)	q = 0.75
(Intercept)	23.577 ***	24.346 ***	29.933 ***
OI total score 1 year after CI	12.972 ***	14.569 ***	14.271 ***
ADSL score 1 year after CI	−0.467 *	−0.244	−0.088
GAD score 1 year after CI	−0.274	−0.075	−0.368
PSQ total score 1 year after CI	5.502	−6.567	−10.588
TQ total score 1 year after CI	−0.160 **	−0.153 **	−0.065

^a^. Dependent Variable: NCIQ total score 1 year after CI; ^b^. Model: (Intercept), OI, ADSL, GAD, PSQ, TQ; ***, *p* < 0.001; **, *p* < 0.01, *, *p* < 0.05.

## Data Availability

Data is available from the corresponding author upon reasonable request.

## Supplementary Materials

The following supporting information can be downloaded at: https://www.mdpi.com/article/10.3390/jcm14228143/s1, S1: Kruskal–Wallis Test: the comparison between the pre- and post-scores of NCIQ for AHL, DSD, and SSD groups. S2: Kruskal–Wallis Test: the comparison between the pre- and post-scores of ADSL, GAD and PSQ for AHL, DSD, and SSD groups; S3: Kruskal–Wallis Test: the comparison between the pre- and post-scores of TQ for AHL, DSD, and SSD groups, S4: Nonparametric Correlations in groups AHL, DSD, SSD before and after implantation.

## Abbreviations

The following abbreviations are used in this manuscript:

ADS-L	General Depression Scale (long version questionnaire)
AHL	Asymmetric Hearing Loss
CI	Cochlear Implant
DSD	Double-Sided Deafness (bilateral deafness)
FS	Freiburg Monosyllable Test (speech Intelligibility test)
GAD-7	Generalized Anxiety Disorder 7
NCIQ	Nijmegen Cochlear Implant Questionnaire (health-related quality of life)
OI	Oldenburg Inventory
PSQ	Perceived Stress Questionnaire
SSD	Single-Sided Deafness (unilateral deafness)
TQ	Tinnitus Questionnaire

## References

[1] Chadha S., Kamenov K., Cieza A. (2021). The world report on hearing. Bull. World Health Organ..

[2] Dazert S., Thomas J.P., Loth A., Zahnert T., Stöver T. (2020). Cochlear Implantation. Dtsch. Arztebl. Int..

[3] Zoneff E.R., Gao D.X., Nisbet D.R., Grayden D.B., Clark G.M. (2023). Restoration of the senses and human communication: Sustainable Development Goals 3 and 9. Int. J. Speech Lang. Pathol..

[4] Hinderink J.B., Krabbe P.F.M., van den Broek P. (2000). Development and application of a health-related quality-of-life instrument for adults with cochlear implants: The Nijmegen Cochlear Implant Questionnaire. Otolaryngol. Head Neck Surg..

[5] Vasil K.J., Lewis J., Tamati T., Ray C., Moberly A.C. (2020). How Does Quality of Life Relate to Auditory Abilities? A Subitem Analysis of the Nijmegen Cochlear Implant Questionnaire. J. Am. Acad. Audiol..

[6] Abdrabbou M.F., Tucker D.A., Compton M.V., Mankoff L. (2018). Quality of life and speech perception in two late deafened adults with cochlear implants. Audiol. Res..

[7] Plath M., Marienfeld T., Sand M., van de Weyer P.S., Praetorius M., Plinkert P.K., Baumann I., Zaoui K. (2022). Prospective study on health-related quality of life in patients before and after cochlear implantation. Eur. Arch. Otorhinolaryngol..

[8] Andries E., Gilles A., Topsakal V., Vanderveken O., Van de Heyning P., Van Rompaey V., Mertens G. (2022). The impact of cochlear implantation on health-related quality of life in older adults, measured with the Health Utilities Index Mark 2 and Mark 3. Eur. Arch. Oto-Rhino-Laryngol..

[9] Cuda D., Manrique M., Ramos Á., Marx M., Bovo R., Khnifes R., Hilly O., Belmin J., Stripeikyte G., Graham P.L. (2024). Improving quality of life in the elderly: Hearing loss treatment with cochlear implants. BMC Geriatr..

[10] Baungaard L.H., Sandvej M.G., Nellemose M.M.O., Hestbæk M.K., Brændgaard L.B., Hansen M.S., Jørgensen M.L., Cayé-Thomasen P., Percy-Smith L. (2025). Longitudinal Cochlear Implant Outcomes in Danish Adults: Changes in Speech Recognition, Self-Reported Hearing Ability, Hearing-Related Quality of Life, and Tinnitus. J. Clin. Med..

[11] Yuen E., Ma C., Nguyen S.A., Meyer T.A., Lambert P.R. (2021). The Effect of Cochlear Implantation on Tinnitus and Quality of Life: A Systematic Review and Meta-analysis. Otol. Neurotol..

[12] Gatehouse S., Noble W. (2004). The Speech, Spatial and Qualities of Hearing Scale (SSQ). Int. J. Audiol..

[13] Holube B., Kollmeier B. (1994). Examination of a modified version of a questionnaire to assess subjective hearing handicap and its relation to the Monosyllabic Rhyme Test. Audiol. Akust..

[14] Ramakers G.G.J., Smulders Y.E., van Zon A., Van Zanten G.A., Grolman W., Stegeman I. (2017). Correlation between subjective and objective hearing tests after unilateral and bilateral cochlear implantation. BMC Ear Nose Throat Disord..

[15] Péus D., Pfluger A., Häussler S.M., Knopke S., Ketterer M.C., Szczepek A.J., Gräbel S., Olze H. (2021). Single-centre experience and practical considerations of the benefit of a second cochlear implant in bilaterally deaf adults. Eur. Arch. Oto-Rhino-Laryngol..

[16] Haile L.M., Orji A.U., Reavis K.M., Briant P.S., Lucas K.M., Alahdab F., Bärnighausen T.W., Bell A.W., Cao C., Dai X. (2024). Hearing Loss Prevalence, Years Lived with Disability, and Hearing Aid Use in the United States From 1990 to 2019: Findings From the Global Burden of Disease Study. Ear Hear.

[17] Chen Z., Lu Y., Chen C., Lin S., Xie T., Luo X., Lin Y., Chen Y., Feng Y., Xiong G. (2024). Association between tinnitus and hearing impairment among older adults with age-related hearing loss: A multi-center cross-sectional study. Front. Neurol..

[18] Mazurek B., Olze H., Haupt H., Szczepek A.J. (2010). The more the worse: The grade of noise-induced hearing loss associates with the severity of tinnitus. Int. J. Environ. Res. Public Health.

[19] Quaranta N., Wagstaff S., Baguley D.M. (2004). Tinnitus and cochlear implantation. Int. J. Audiol..

[20] Zhang Z.Q., Li J.Y., Ge S.T., Ma T.Y., Li F.Y., Lu J.L., Si S.R., Cui Z.Z., Jin Y.L., Jin X.H. (2023). Bidirectional associations between sensorineural hearing loss and depression and anxiety: A meta-analysis. Front. Public Health.

[21] Kim H.J., Jeong S., Roh K.J., Oh Y.H., Suh M.J. (2023). Association Between Hearing Impairment and Incident Depression: A Nationwide Follow-up Study. Laryngoscope.

[22] Cacciatore F., Napoli C., Abete P., Marciano E., Triassi M., Rengo F. (1999). Quality of Life Determinants and Hearing Function in an Elderly Population: Osservatorio Geriatrico Campano Study Group. Gerontology.

[23] Li F., Jin M., Ma T., Cui C. (2025). Association between age-related hearing loss and depression: A systematic review and meta-analysis. PLoS ONE.

[24] Shoham N., Lewis G., Favarato G., Cooper C. (2019). Prevalence of anxiety disorders and symptoms in people with hearing impairment: A systematic review. Soc. Psychiatry Psychiatr. Epidemiol..

[25] Chae H., Lee H.S., Lee J.H., Kim M., Park S.Y., Seo Y.J. (2021). Usefulness of stress-related hormones as predictors and prognostic factors for idiopathic sudden sensorineural hearing loss. Acta Otolaryngol..

[26] Olze H., Ketterer M.C., Péus D., Häußler S.M., Hildebrandt L., Gräbel S., Szczepek A.J. (2022). Effects of auditory rehabilitation with cochlear implant on tinnitus prevalence and distress, health-related quality of life, subjective hearing and psychological comorbidities: Comparative analysis of patients with asymmetric hearing loss (AHL), double-sided (bilateral) deafness (DSD), and single-sided (unilateral) deafness (SSD). Front. Neurol..

[27] Häußler S.M., Stankow E., Knopke S., Szczepek A.J., Olze H. (2023). Sustained Cognitive Improvement in Patients over 65 Two Years after Cochlear Implantation. Brain Sci..

[28] Häußler S.M., Knopke S., Wiltner P., Ketterer M., Gräbel S., Olze H. (2019). Long-term Benefit of Unilateral Cochlear Implantation on Quality of Life and Speech Perception in Bilaterally Deafened Patients. Otol. Neurotol..

[29] Ketterer M.C., Knopke S., Häußler S.M., Hildenbrand T., Becker C., Gräbel S., Olze H. (2018). Asymmetric hearing loss and the benefit of cochlear implantation regarding speech perception, tinnitus burden and psychological comorbidities: A prospective follow-up study. Eur. Arch. Otorhinolaryngol..

[30] Knopke S., Szczepek A.J., Häussler S.M., Gräbel S., Olze H. (2017). Cochlear Implantation of Bilaterally Deafened Patients with Tinnitus Induces Sustained Decrease of Tinnitus-Related Distress. Front. Neurol..

[31] Olze H., Knopke S., Gräbel S., Szczepek A.J. (2016). Rapid Positive Influence of Cochlear Implantation on the Quality of Life in Adults 70 Years and Older. Audiol. Neurootol..

[32] Hoth S. (2016). Der Freiburger Sprachtest. HNO.

[33] Meyer T.D., Hautzinger M. (2001). Allgemeine Depressions-Skala (ADS).

[34] Hautzinger M., Bailer M. (1993). Allgemeine Depressions-Skala ADS.

[35] Spitzer R.L., Kroenke K., Williams J.B., Löwe B. (2006). A brief measure for assessing generalized anxiety disorder: The GAD-7. Arch. Intern. Med..

[36] Levenstein S., Prantera C., Varvo V., Scribano M.L., Berto E., Luzi C., Andreoli A. (1993). Development of the Perceived Stress Questionnaire: A new tool for psychosomatic research. J. Psychosom. Res..

[37] Fliege H., Rose M., Arck P., Walter O.B., Kocalevent R.D., Weber C., Klapp B.F. (2005). The Perceived Stress Questionnaire (PSQ) reconsidered: Validation and reference values from different clinical and healthy adult samples. Psychosom. Med..

[38] Hallam R.S., Jakes S.C., Hinchcliffe R. (1988). Cognitive variables in tinnitus annoyance. Br. J. Clin. Psychol..

[39] Hiller W., Goebel G. (1992). A psychometric study of complaints in chronic tinnitus. J. Psychosom. Res..

[40] Maher J.M., Markey J.C., Ebert-May D. (2013). The other half of the story: Effect size analysis in quantitative research. CBE Life Sci. Educ..

[41] Schmidt A.F., Finan C. (2018). Linear regression and the normality assumption. J. Clin. Epidemiol..

[42] Andries E., Gilles A., Topsakal V., Vanderveken O.M., Van de Heyning P., Van Rompaey V., Mertens G. (2020). Systematic Review of Quality of Life Assessments after Cochlear Implantation in Older Adults. Audiol. Neurotol..

[43] Knopke S., Gräbel S., Förster-Ruhrmann U., Mazurek B., Szczepek A.J., Olze H. (2016). Impact of cochlear implantation on quality of life and mental comorbidity in patients aged 80 years. Laryngoscope.

[44] Lassaletta L., Calvino M., Sanchez-Cuadrado I., Skarzynski P.H., Cywka K.B., Czajka N., Kutyba J., Tavora-Vieira D., Van de Heyning P., Mertens G. (2023). QoL, CIs, QALYs, and Individualized Rehabilitation: The Clinical and Practical Benefits of Regularly Assessing the Quality of Life of Adult Cochlear Implant Recipients. Int. J. Environ. Res. Public Health.

[45] Yeni Elbay R., Bakıcı B., Kalcıoğlu M.T. (2023). Depression, Anxiety, and Quality of Life in Patients with Cochlear Implant: A Case-Control Study. J. Int. Adv. Otol..

[46] Bergman P., Lyxell B., Harder H., Mäki-Torkko E. (2020). The outcome of unilateral cochlear implantation in adults: Speech recognition, health-related quality of life and level of anxiety and depression: A one-and three-year follow-up study. Int. Arch. Otorhinolaryngol..

[47] Häußler S.M., Köpke V., Knopke S., Gräbel S., Olze H. (2020). Multifactorial positive influence of cochlear implantation on patients with single-sided deafness. Laryngoscope.

[48] Brüggemann P., Szczepek A.J., Klee K., Gräbel S., Mazurek B., Olze H. (2017). In Patients Undergoing Cochlear Implantation, Psychological Burden Affects Tinnitus and the Overall Outcome of Auditory Rehabilitation. Front. Hum. Neurosci..

[49] Olze H., Szczepek A.J., Haupt H., Förster U., Zirke N., Gräbel S., Mazurek B. (2011). Cochlear implantation has a positive influence on quality of life, tinnitus, and psychological comorbidity. Laryngoscope.

[50] Adamchic I., Tass P.A., Langguth B., Hauptmann C., Koller M., Schecklmann M., Zeman F., Landgrebe M. (2012). Linking the Tinnitus Questionnaire and the subjective Clinical Global Impression: Which differences are clinically important?. Health Qual. Life Outcomes.

[51] Hall D.A., Mehta R.L., Argstatter H. (2018). Interpreting the Tinnitus Questionnaire (German version): What individual differences are clinically important?. Int. J. Audiol..

[52] Olze H., Szczepek A.J., Haupt H., Zirke N., Graebel S., Mazurek B. (2012). The impact of cochlear implantation on tinnitus, stress and quality of life in postlingually deafened patients. Audiol. Neurootol..

[53] Quaranta N., Baguley D., Fanizzi P., Murri A., Pontillo V., Cutler J.M., Cavallaro G. (2023). The effect of cochlear implant and bimodal stimulation on tinnitus: A multinational survey. Acta Otolaryngol..

[54] Rasmussen K.D., West N.C., Bille M., Cayé-Thomasen P. (2023). Tinnitus suppression in a prospective cohort of 45 cochlear implant recipients: Occurrence, degree and correlates. Eur. Arch. Otorhinolaryngol..

[55] Borges A.L.F., Duarte P., Almeida R.B.S., Ledesma A.L.L., Azevedo Y.J., Pereira L.V., Bahmad F. (2021). Cochlear implant and tinnitus-a meta-analysis. Braz. J. Otorhinolaryngol..

[56] Assouly K.K.S., Shabbir M., van Dijk B., Hoare D.J., Akeroyd M.A., Stokroos R.J., Stegeman I., Smit A.L. (2023). The impact of tinnitus on adult cochlear implant recipients: A mixed-method approach. PLoS ONE.

[57] Mosnier I., Ferrary E., Aubry K., Bordure P., Bozorg-Grayeli A., Deguine O., Eyermann C., Franco-Vidal V., Godey B., Guevara N. (2020). The French National Cochlear Implant Registry (EPIIC): Cochlear implantation in adults over 65years old. Eur. Ann. Otorhinolaryngol. Head Neck Dis..

[58] Stöver T., Plontke S.K., Guntinas-Lichius O., Welkoborsky H.J., Zahnert T., Delank K.W., Deitmer T., Esser D., Dietz A., Wienke A. (2023). Structure and establishment of the German Cochlear Implant Registry (DCIR). HNO.

[59] Berrettini S., Arslan E., Baggiani A., Burdo S., Cassandro E., Cuda D., Dinelli E., Filipo R., Mancini P., Martini A. (2011). A registry for the collection of data in cochlear implant patients. Acta Otorhinolaryngol. Ital..

[60] Loth A., Vazzana C., Leinung M., Guderian D., Issing C., Baumann U., Stöver T. (2022). Quality control in cochlear implant therapy: Clinical practice guidelines and registries in European countries. Eur. Arch. Otorhinolaryngol..

[61] Lenarz T., Büchner A., Illg A. (2022). Cochlear Implantation: Concept, Results Outcomes and Quality of Life. Laryngorhinootologie.

[62] McRackan T.R., Hand B.N., Velozo C.A., Dubno J.R. (2019). Association of Demographic and Hearing-Related Factors with Cochlear Implant-Related Quality of Life. JAMA Otolaryngol. Head Neck Surg..

[63] Lee E., Pisa J., Hochman J. (2023). Comorbidity associated with worse outcomes in a population of limited cochlear implant performers. Laryngoscope Investig. Otolaryngol..

