# Supplementary material for: Changes in Speech Intelligibility, Health-Related Quality of Life, Depressive Symptoms, Anxiety, Perceived Stress, and Tinnitus-Induced Distress, in a Cohort of 227 Adults One Year After Cochlear Implantation: A Decade of Experience from a Single Tertiary Center
Source: J Clin Med. 2025 Nov 17;14(22):8143. doi: 10.3390/jcm14228143 (PMC12653197; doi:10.3390/jcm14228143)
Supplement: Supplementary file 1 [file jcm-14-08143-s001.zip › S1.pdf]

S1: Kruskal-Wallis Test: the comparison between the pre- and post-scores of NCIQ for AHL, DSD, and SSD groups.

Hypothesis Test Summary

	Null Hypothesis	Test	Sig.[a,b]	Decision
1	The distribution of Rank of @1_NCIQtotal is the same across categories of type of hearing loss.	Independent-Samples Kruskal-Wallis Test	,014	Reject the null hypothesis.
2	The distribution of NCIQ1 is the same across categories of type of hearing loss.	Independent-Samples Kruskal-Wallis Test	<,001	Reject the null hypothesis.
3	The distribution of NCIQ2 is the same across categories of type of hearing loss.	Independent-Samples Kruskal-Wallis Test	<,001	Reject the null hypothesis.
4	The distribution of NCIQ3 is the same across categories of type of hearing loss.	Independent-Samples Kruskal-Wallis Test	<,001	Reject the null hypothesis.
5	The distribution of NCIQ4 is the same across categories of type of hearing loss.	Independent-Samples Kruskal-Wallis Test	,015	Reject the null hypothesis.
6	The distribution of NCIQ5 is the same across categories of type of hearing loss.	Independent-Samples Kruskal-Wallis Test	,006	Reject the null hypothesis.
7	The distribution of NCIQ6 is the same across categories of type of hearing loss.	Independent-Samples Kruskal-Wallis Test	,011	Reject the null hypothesis.
8	The distribution of NCIQtotal is the same across categories of type of hearing loss.	Independent-Samples Kruskal-Wallis Test	<,001	Reject the null hypothesis.
9	The distribution of 1_NCIQ1 is the same across categories of type of hearing loss.	Independent-Samples Kruskal-Wallis Test	,012	Reject the null hypothesis.

10	The distribution of 1_NCIQ2 is the same across categories of type of hearing loss.	Independent-Samples Kruskal-Wallis Test	<,001	Reject the null hypothesis.
11	The distribution of 1_NCIQ3 is the same across categories of type of hearing loss.	Independent-Samples Kruskal-Wallis Test	,004	Reject the null hypothesis.
12	The distribution of 1_NCIQ4 is the same across categories of type of hearing loss.	Independent-Samples Kruskal-Wallis Test	,695	Retain the null hypothesis.
13	The distribution of 1_NCIQ5 is the same across categories of type of hearing loss.	Independent-Samples Kruskal-Wallis Test	,197	Retain the null hypothesis.
14	The distribution of 1_NCIQ6 is the same across categories of type of hearing loss.	Independent-Samples Kruskal-Wallis Test	,053	Retain the null hypothesis.
15	The distribution of 1_NCIQtotal is the same across categories of type of hearing loss.	Independent-Samples Kruskal-Wallis Test	,014	Reject the null hypothesis.

a. The significance level is ,050.

b. Asymptotic significance is displayed.

Independent-Samples Kruskal-Wallis Test

Rank of @1_NCIQtotal across type of hearing loss

Independent-Samples Kruskal-Wallis Test Summary

Total N	220
Test Statistic	8,603[a]
Degree Of Freedom	2

Asymptotic Sig.(2-sided test)	,014

a. The test statistic is adjusted for ties.

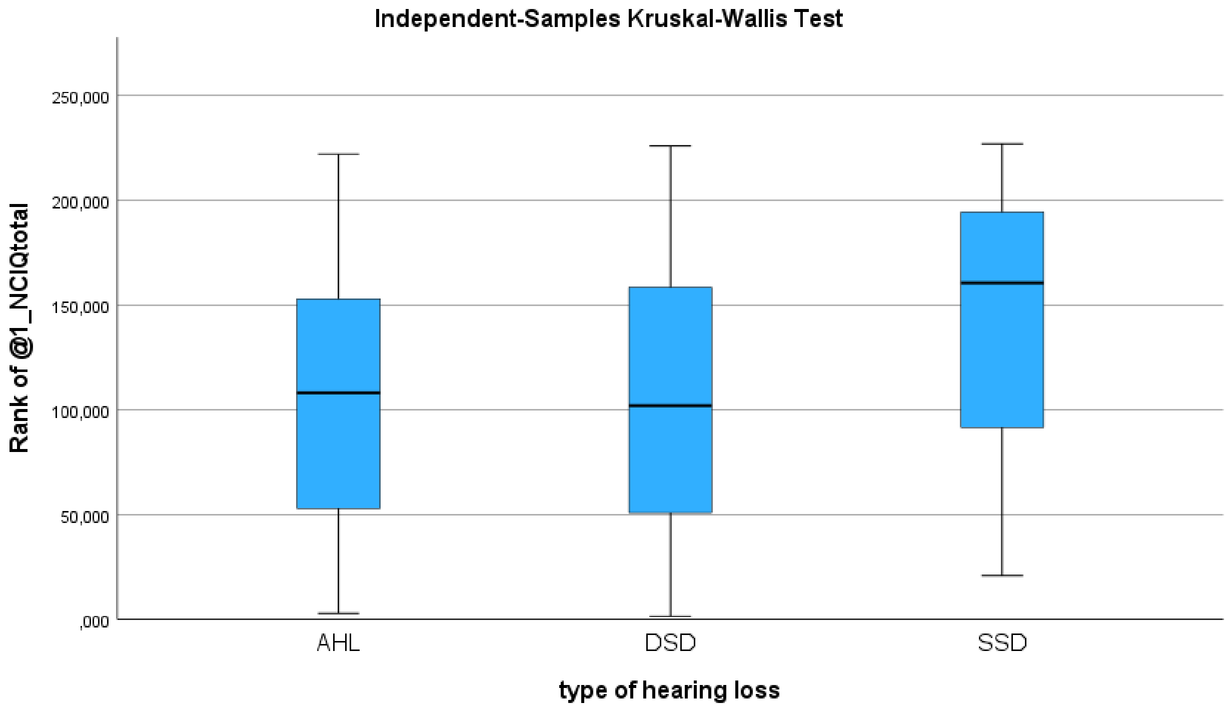

Pairwise Comparisons of type of hearing loss

Sample 1-Sample 2	Test Statistic	Std. Error	Std. Test Statistic	Sig.	Adj. Sig.[a]
AHL-DSD	-2,249	10,193	-,221	,825	1,000
AHL-SSD	-33,163	12,809	-2,589	,010	,029
DSD-SSD	-30,915	11,326	-2,730	,006	,019

Each row tests the null hypothesis that the Sample 1 and Sample 2 distributions are the same.
Asymptotic significances (2-sided tests) are displayed. The significance level is ,050.
a. Significance values have been adjusted by the Bonferroni correction for multiple tests.

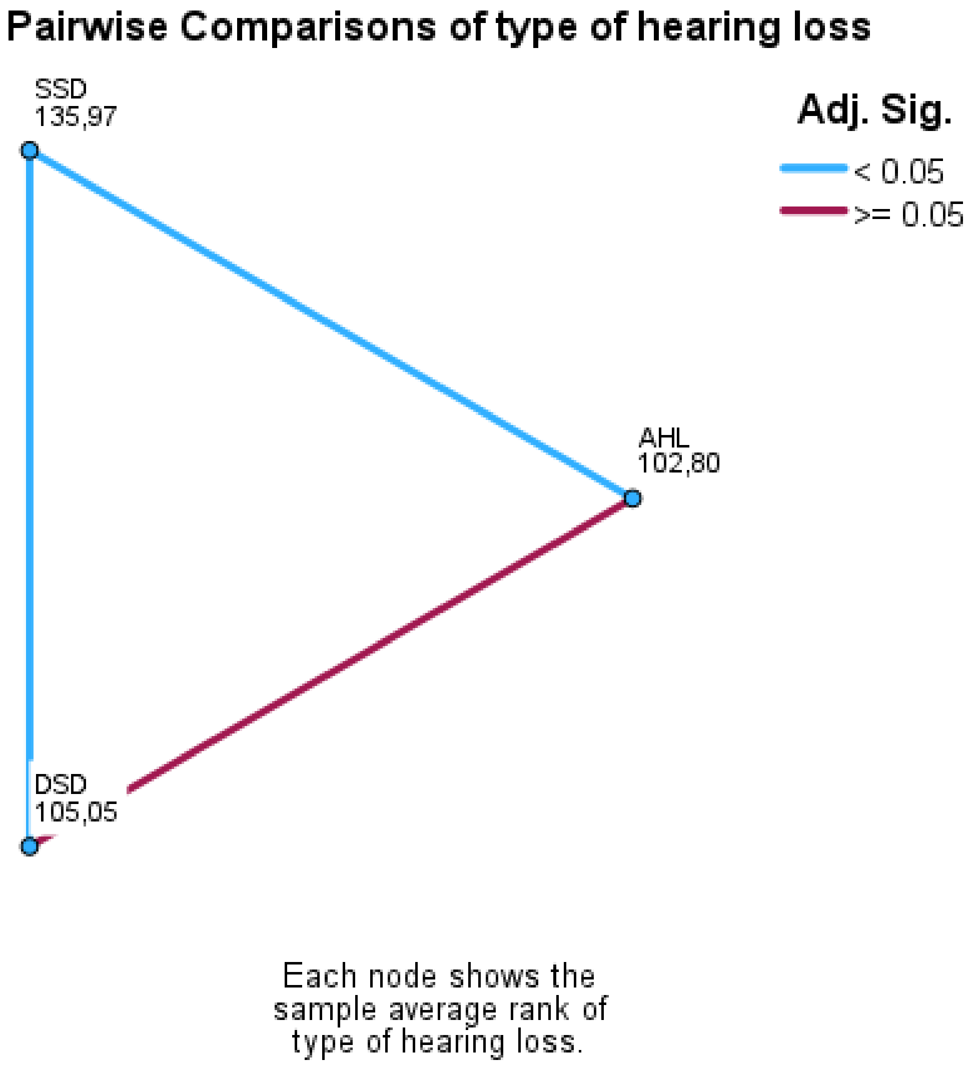

NCIQ1 across type of hearing loss

Independent-Samples Kruskal-Wallis Test Summary

Total N	220
Test Statistic	42,411[a]
Degree Of Freedom	2
Asymptotic Sig.(2-sided test)	<,001

a. The test statistic is adjusted for ties.

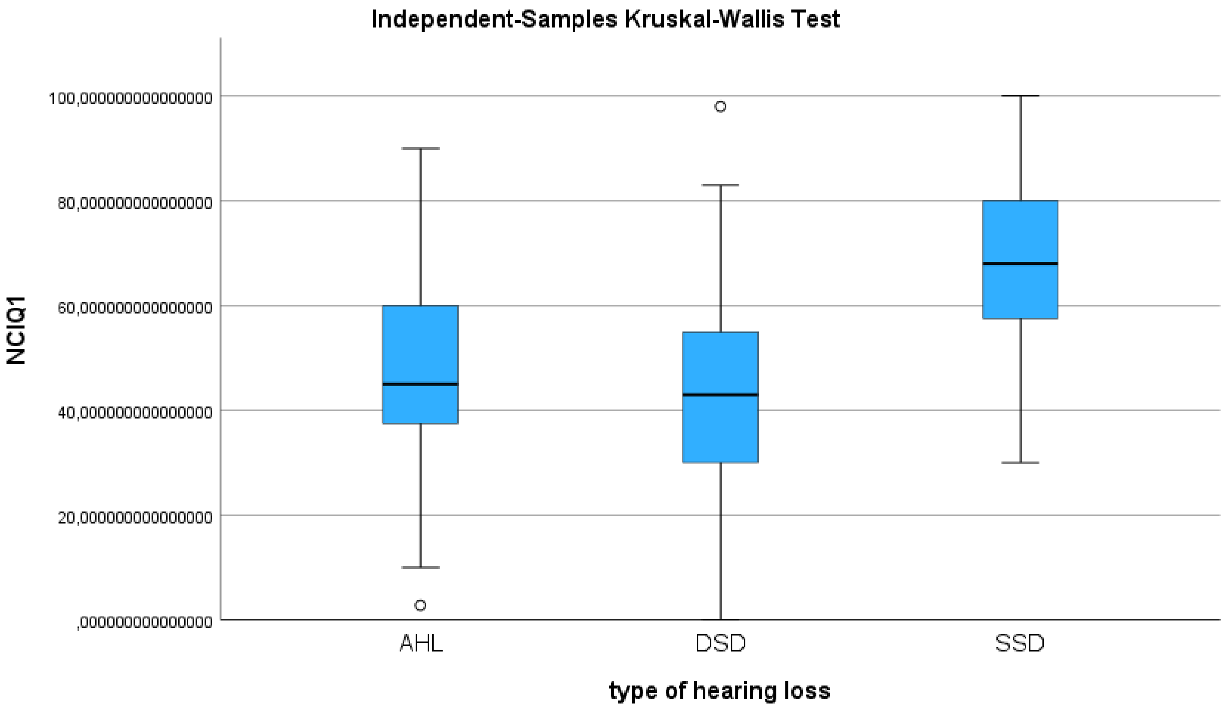

Pairwise Comparisons of type of hearing loss

Sample 1-Sample 2	Test Statistic	Std. Error	Std. Test Statistic	Sig.	Adj. Sig.[a]
DSD-AHL	17,405	10,190	1,708	,088	,263
DSD-SSD	-73,689	11,322	-6,509	<,001	,000
AHL-SSD	-56,284	12,805	-4,395	<,001	,000

Each row tests the null hypothesis that the Sample 1 and Sample 2 distributions are the same.

Asymptotic significances (2-sided tests) are displayed. The significance level is ,050.

a. Significance values have been adjusted by the Bonferroni correction for multiple tests.

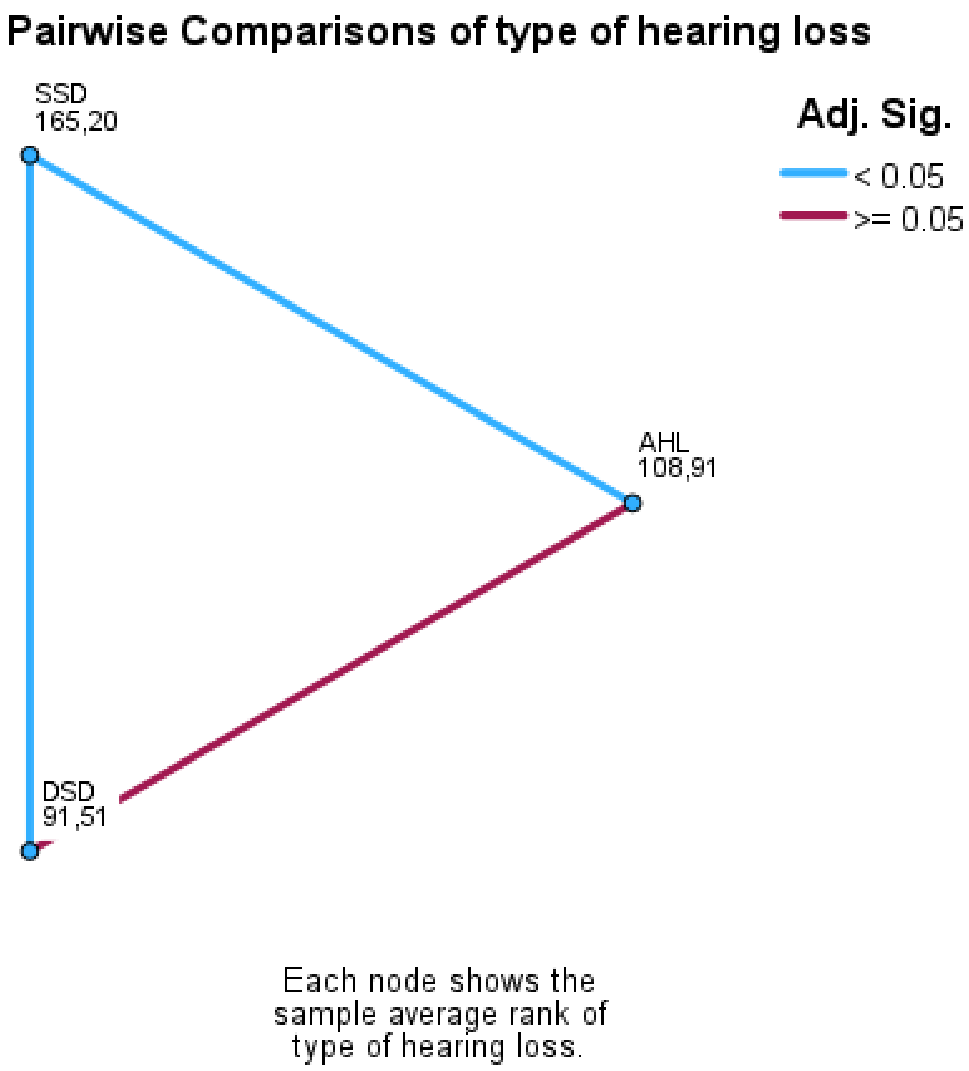

NCIQ2 across type of hearing loss

Independent-Samples Kruskal-Wallis Test Summary

Total N	220
Test Statistic	56,059[a]
Degree Of Freedom	2
Asymptotic Sig.(2-sided test)	<,001

a. The test statistic is adjusted for ties.

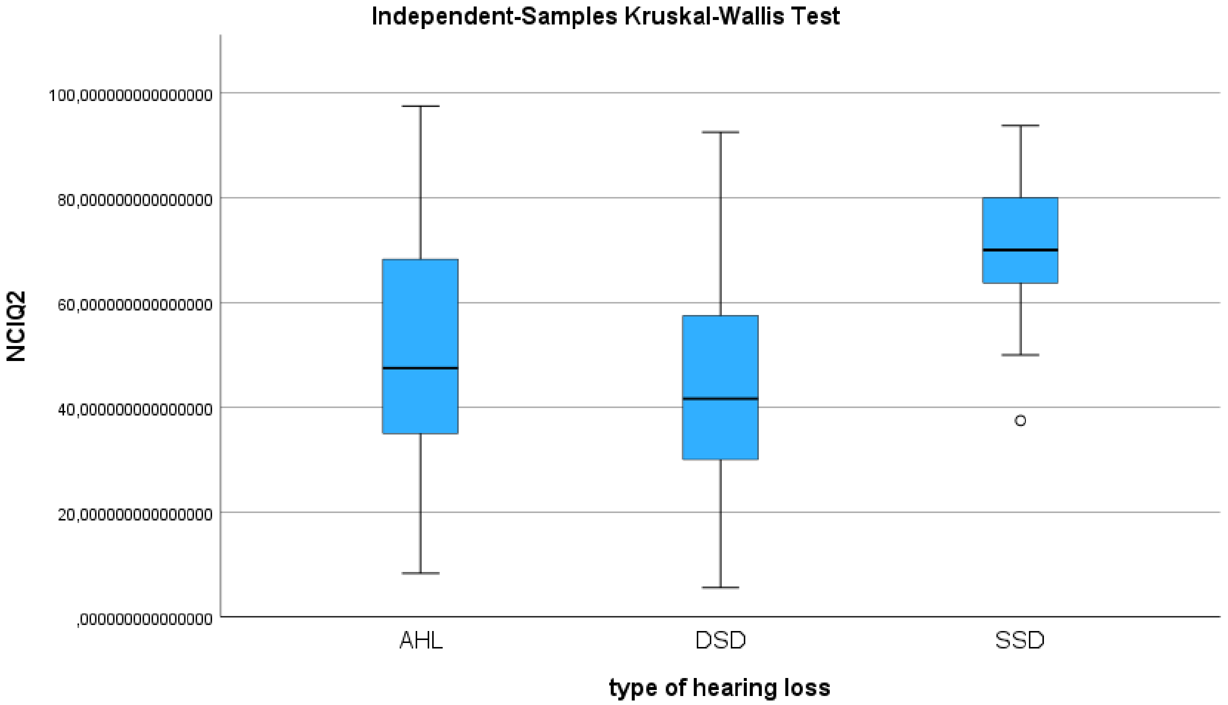

Pairwise Comparisons of type of hearing loss

Sample 1-Sample 2	Test Statistic	Std. Error	Std. Test Statistic	Sig.	Adj. Sig.[a]
DSD-AHL	22,158	10,188	2,175	,030	,089
DSD-SSD	-84,756	11,320	-7,487	<,001	,000
AHL-SSD	-62,599	12,803	-4,889	<,001	,000

Each row tests the null hypothesis that the Sample 1 and Sample 2 distributions are the same.

Asymptotic significances (2-sided tests) are displayed. The significance level is ,050.

a. Significance values have been adjusted by the Bonferroni correction for multiple tests.

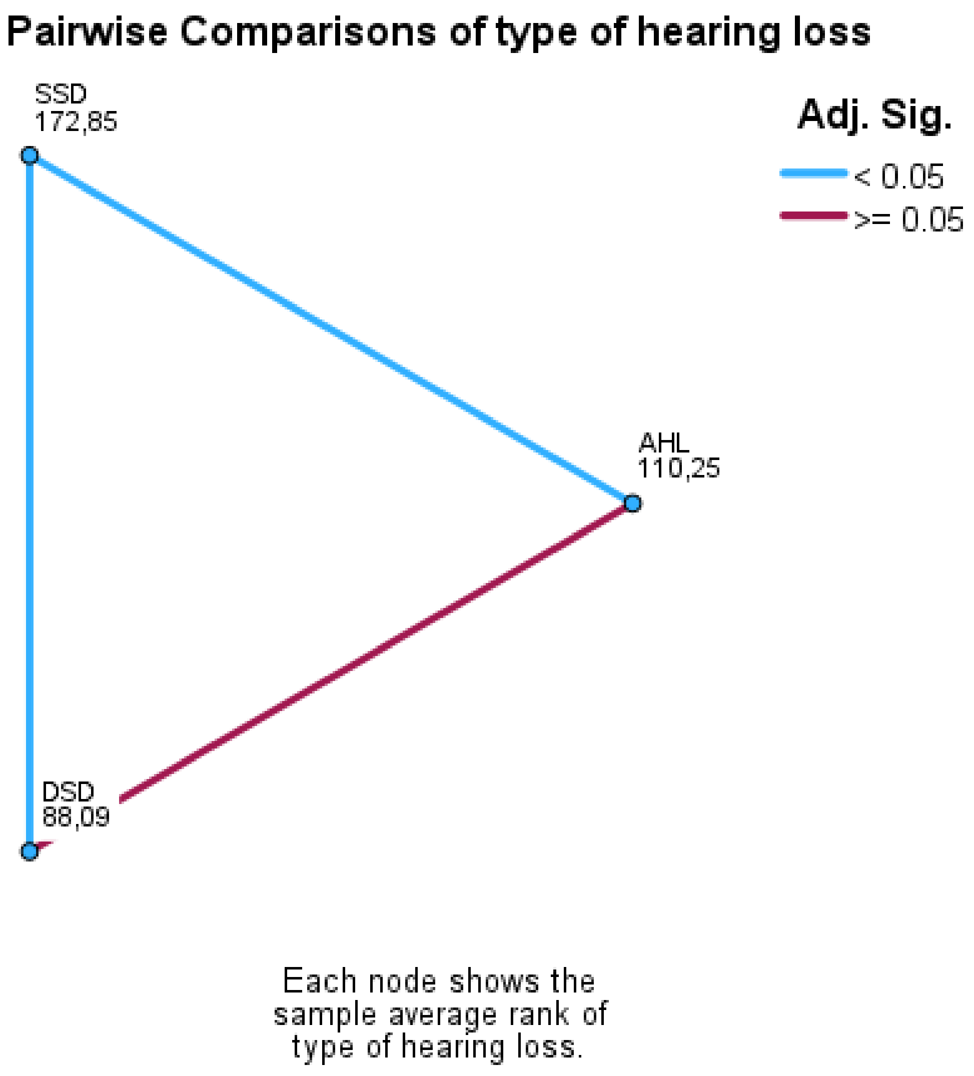

NCIQ3 across type of hearing loss

Independent-Samples Kruskal-Wallis Test Summary

Total N	220
Test Statistic	18,665[a]
Degree Of Freedom	2
Asymptotic Sig.(2-sided test)	<,001

a. The test statistic is adjusted for ties.

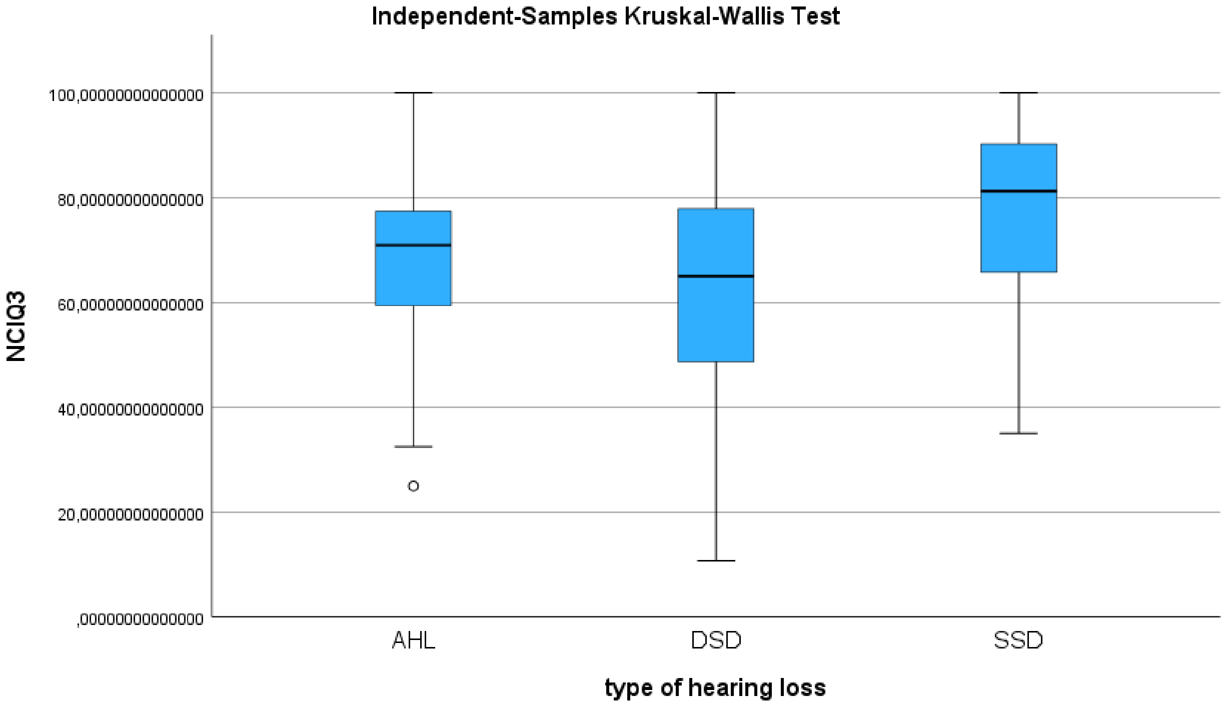

Pairwise Comparisons of type of hearing loss

Sample 1-Sample 2	Test Statistic	Std. Error	Std. Test Statistic	Sig.	Adj. Sig.[a]
DSD-AHL	17,947	10,190	1,761	,078	,235
DSD-SSD	-48,560	11,322	-4,289	<,001	,000
AHL-SSD	-30,613	12,805	-2,391	,017	,050

Each row tests the null hypothesis that the Sample 1 and Sample 2 distributions are the same.
Asymptotic significances (2-sided tests) are displayed. The significance level is ,050.
a. Significance values have been adjusted by the Bonferroni correction for multiple tests.

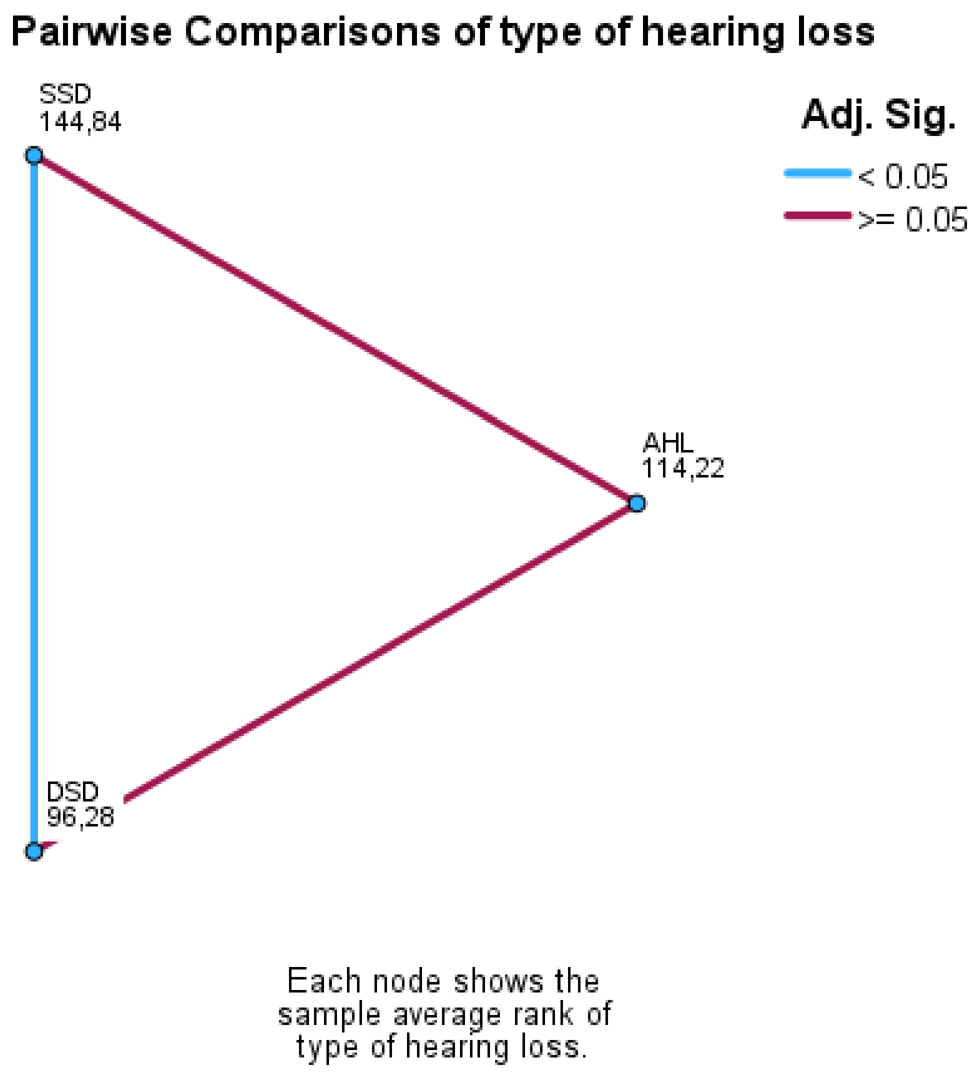

NCIQ4 across type of hearing loss

Independent-Samples Kruskal-Wallis Test Summary

Total N	220
Test Statistic	8,453[a]
Degree Of Freedom	2
Asymptotic Sig.(2-sided test)	,015

a. The test statistic is adjusted for ties.

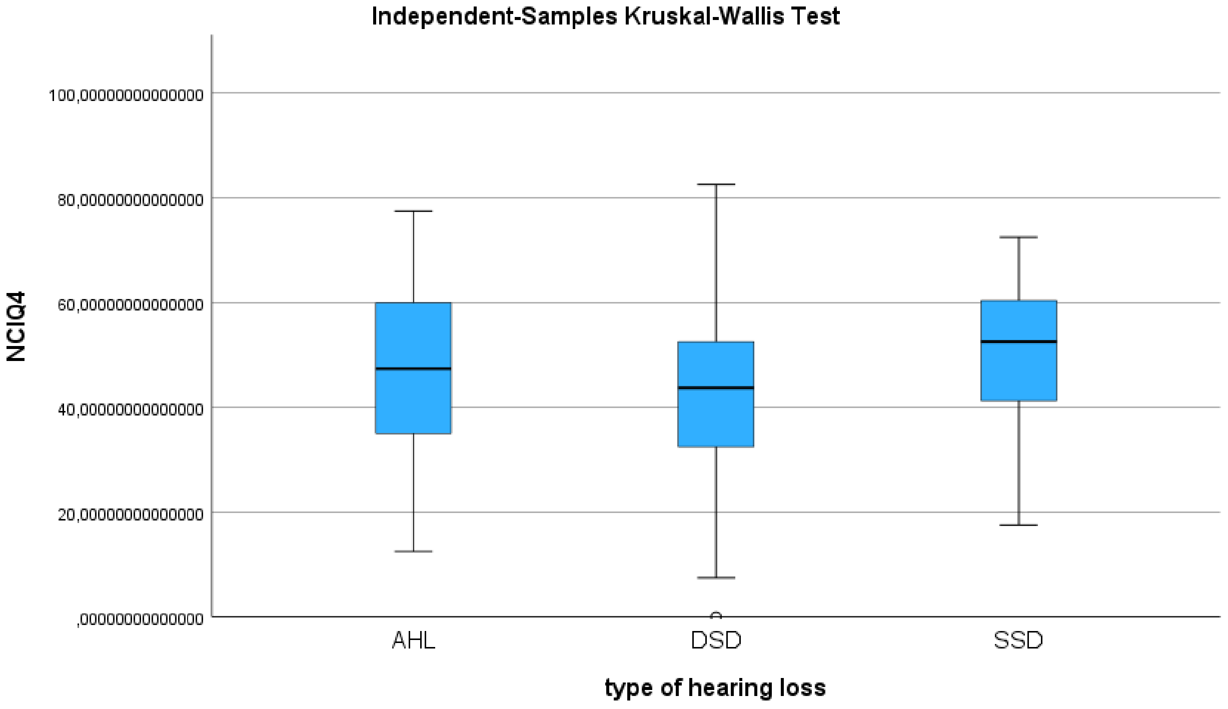

Pairwise Comparisons of type of hearing loss

Sample 1-Sample 2	Test Statistic	Std. Error	Std. Test Statistic	Sig.	Adj. Sig.[a]
DSD-AHL	15,127	10,185	1,485	,137	,412
DSD-SSD	-31,984	11,316	-2,826	,005	,014
AHL-SSD	-16,856	12,799	-1,317	,188	,564

Each row tests the null hypothesis that the Sample 1 and Sample 2 distributions are the same.

Asymptotic significances (2-sided tests) are displayed. The significance level is ,050.

a. Significance values have been adjusted by the Bonferroni correction for multiple tests.

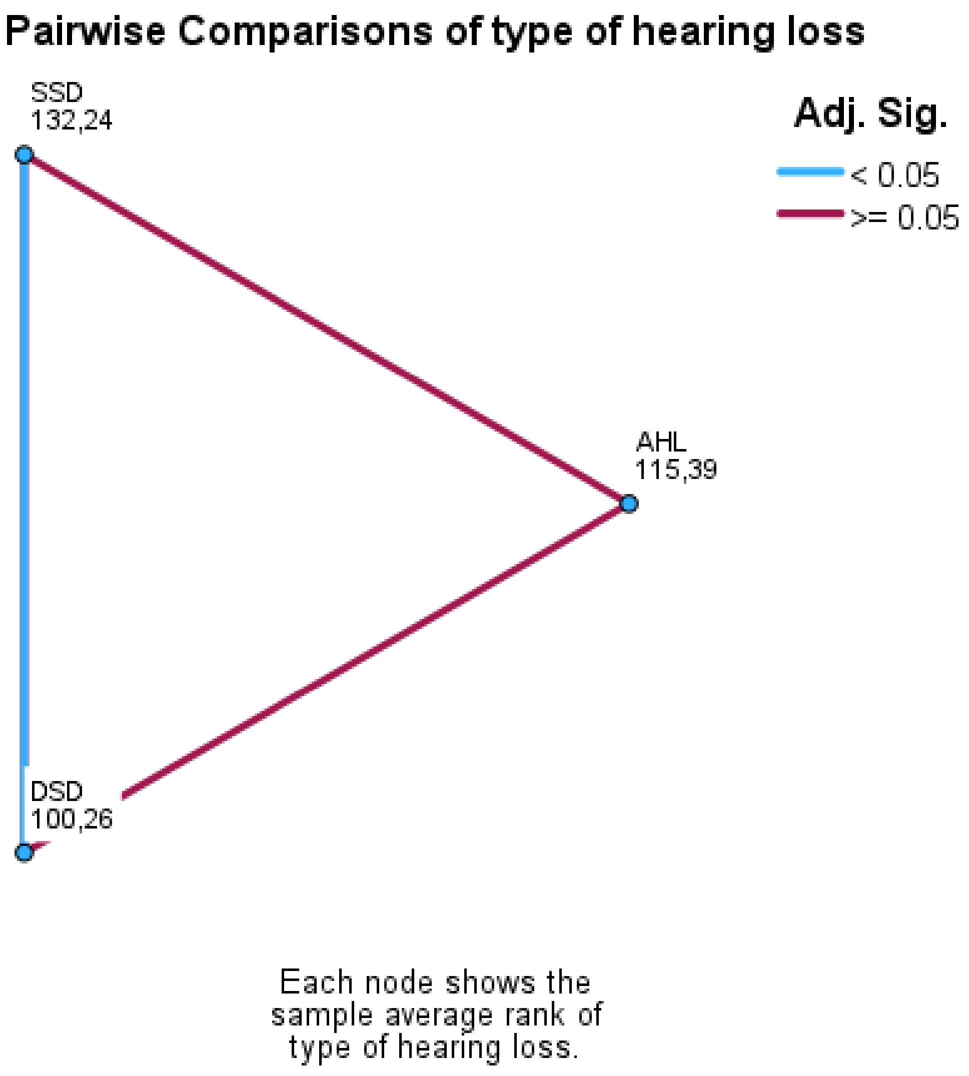

NCIQ5 across type of hearing loss

Independent-Samples Kruskal-Wallis Test Summary

Total N	220
Test Statistic	10,396[a]
Degree Of Freedom	2
Asymptotic Sig.(2-sided test)	,006

a. The test statistic is adjusted for ties.

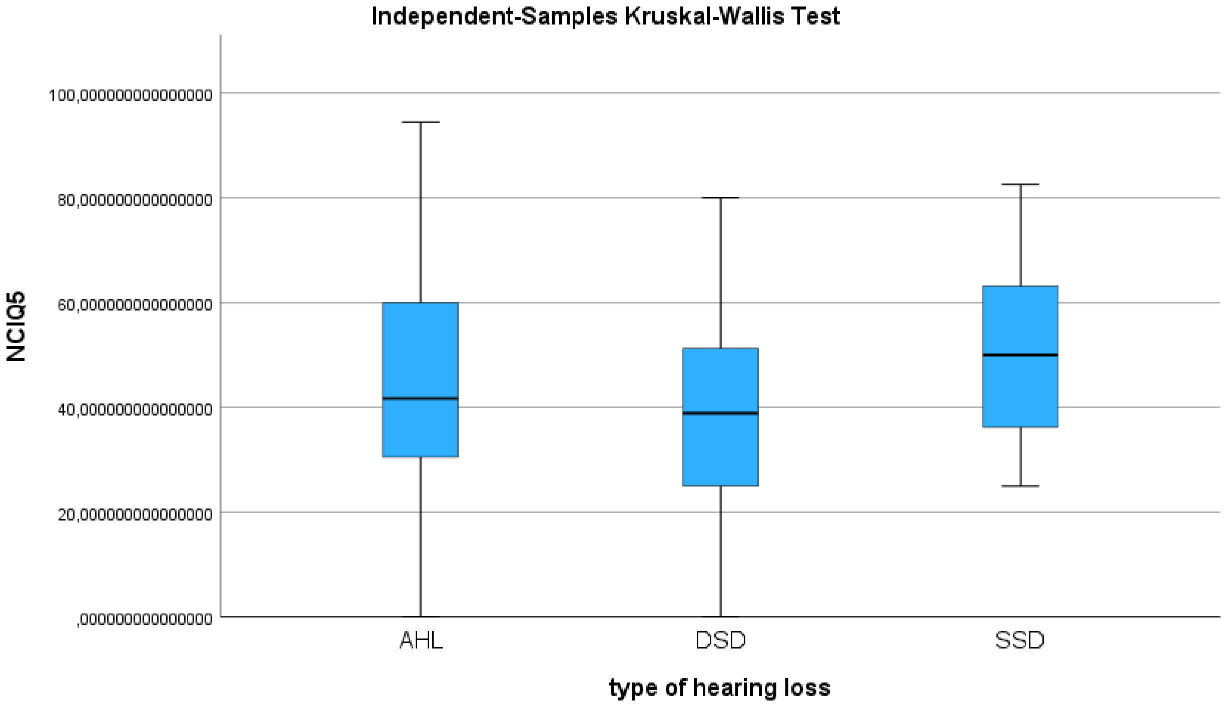

Pairwise Comparisons of type of hearing loss

Sample 1-Sample 2	Test Statistic	Std. Error	Std. Test Statistic	Sig.	Adj. Sig.[a]
DSD-AHL	17,097	10,190	1,678	,093	,280
DSD-SSD	-35,389	11,322	-3,126	,002	,005
AHL-SSD	-18,293	12,805	-1,429	,153	,459

Each row tests the null hypothesis that the Sample 1 and Sample 2 distributions are the same.

Asymptotic significances (2-sided tests) are displayed. The significance level is ,050.

a. Significance values have been adjusted by the Bonferroni correction for multiple tests.

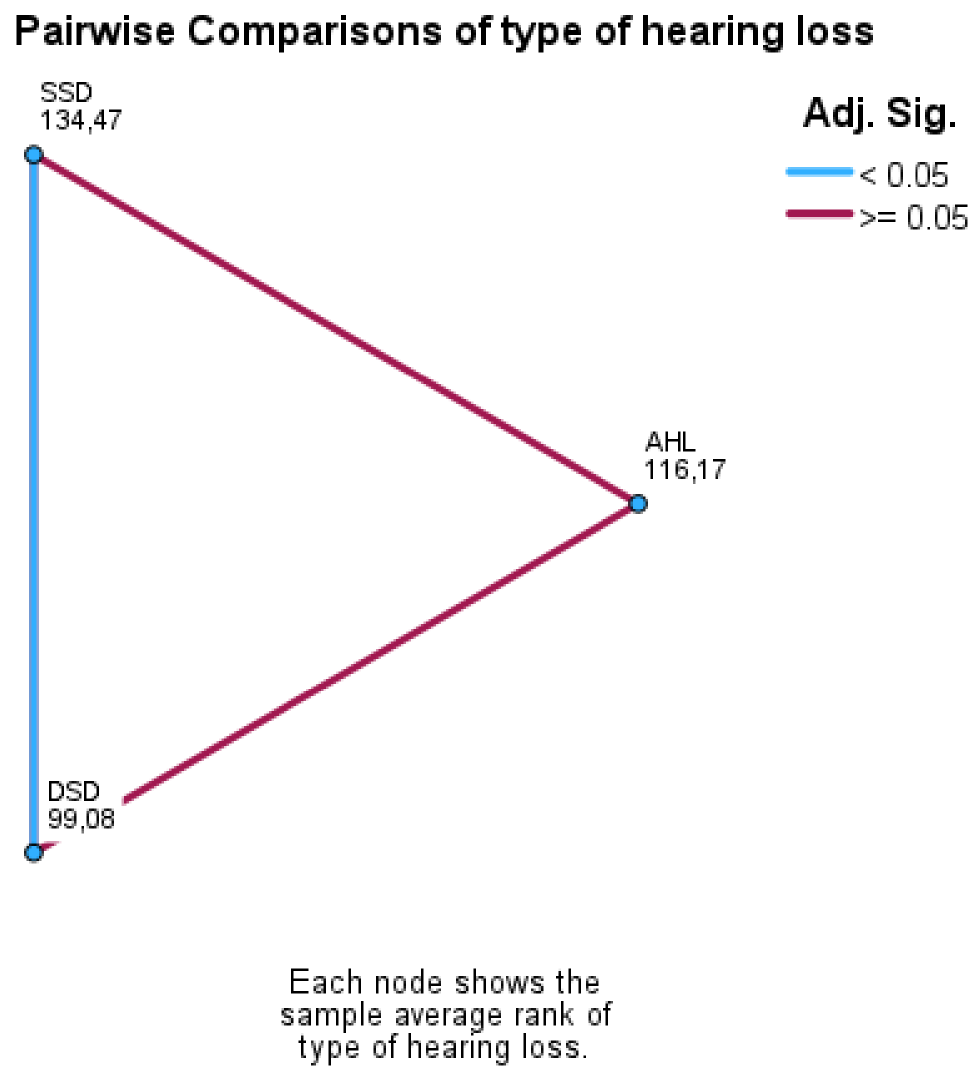

NCIQ6 across type of hearing loss

Independent-Samples Kruskal-Wallis Test Summary

Total N	220
Test Statistic	9,039[a]
Degree Of Freedom	2
Asymptotic Sig.(2-sided test)	,011

a. The test statistic is adjusted for ties.

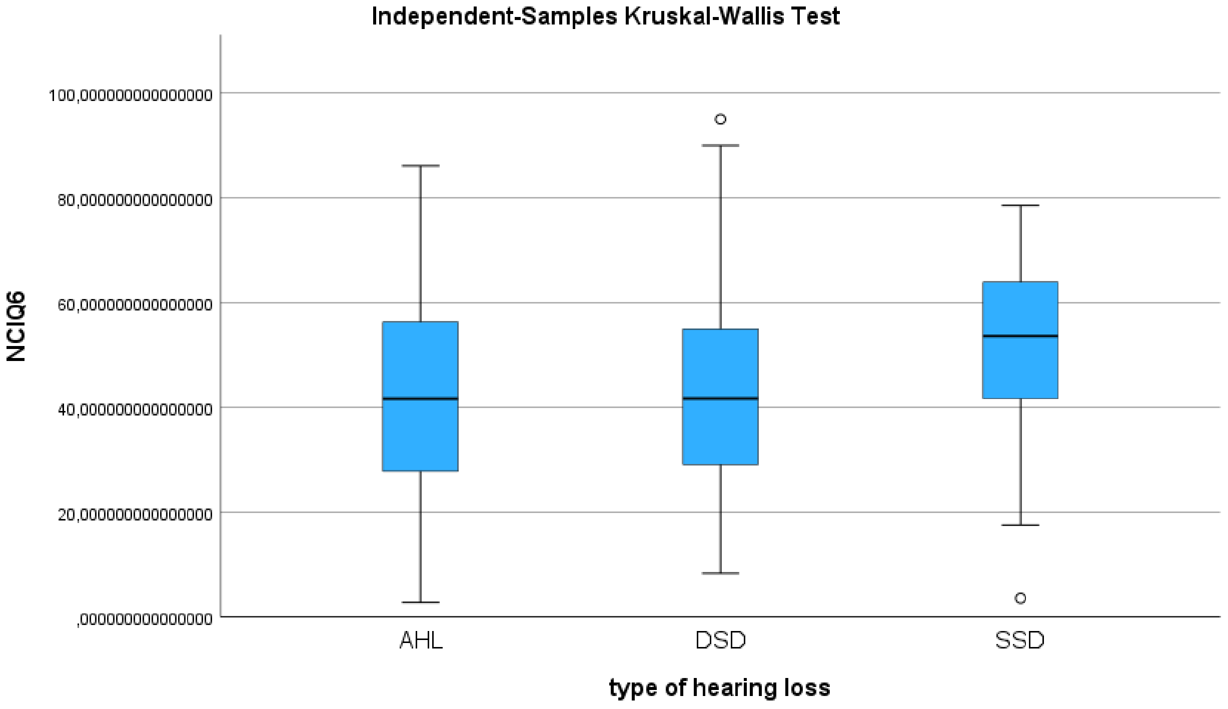

Pairwise Comparisons of type of hearing loss

Sample 1-Sample 2	Test Statistic	Std. Error	Std. Test Statistic	Sig.	Adj. Sig.[a]
DSD-AHL	1,064	10,190	,104	,917	1,000
DSD-SSD	-32,853	11,322	-2,902	,004	,011
AHL-SSD	-31,789	12,805	-2,483	,013	,039

Each row tests the null hypothesis that the Sample 1 and Sample 2 distributions are the same.

Asymptotic significances (2-sided tests) are displayed. The significance level is ,050.

a. Significance values have been adjusted by the Bonferroni correction for multiple tests.

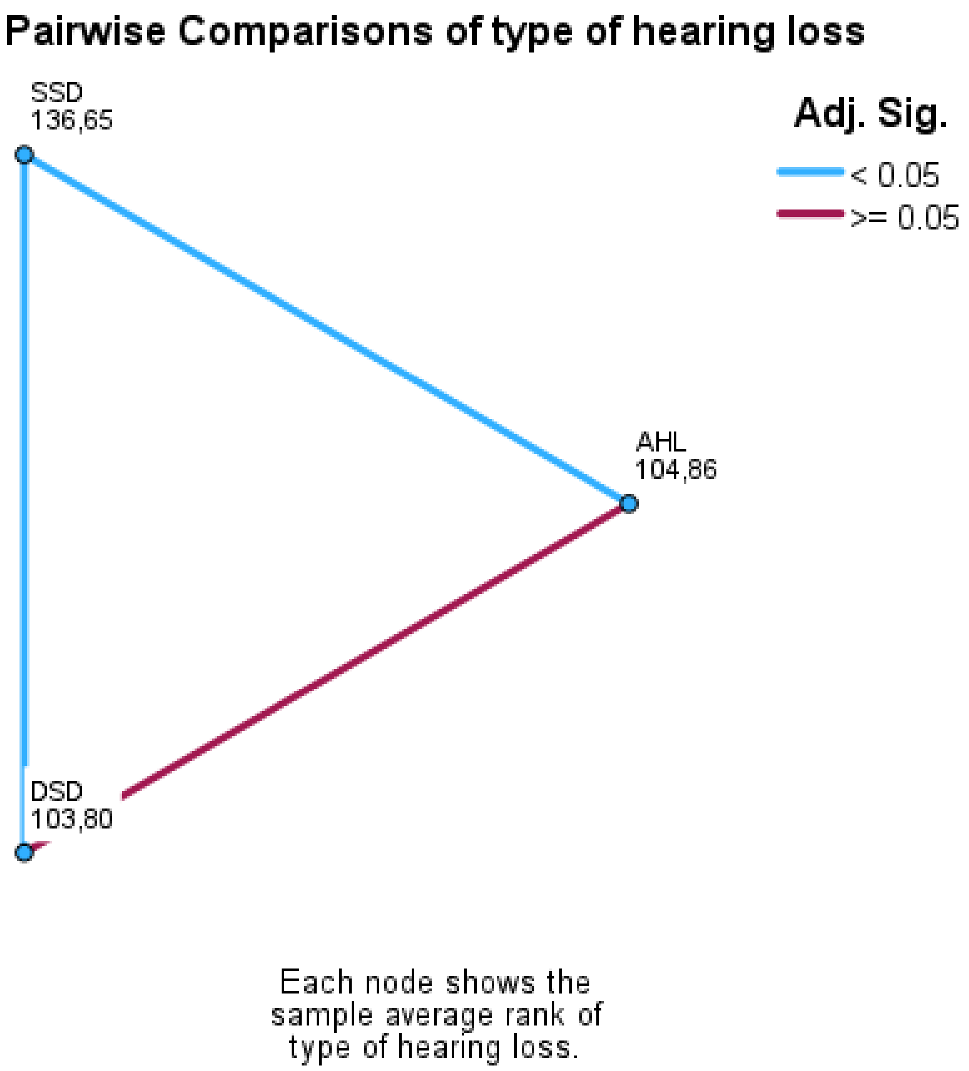

NCIQtotal across type of hearing loss

Independent-Samples Kruskal-Wallis Test Summary

Total N	220
Test Statistic	37,450[a]
Degree Of Freedom	2
Asymptotic Sig.(2-sided test)	<,001

a. The test statistic is adjusted for ties.

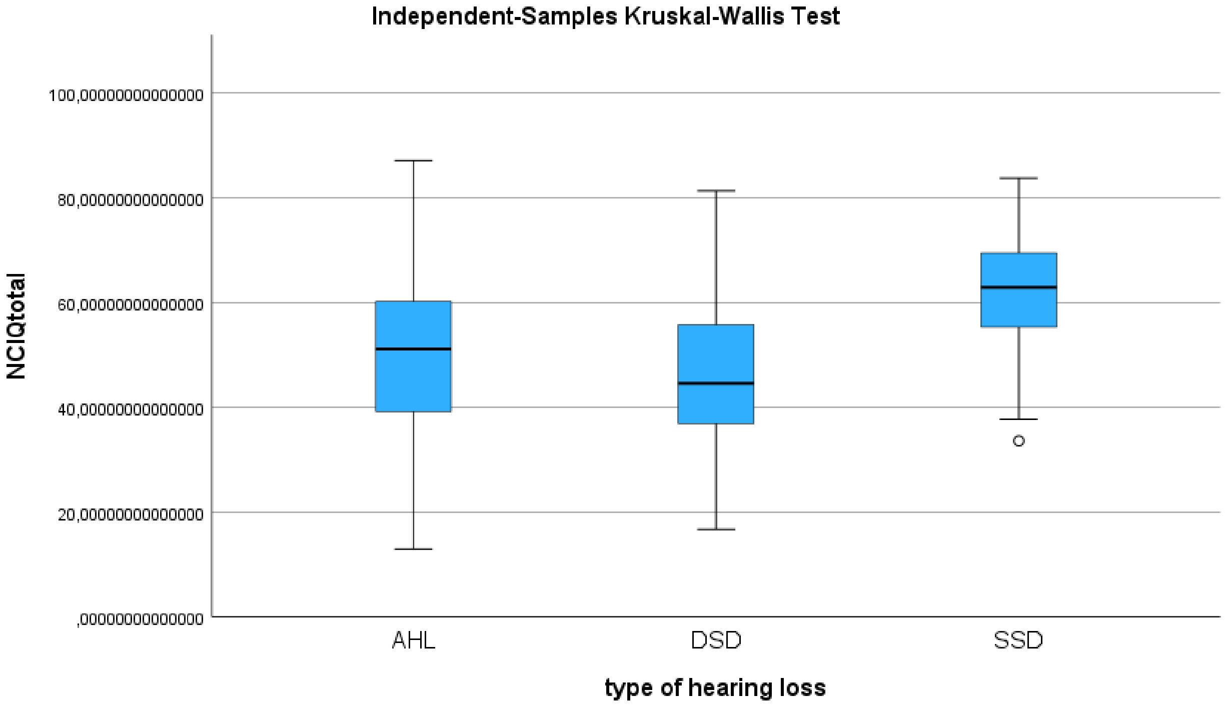

Pairwise Comparisons of type of hearing loss

Sample 1-Sample 2	Test Statistic	Std. Error	Std. Test Statistic	Sig.	Adj. Sig.[a]
DSD-AHL	18,151	10,193	1,781	,075	,225
DSD-SSD	-69,309	11,326	-6,120	<,001	,000
AHL-SSD	-51,158	12,809	-3,994	<,001	,000

Each row tests the null hypothesis that the Sample 1 and Sample 2 distributions are the same.
Asymptotic significances (2-sided tests) are displayed. The significance level is ,050.
a. Significance values have been adjusted by the Bonferroni correction for multiple tests.

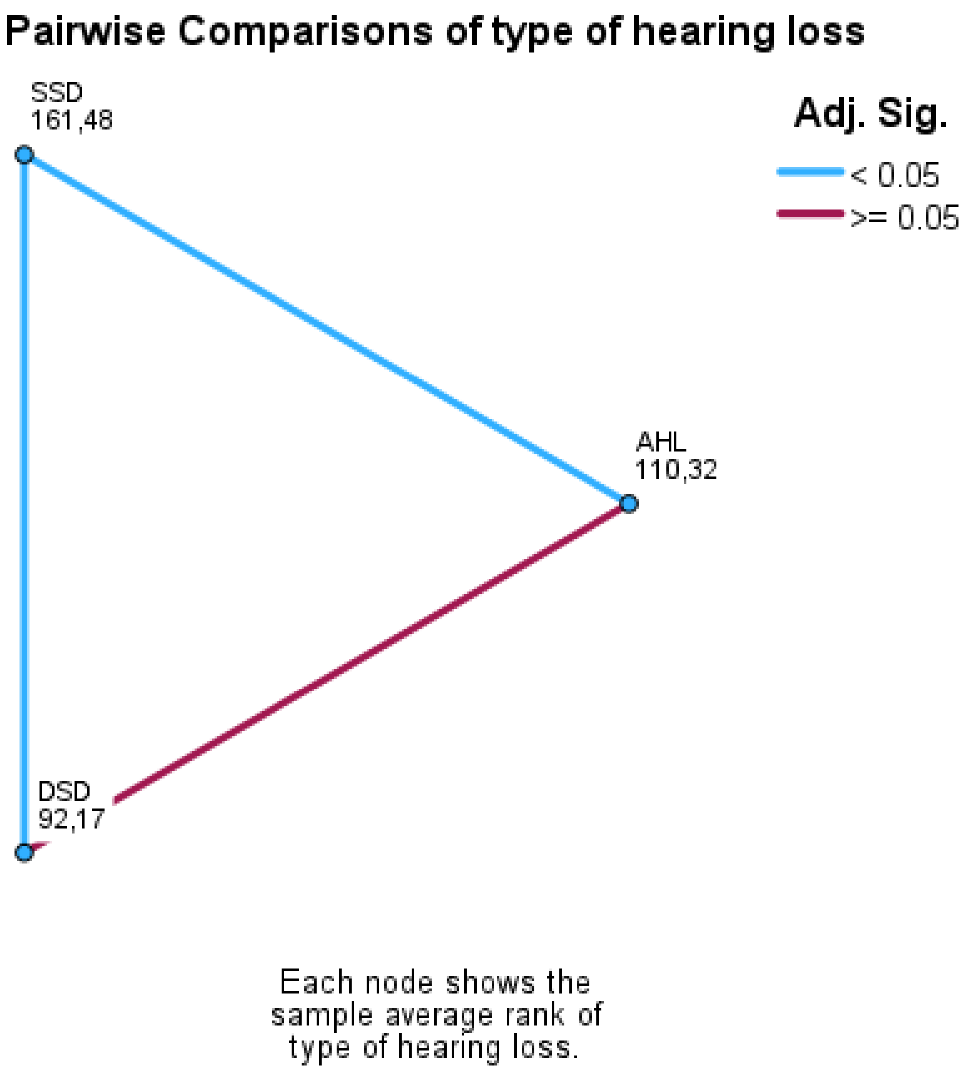

1_NCIQ1 across type of hearing loss

Independent-Samples Kruskal-Wallis Test Summary

Total N	219
Test Statistic	8,799[a]
Degree Of Freedom	2
Asymptotic Sig.(2-sided test)	,012

a. The test statistic is adjusted for ties.

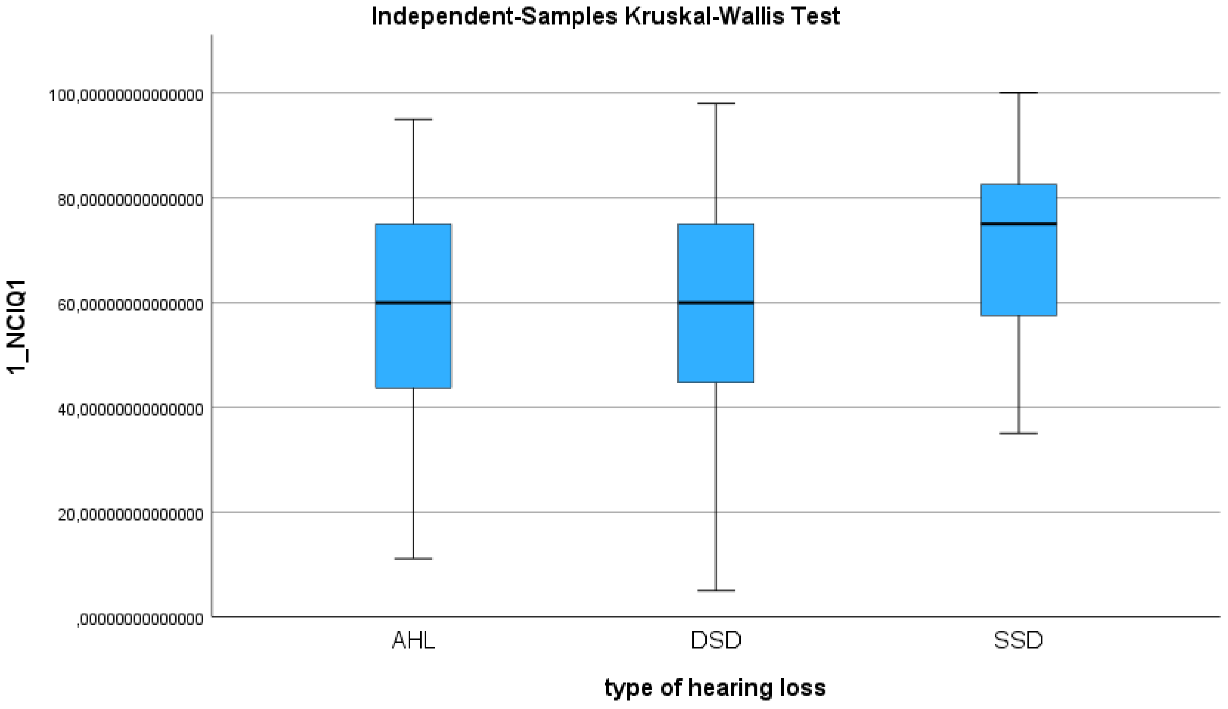

Pairwise Comparisons of type of hearing loss

Sample 1-Sample 2	Test Statistic	Std. Error	Std. Test Statistic	Sig.	Adj. Sig.[a]
AHL-DSD	-2,040	10,203	-,200	,842	1,000
AHL-SSD	-33,268	12,794	-2,600	,009	,028
DSD-SSD	-31,229	11,270	-2,771	,006	,017

Each row tests the null hypothesis that the Sample 1 and Sample 2 distributions are the same.
Asymptotic significances (2-sided tests) are displayed. The significance level is ,050.
a. Significance values have been adjusted by the Bonferroni correction for multiple tests.

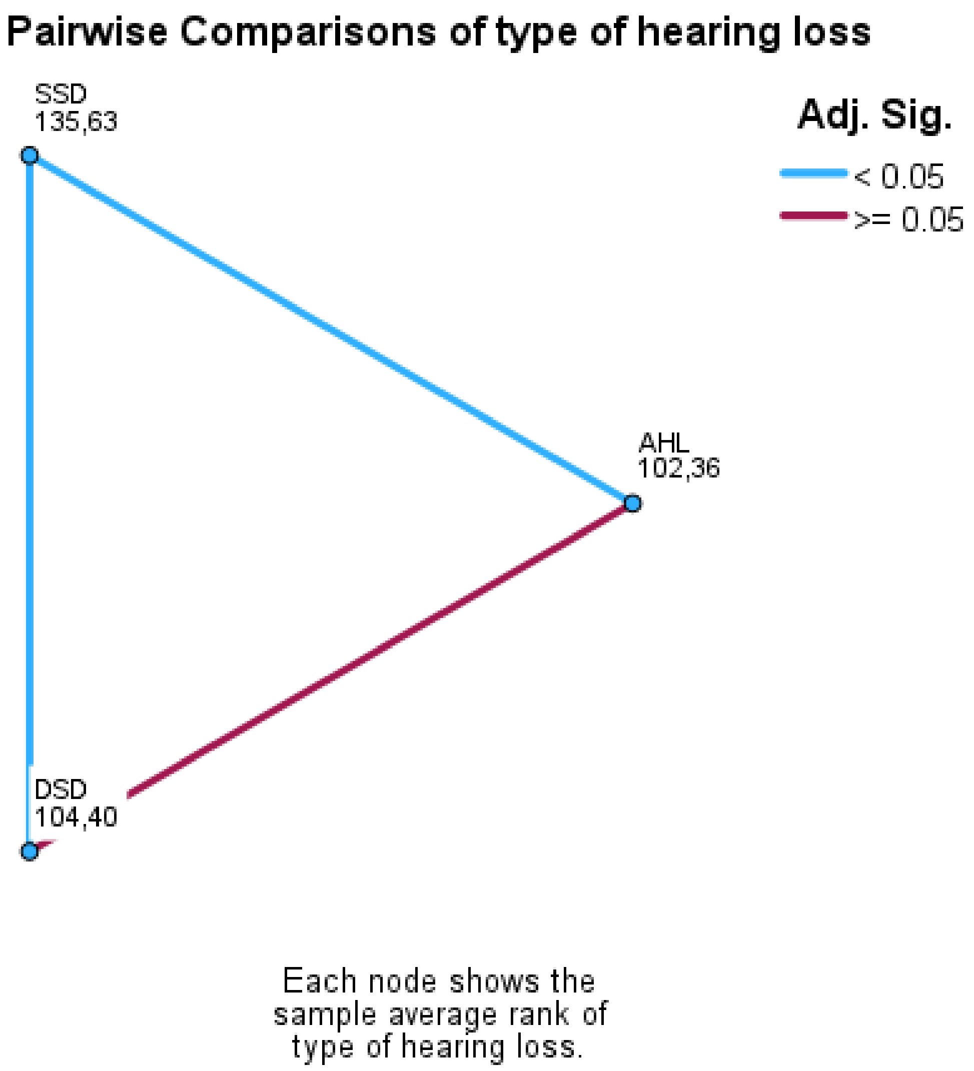

1_NCIQ2 across type of hearing loss

Independent-Samples Kruskal-Wallis Test Summary

Total N	220
Test Statistic	24,343[a]
Degree Of Freedom	2
Asymptotic Sig.(2-sided test)	<,001

a. The test statistic is adjusted for ties.

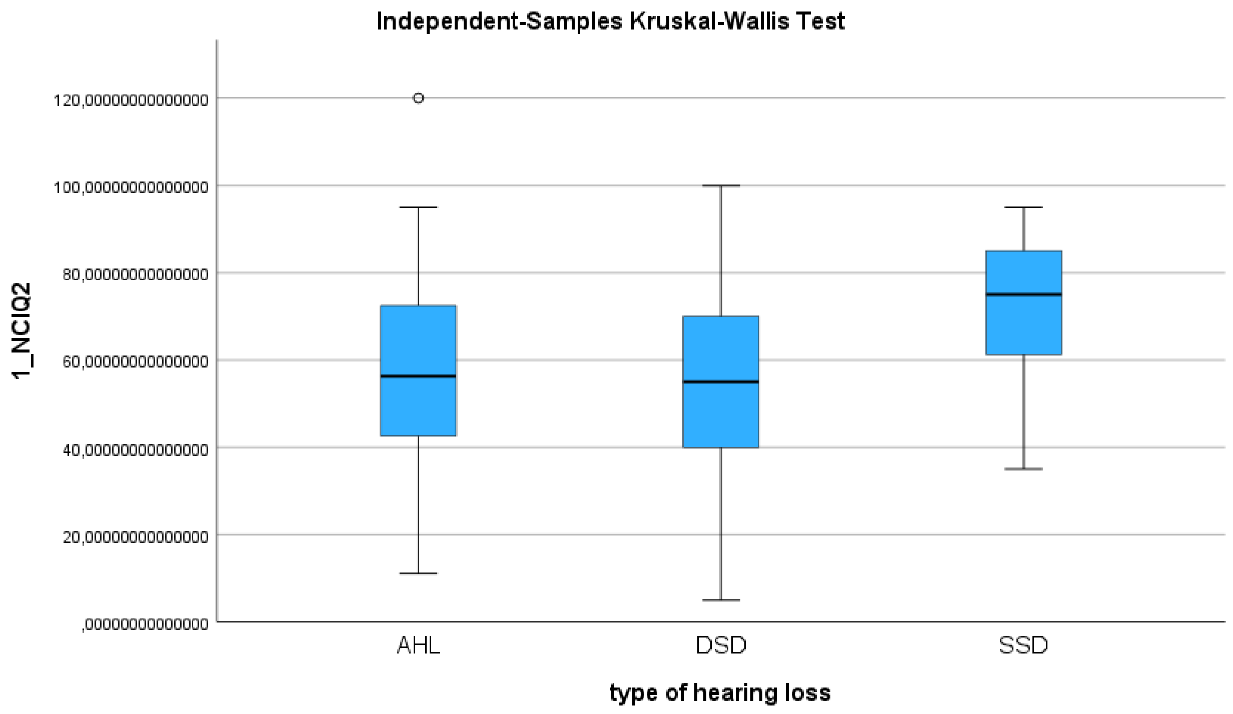

Pairwise Comparisons of type of hearing loss

Sample 1-Sample 2	Test Statistic	Std. Error	Std. Test Statistic	Sig.	Adj. Sig.[a]
DSD-AHL	7,874	10,188	,773	,440	1,000
DSD-SSD	-55,289	11,320	-4,884	<,001	,000
AHL-SSD	-47,416	12,803	-3,703	<,001	,001

Each row tests the null hypothesis that the Sample 1 and Sample 2 distributions are the same.
Asymptotic significances (2-sided tests) are displayed. The significance level is ,050.
a. Significance values have been adjusted by the Bonferroni correction for multiple tests.

Pairwise Comparisons of type of hearing loss

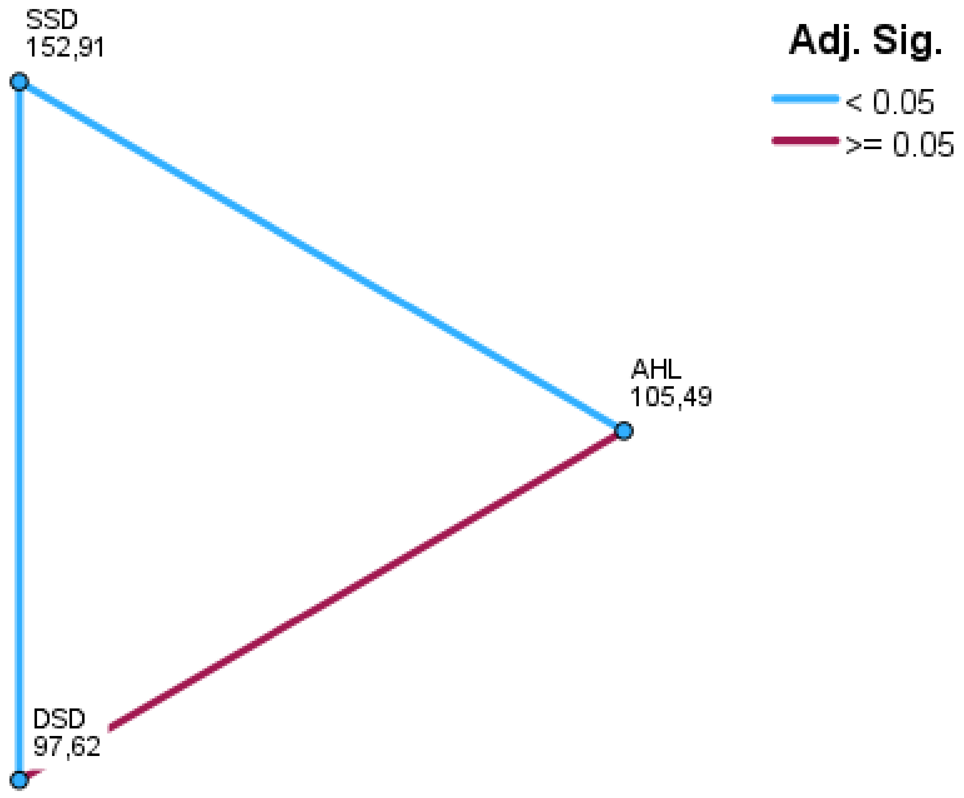

Each node shows the sample average rank of type of hearing loss.

1_NCIQ3 across type of hearing loss

Independent-Samples Kruskal-Wallis Test Summary

Total N	220
Test Statistic	11,130[a]
Degree Of Freedom	2
Asymptotic Sig.(2-sided test)	,004

a. The test statistic is adjusted for ties.

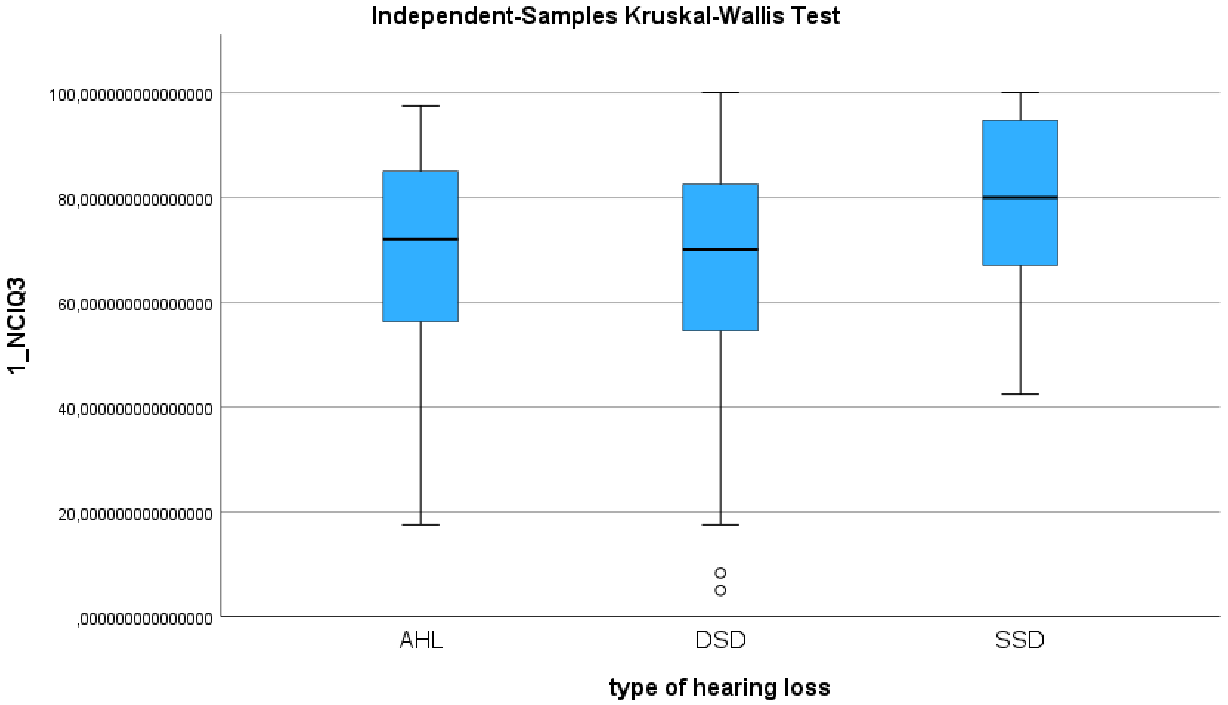

Pairwise Comparisons of type of hearing loss

Sample 1-Sample 2	Test Statistic	Std. Error	Std. Test Statistic	Sig.	Adj. Sig.[a]
DSD-AHL	5,677	10,189	,557	,577	1,000
DSD-SSD	-37,443	11,321	-3,307	<,001	,003
AHL-SSD	-31,766	12,804	-2,481	,013	,039

Each row tests the null hypothesis that the Sample 1 and Sample 2 distributions are the same.

Asymptotic significances (2-sided tests) are displayed. The significance level is ,050.

a. Significance values have been adjusted by the Bonferroni correction for multiple tests.

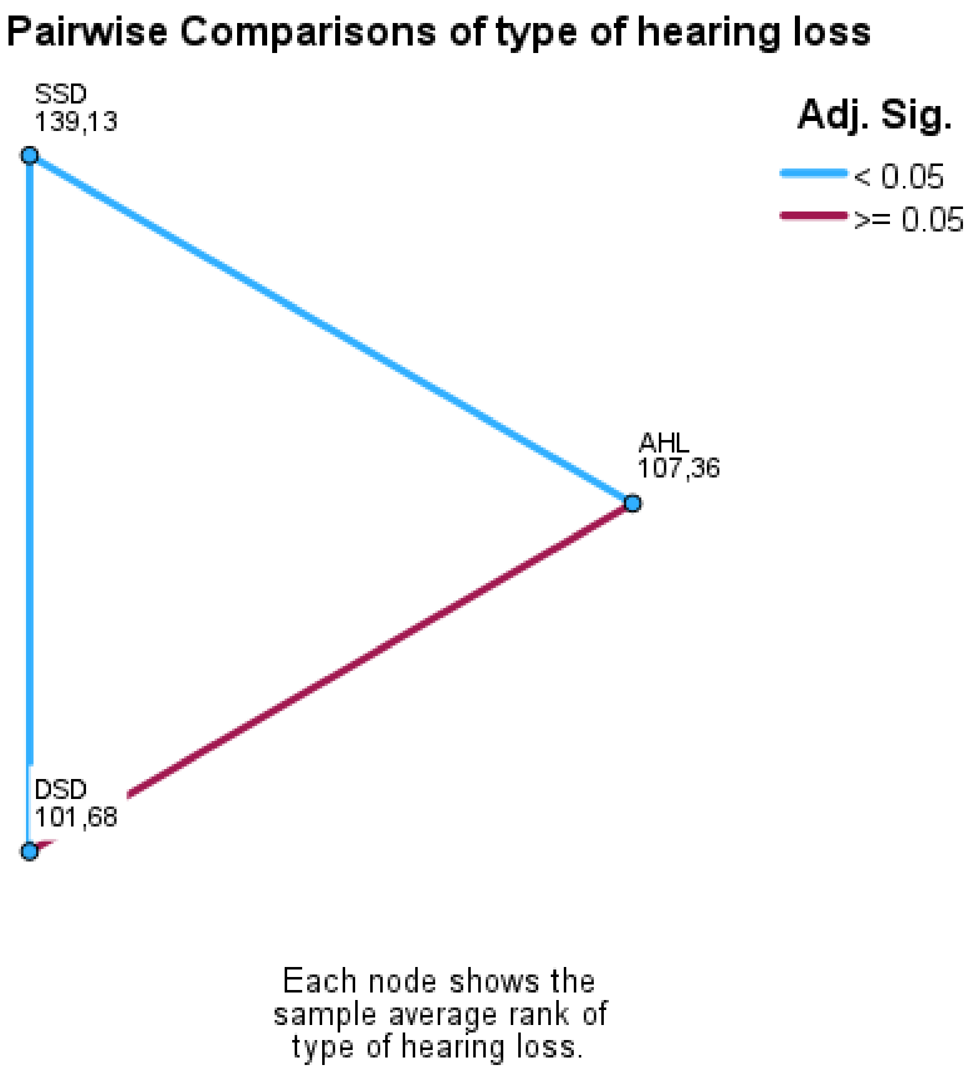

1_NCIQ4 across type of hearing loss

Independent-Samples Kruskal-Wallis Test Summary

Total N	220
Test Statistic	,727[a]
Degree Of Freedom	2
Asymptotic Sig.(2-sided test)	,695

a. The test statistic is adjusted for ties.

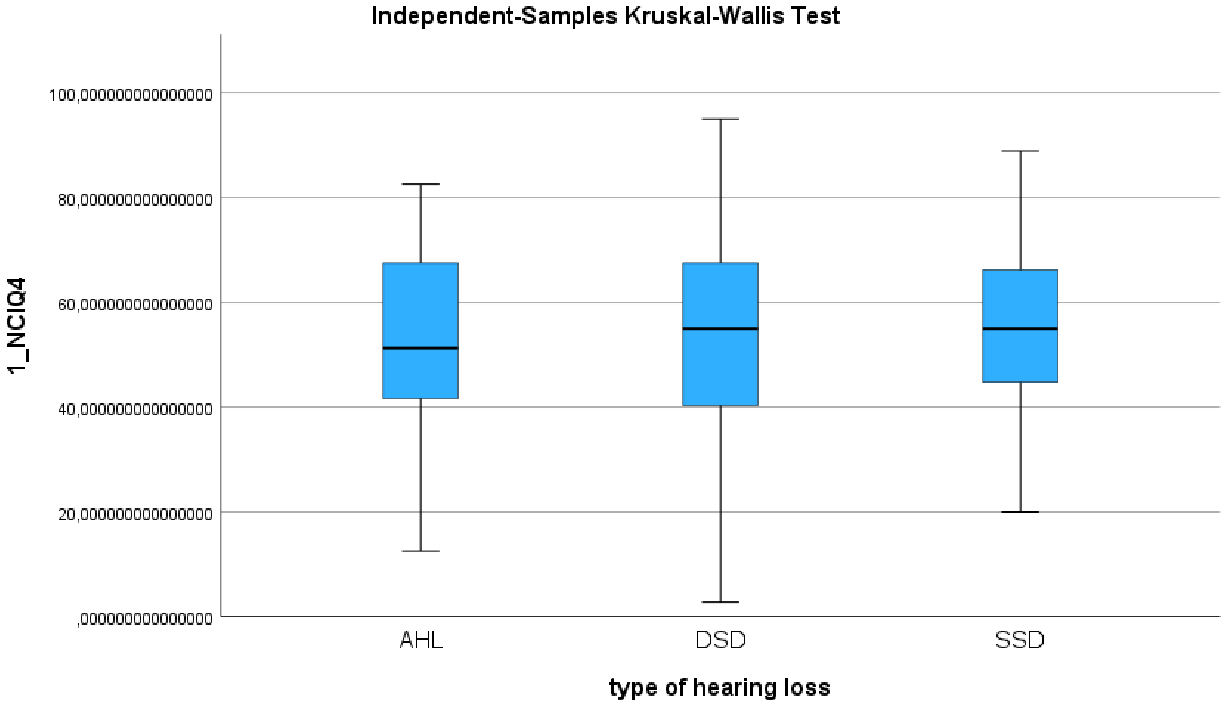

Pairwise Comparisons of type of hearing loss

Sample 1-Sample 2	Test Statistic	Std. Error	Std. Test Statistic	Sig.	Adj. Sig.[a]
AHL-DSD	-7,486	10,187	-,735	,462	1,000
AHL-SSD	-9,716	12,802	-,759	,448	1,000
DSD-SSD	-2,230	11,319	-,197	,844	1,000

Each row tests the null hypothesis that the Sample 1 and Sample 2 distributions are the same.

Asymptotic significances (2-sided tests) are displayed. The significance level is ,050.

a. Significance values have been adjusted by the Bonferroni correction for multiple tests.

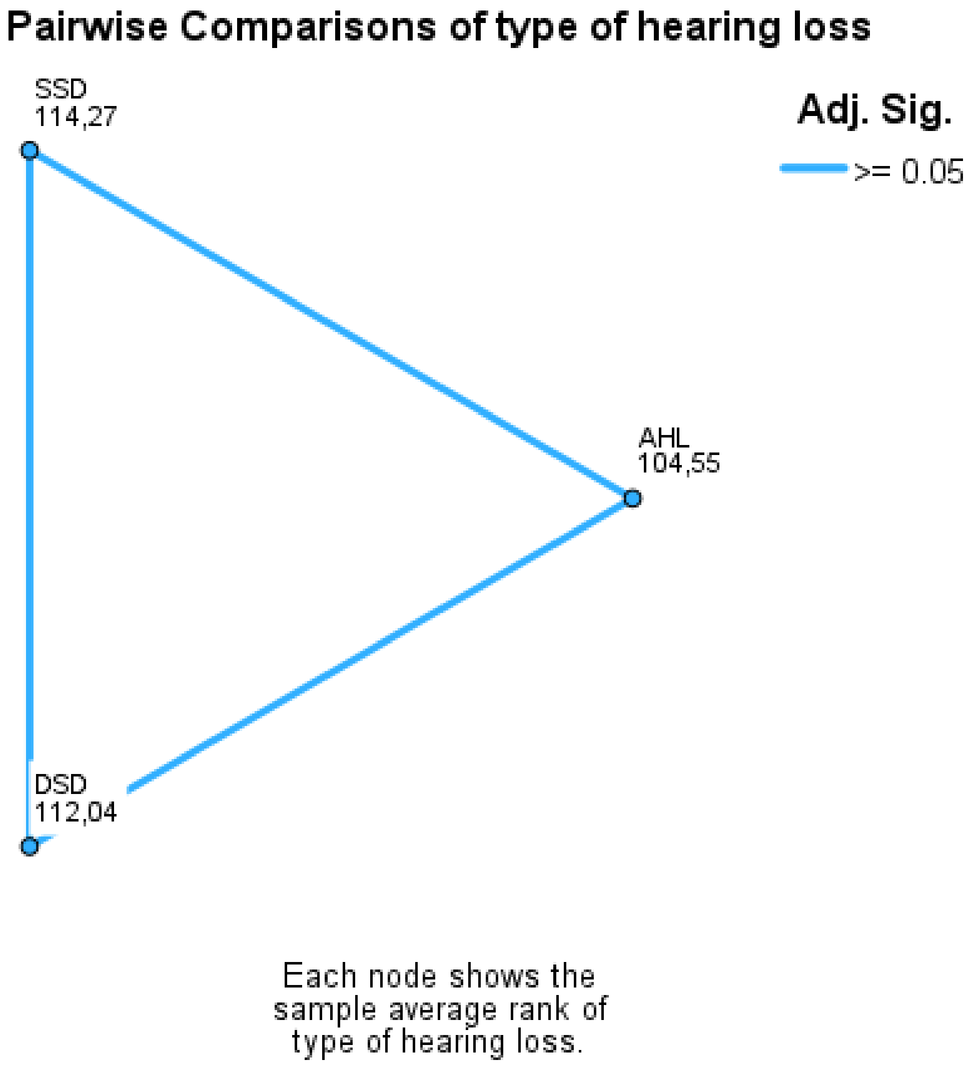

1_NCIQ5 across type of hearing loss

Independent-Samples Kruskal-Wallis Test Summary

Total N	220
Test Statistic	3,245[a]
Degree Of Freedom	2
Asymptotic Sig.(2-sided test)	,197

a. The test statistic is adjusted for ties.

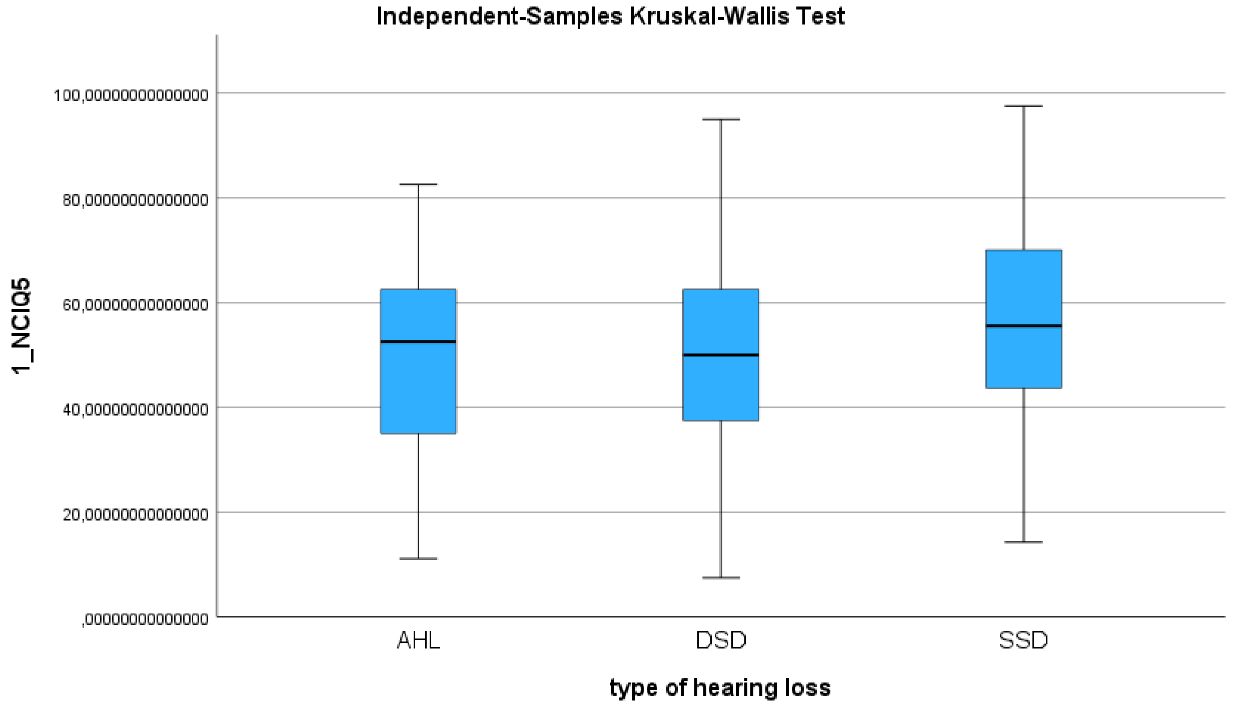

Pairwise Comparisons of type of hearing loss

Sample 1-Sample 2	Test Statistic	Std. Error	Std. Test Statistic	Sig.	Adj. Sig.[a]
AHL-DSD	-9,499	10,191	-,932	,351	1,000
AHL-SSD	-23,065	12,806	-1,801	,072	,215
DSD-SSD	-13,566	11,323	-1,198	,231	,693

Each row tests the null hypothesis that the Sample 1 and Sample 2 distributions are the same.
Asymptotic significances (2-sided tests) are displayed. The significance level is ,050.
a. Significance values have been adjusted by the Bonferroni correction for multiple tests.

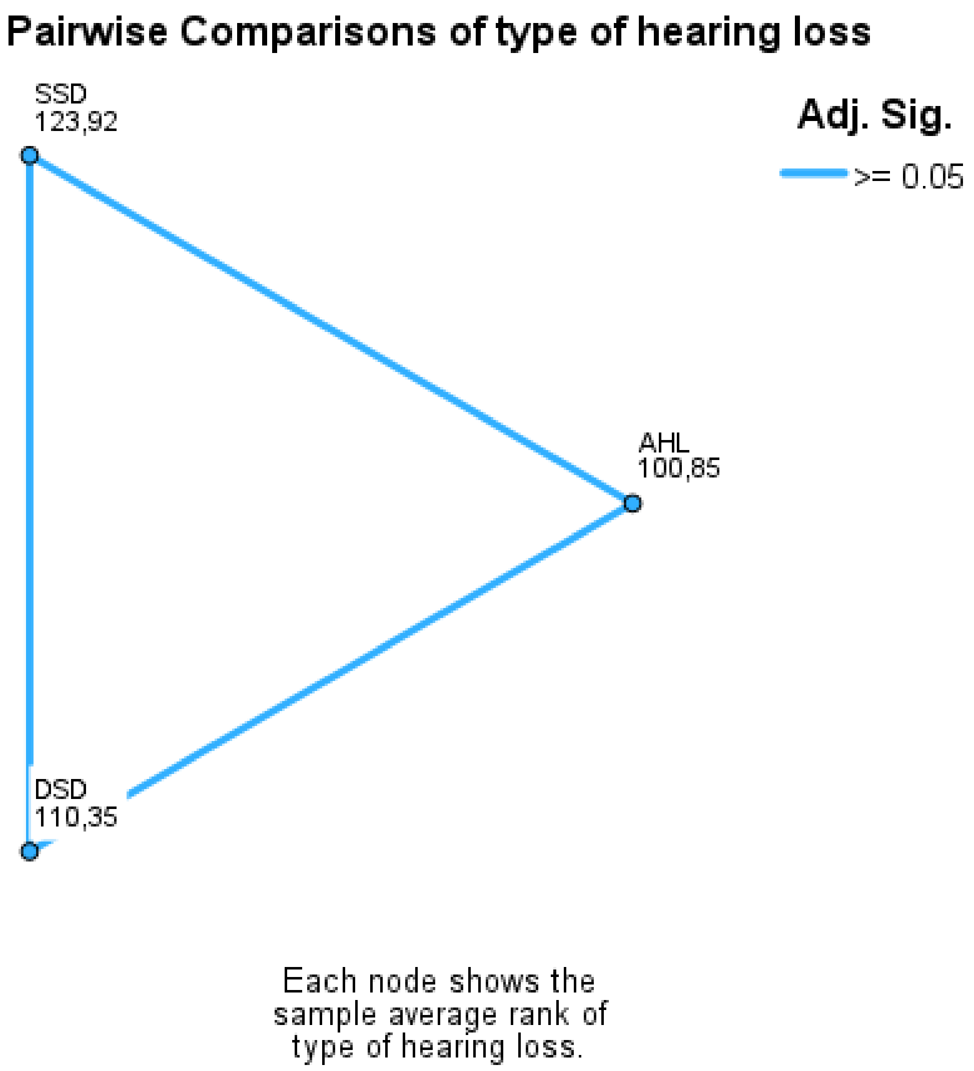

1_NCIQ6 across type of hearing loss

Independent-Samples Kruskal-Wallis Test Summary

Total N	220
Test Statistic	5,893[a]
Degree Of Freedom	2
Asymptotic Sig.(2-sided test)	,053

a. The test statistic is adjusted for ties.

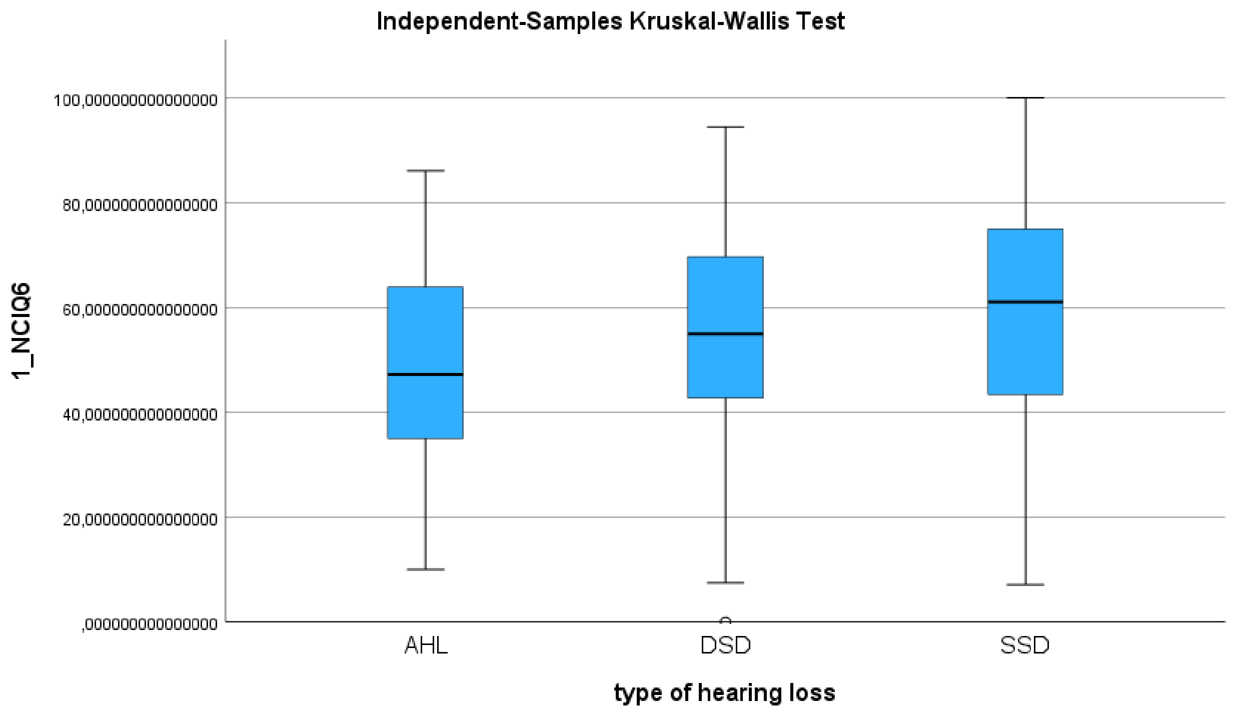

Pairwise Comparisons of type of hearing loss

Sample 1-Sample 2	Test Statistic	Std. Error	Std. Test Statistic	Sig.	Adj. Sig.[a]
AHL-DSD	-17,842	10,191	-1,751	,080	,240
AHL-SSD	-30,191	12,807	-2,357	,018	,055
DSD-SSD	-12,350	11,324	-1,091	,275	,826

Each row tests the null hypothesis that the Sample 1 and Sample 2 distributions are the same.
Asymptotic significances (2-sided tests) are displayed. The significance level is ,050.
a. Significance values have been adjusted by the Bonferroni correction for multiple tests.

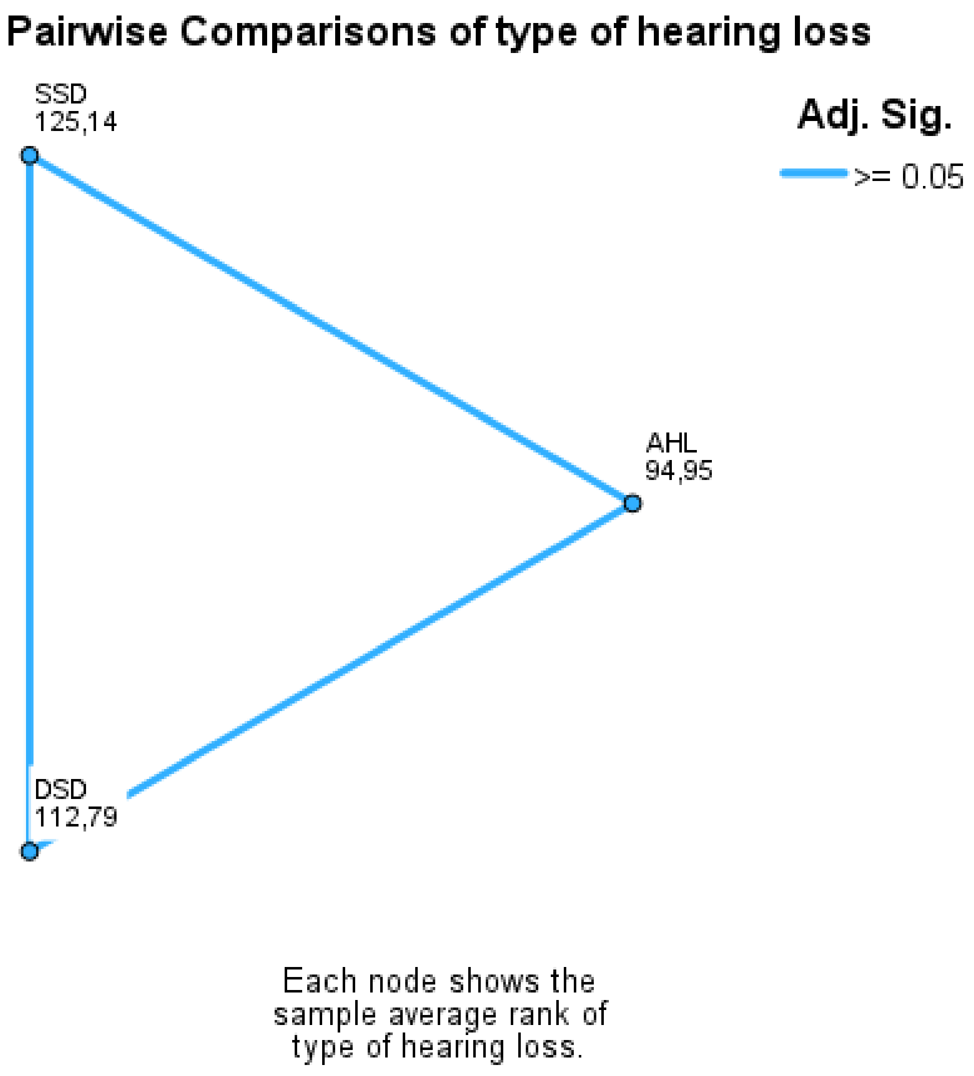

1_NCIQtotal across type of hearing loss

Independent-Samples Kruskal-Wallis Test Summary

Total N	220
Test Statistic	8,603[a]
Degree Of Freedom	2
Asymptotic Sig.(2-sided test)	,014

a. The test statistic is adjusted for ties.

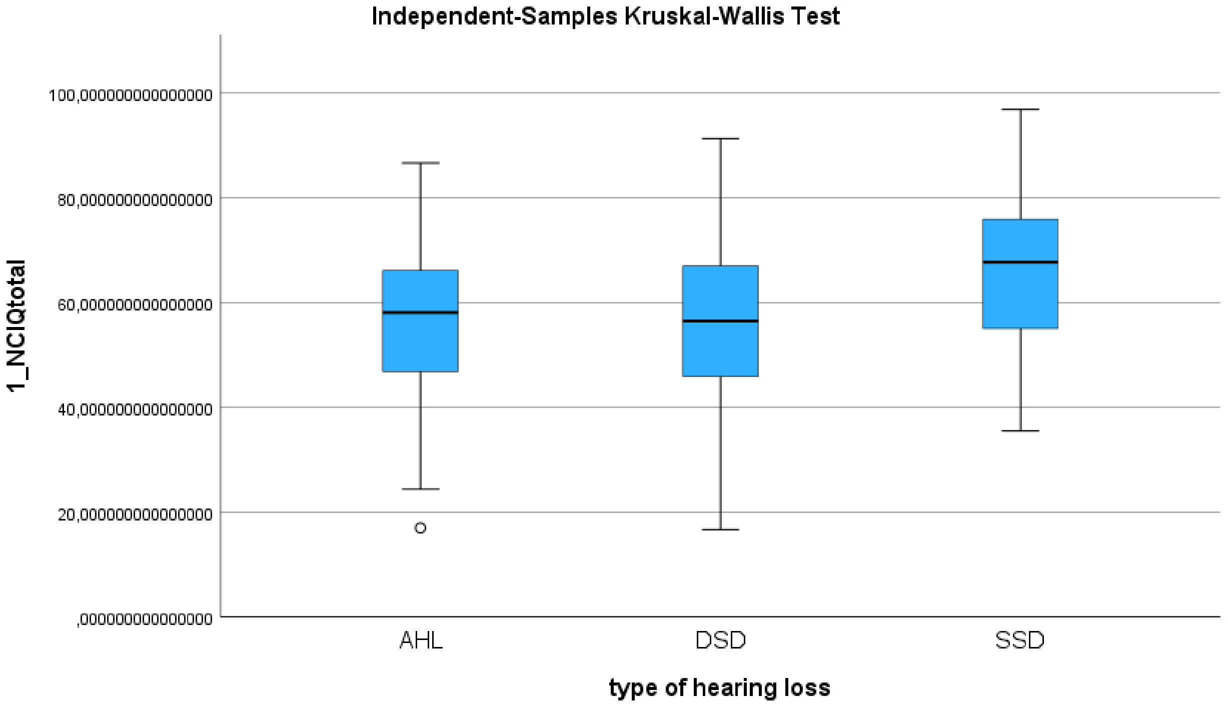

Pairwise Comparisons of type of hearing loss

Sample 1-Sample 2	Test Statistic	Std. Error	Std. Test Statistic	Sig.	Adj. Sig.[a]
AHL-DSD	-2,249	10,193	-,221	,825	1,000
AHL-SSD	-33,163	12,809	-2,589	,010	,029
DSD-SSD	-30,915	11,326	-2,730	,006	,019

Each row tests the null hypothesis that the Sample 1 and Sample 2 distributions are the same.

Asymptotic significances (2-sided tests) are displayed. The significance level is ,050.

a. Significance values have been adjusted by the Bonferroni correction for multiple tests.

Pairwise Comparisons of type of hearing loss

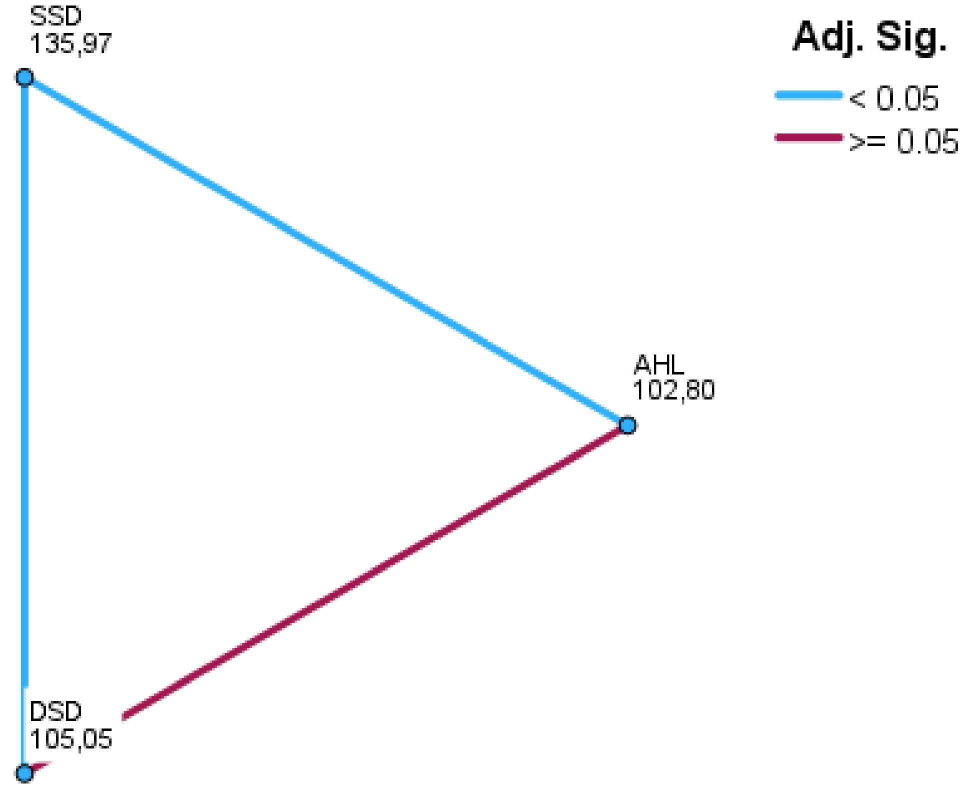

Each node shows the sample average rank of type of hearing loss.

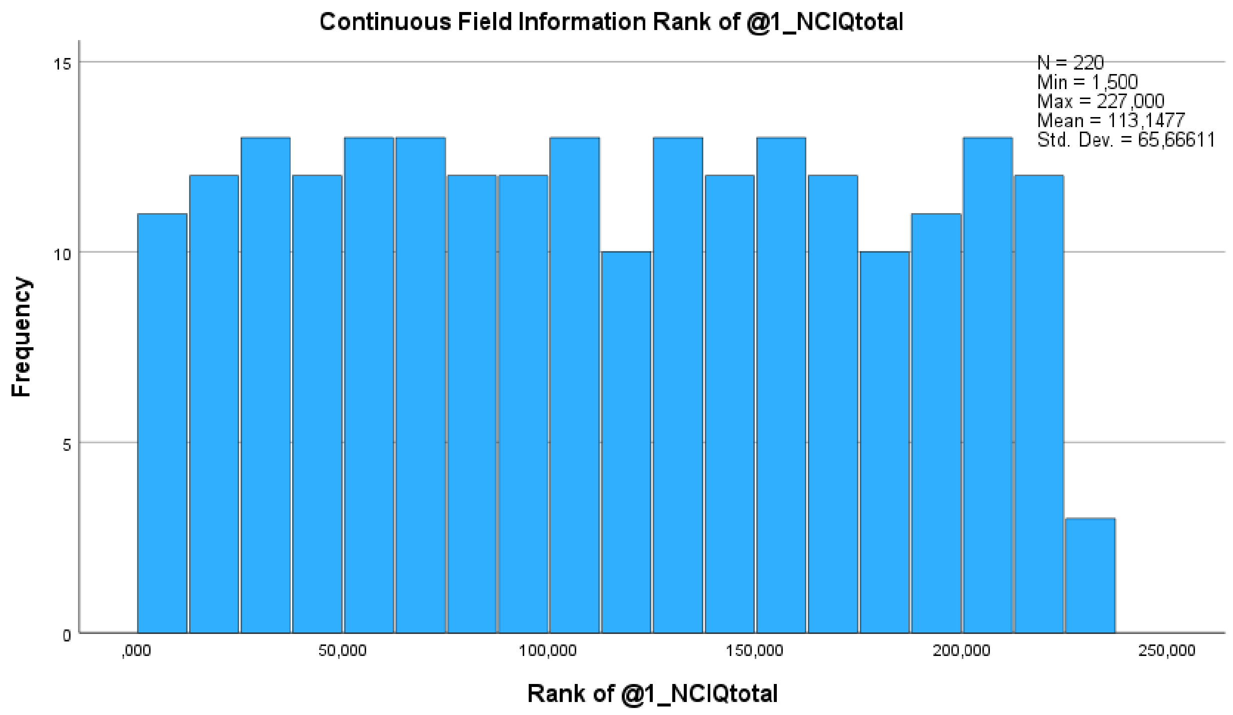
Continuous Field Information Rank of @1_NCIQtotal
N = 220
Min = 1,500
Max = 227,000
Mean = 113,1477
Std. Dev. = 65,66611
Frequency
Rank of @1_NCIQtotal

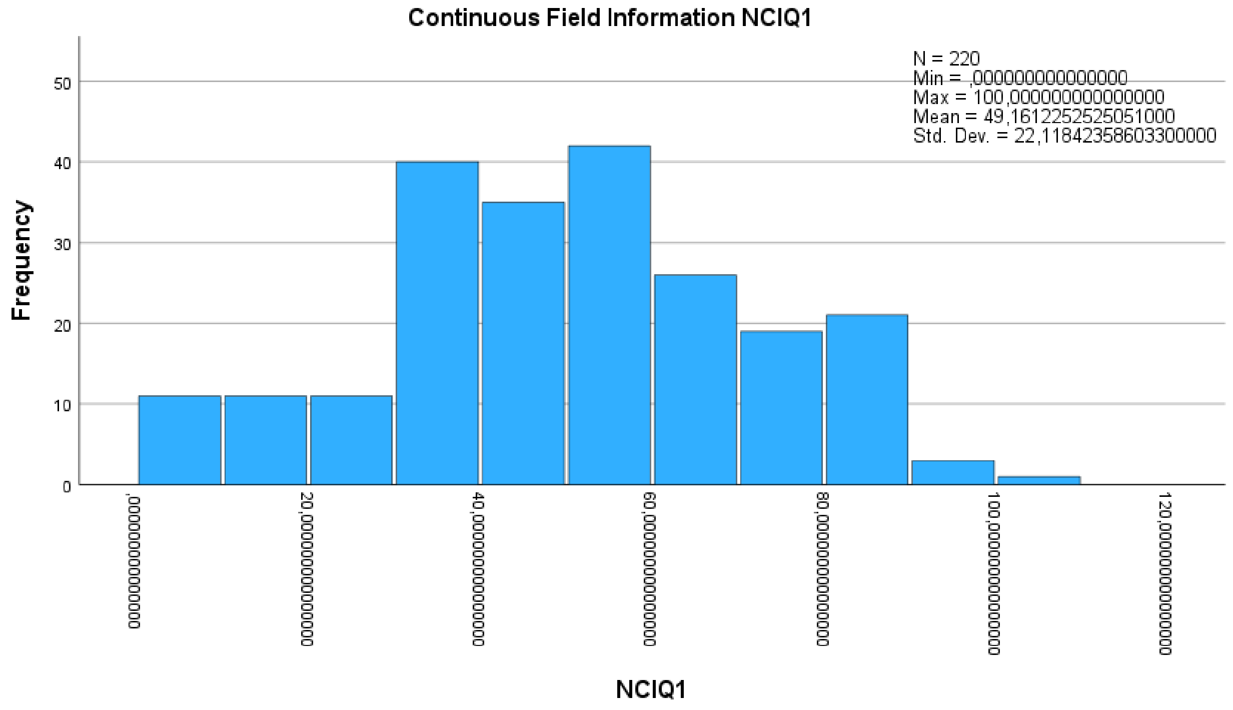
Continuous Field Information NCIQ1
N = 220
Min = ,000000000000000
Max = 100,000000000000000
Mean = 49,1612252525051000
Std. Dev. = 22,11842358603300000
Frequency
NCIQ1

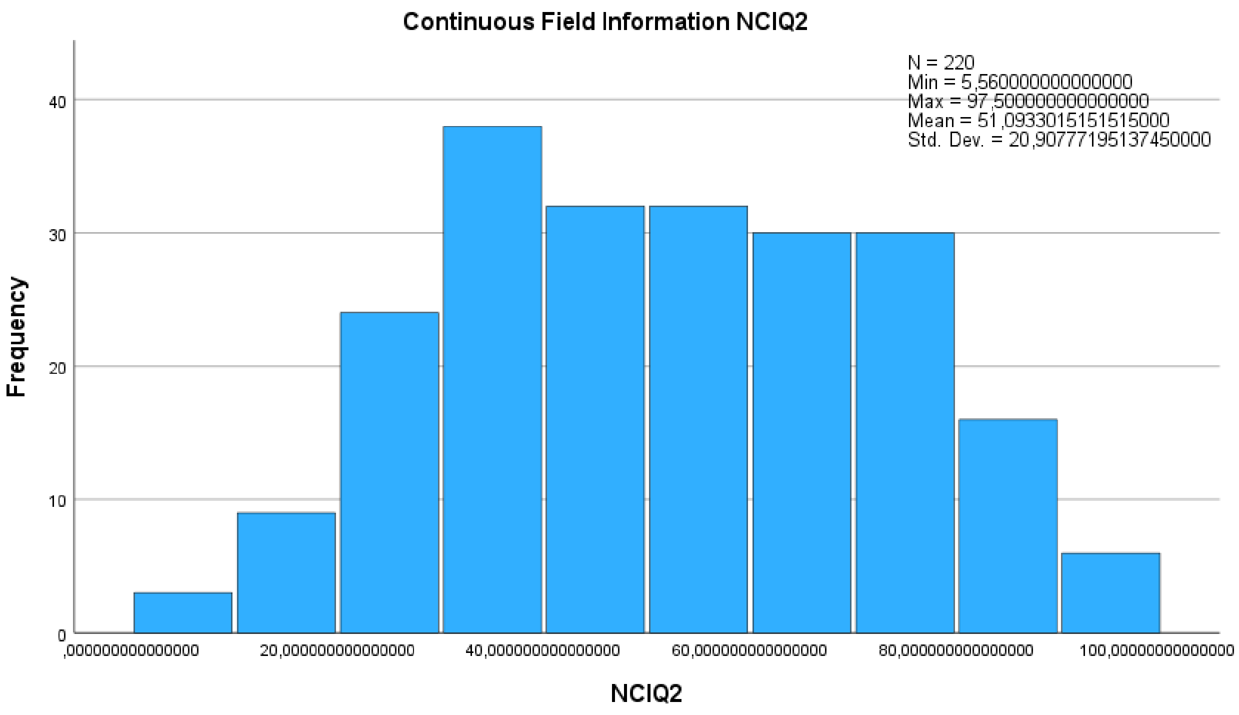
Continuous Field Information NCIQ2
N = 220
Min = 5,560000000000000
Max = 97,500000000000000
Mean = 51,0933015151515000
Std. Dev. = 20,90777195137450000
Frequency
0
10
20
30
40
,000000000000000
20,000000000000000
40,000000000000000
60,000000000000000
80,000000000000000
100,000000000000000
NCIQ2

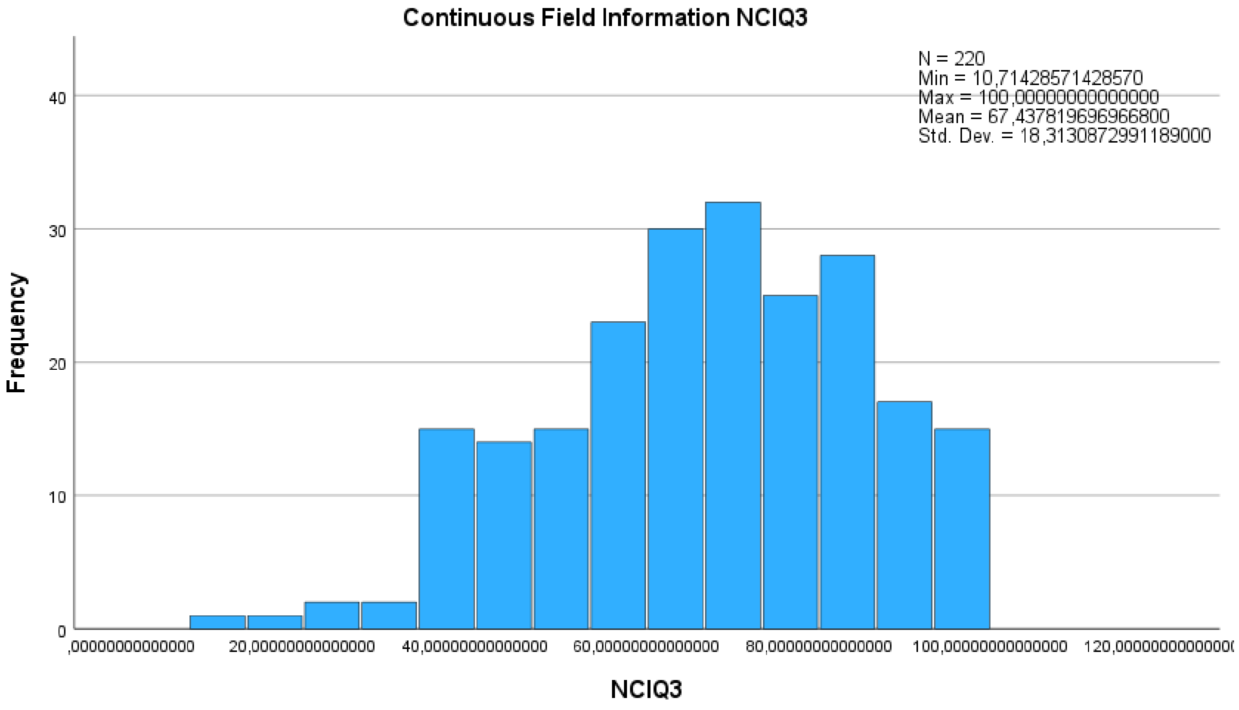
Continuous Field Information NCIQ3
N = 220
Min = 10,71428571428570
Max = 100,00000000000000
Mean = 67,437819696966800
Std. Dev. = 18,3130872991189000
Frequency
0
10
20
30
40
,00000000000000
20,00000000000000
40,00000000000000
60,00000000000000
80,00000000000000
100,00000000000000
NCIQ3

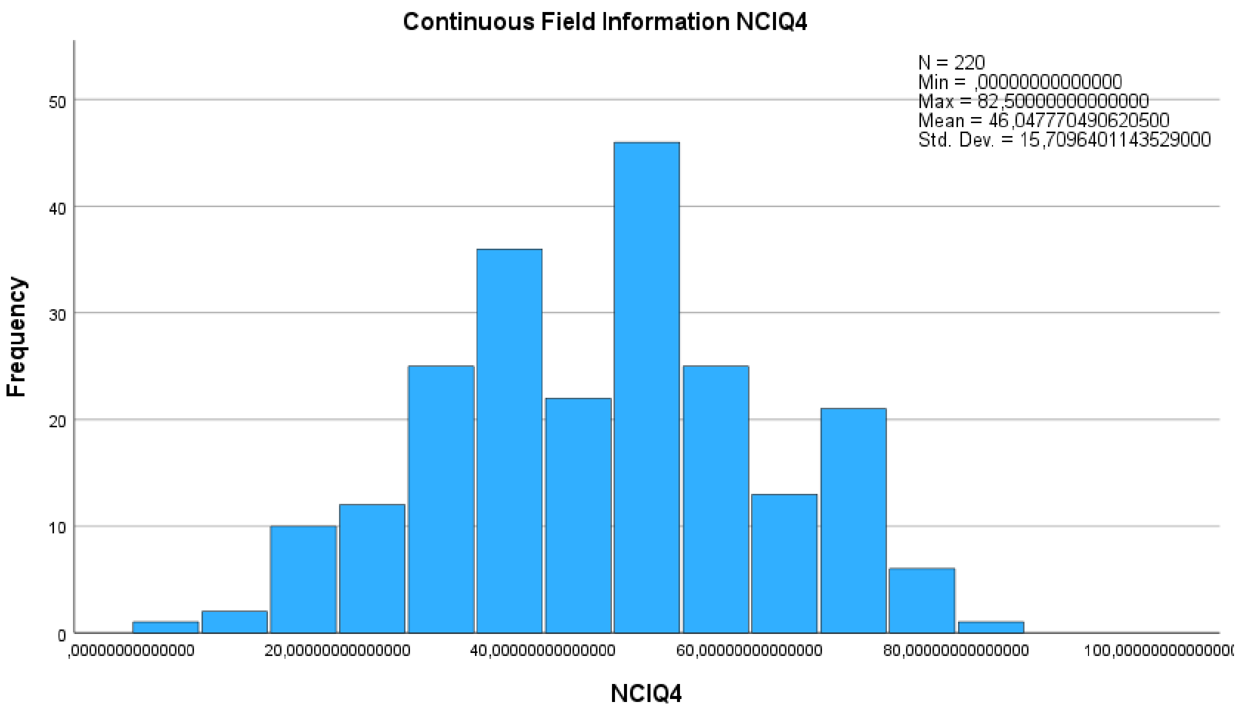
Continuous Field Information NCIQ4
N = 220
Min = ,00000000000000
Max = 82,50000000000000
Mean = 46,047770490620500
Std. Dev. = 15,7096401143529000
Frequency
NCIQ4

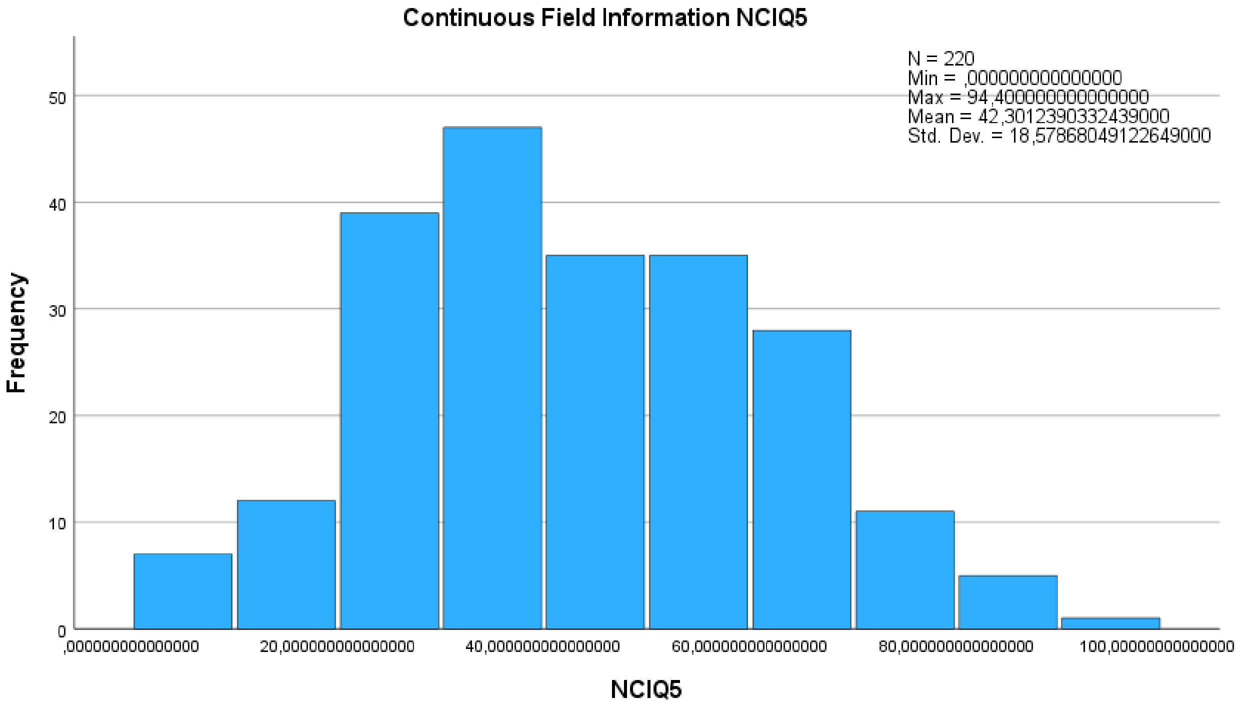
Continuous Field Information NCIQ5
N = 220
Min = ,000000000000000
Max = 94,400000000000000
Mean = 42,3012390332439000
Std. Dev. = 18,5786804912264900
Frequency
NCIQ5

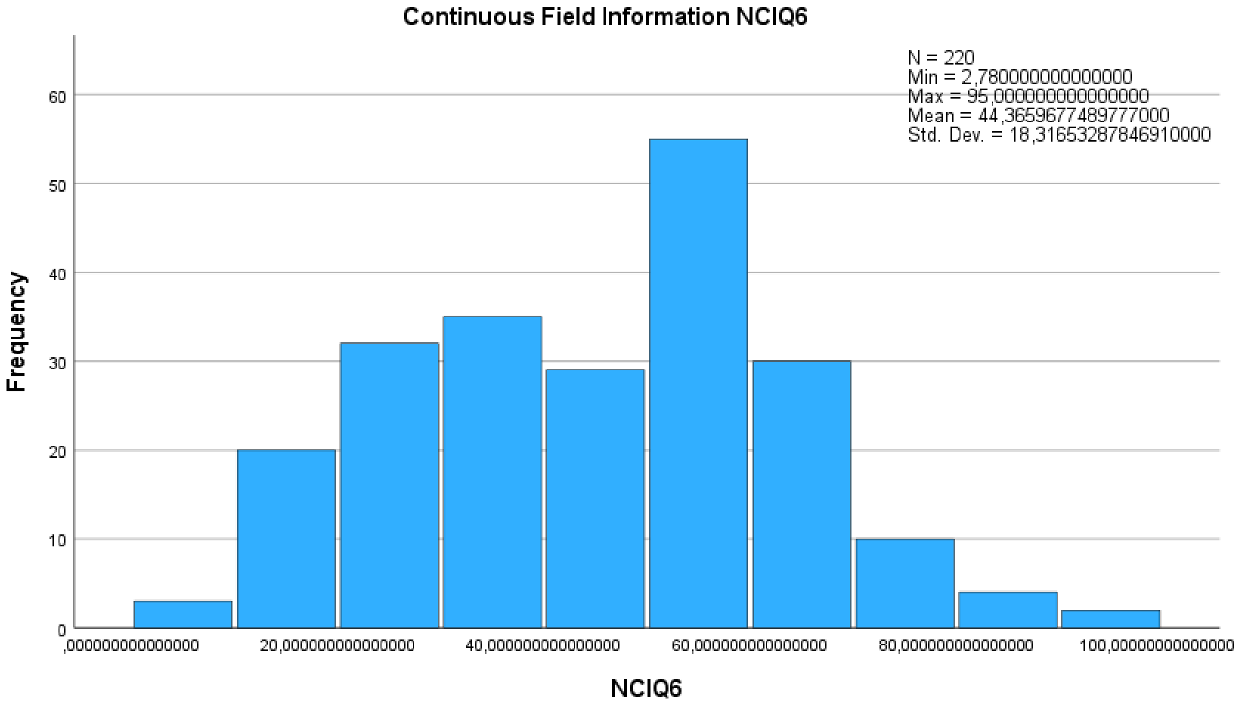
Continuous Field Information NCIQ6
N = 220
Min = 2,780000000000000
Max = 95,000000000000000
Mean = 44,3659677489777000
Std. Dev. = 18,31653287846910000
Frequency
0
10
20
30
40
50
60
,000000000000000
20,000000000000000
40,000000000000000
60,000000000000000
80,000000000000000
100,000000000000000
NCIQ6

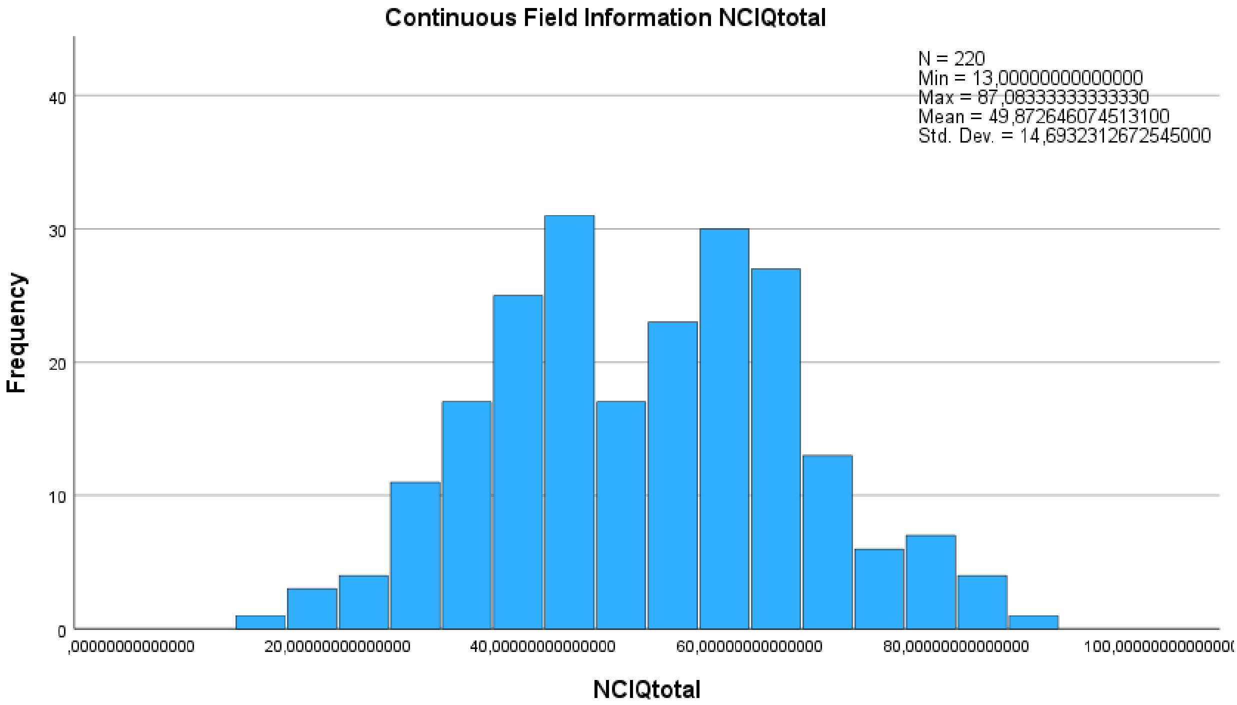
Continuous Field Information NCIQtotal
N = 220
Min = 13,00000000000000
Max = 87,08333333333330
Mean = 49,872646074513100
Std. Dev. = 14,6932312672545000
Frequency
0
10
20
30
40
,00000000000000
20,00000000000000
40,00000000000000
60,00000000000000
80,00000000000000
NCIQtotal

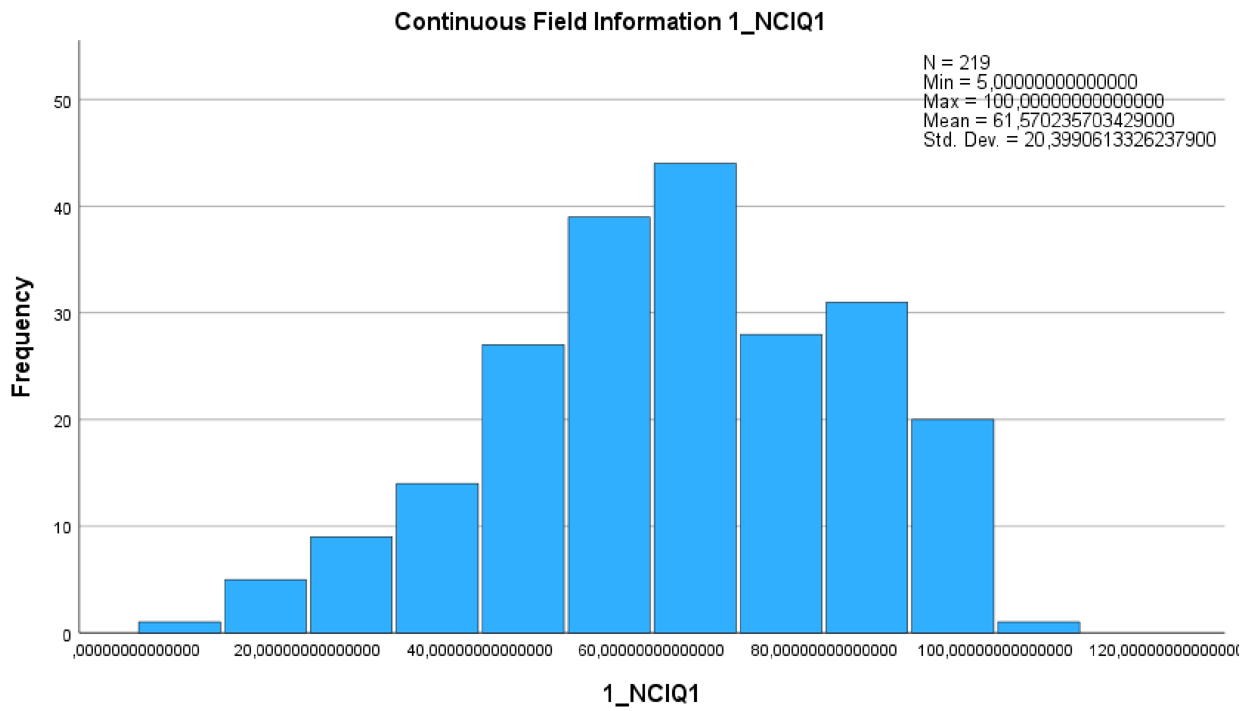
Continuous Field Information 1_NCIQ1
N = 219
Min = 5,00000000000000
Max = 100,00000000000000
Mean = 61,570235703429000
Std. Dev. = 20,3990613326237900
Frequency
0
10
20
30
40
50
,000000000000000
20,000000000000000
40,000000000000000
60,000000000000000
80,000000000000000
100,000000000000000
1_NCIQ1

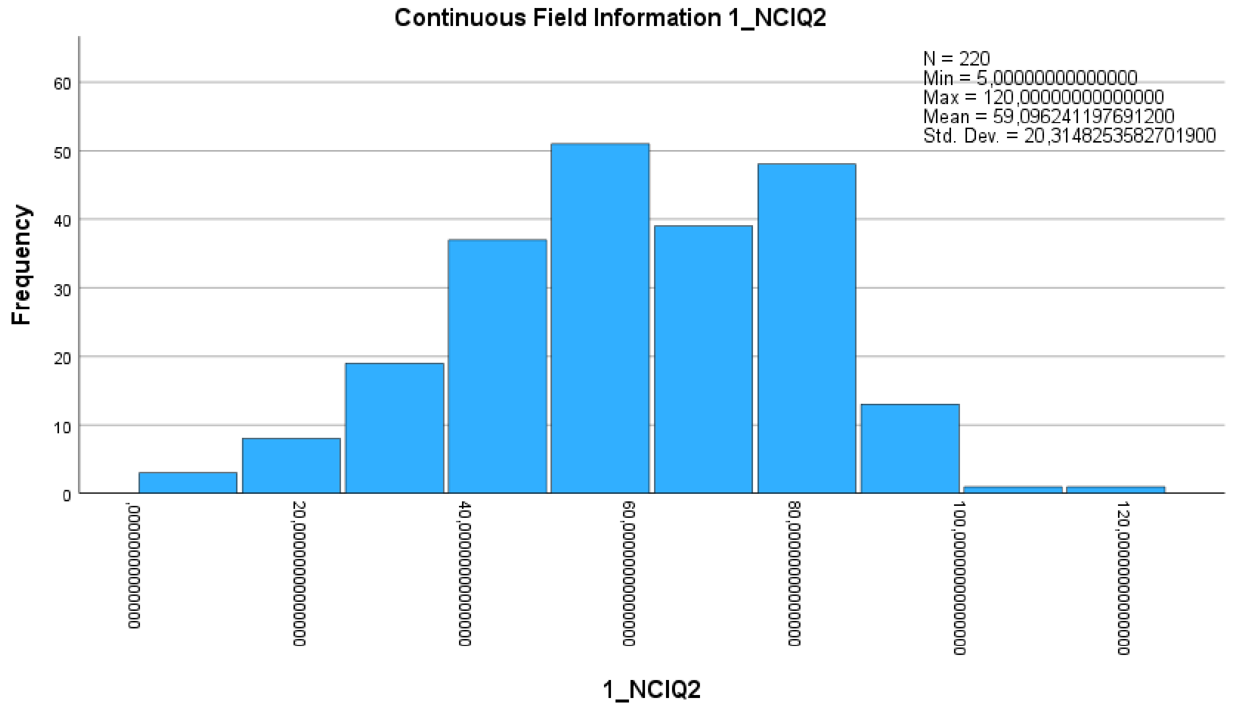
Continuous Field Information 1_NCIQ2
N = 220
Min = 5,00000000000000
Max = 120,00000000000000
Mean = 59,096241197691200
Std. Dev. = 20,3148253582701900
Frequency
0
10
20
30
40
50
60
1_NCIQ2

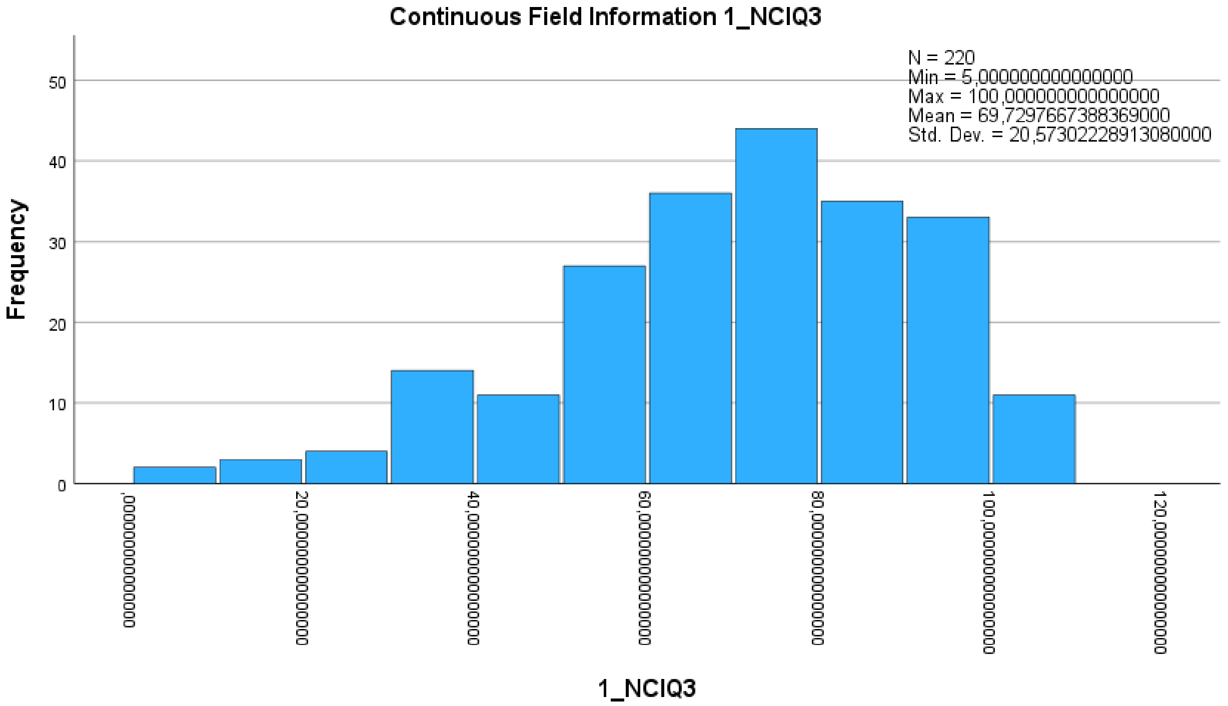
Continuous Field Information 1_NCIQ3
N = 220
Min = 5,000000000000000
Max = 100,000000000000000
Mean = 69,7297667388369000
Std. Dev. = 20,57302228913080000
Frequency
50
40
30
20
10
0
,0000000000000000
20,0000000000000000
40,0000000000000000
60,0000000000000000
80,0000000000000000
100,0000000000000000
120,0000000000000000
1_NCIQ3

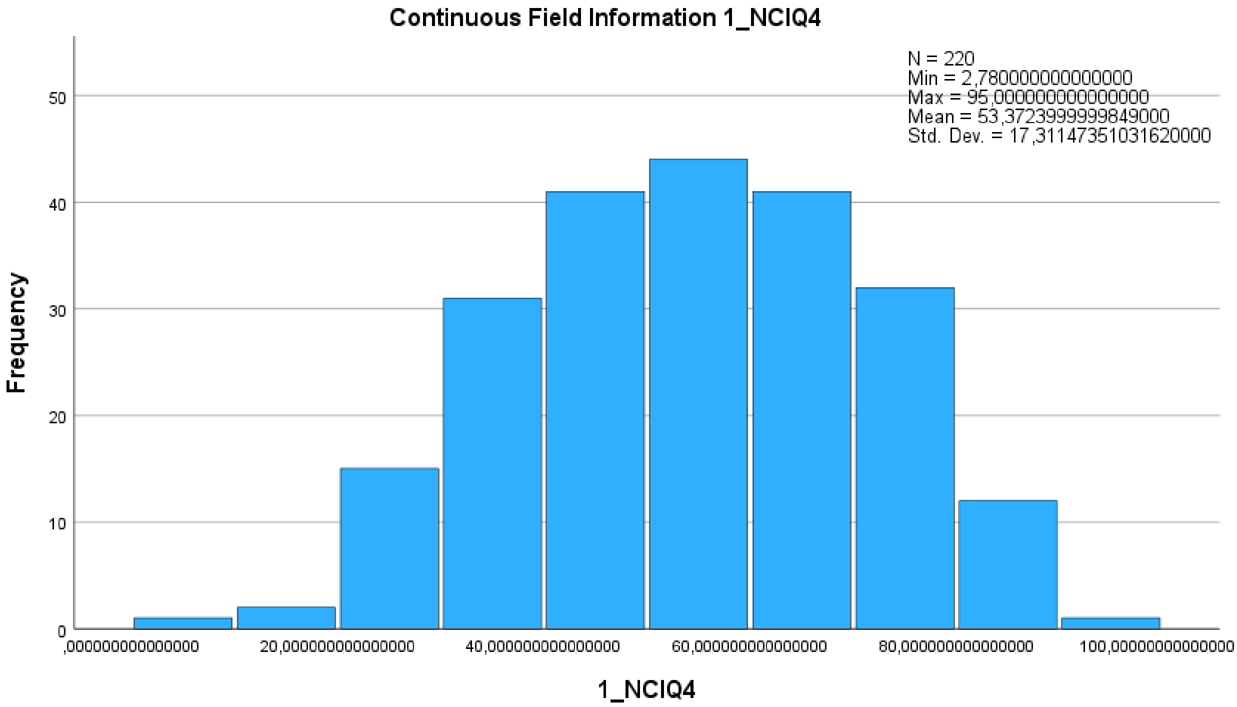
Continuous Field Information 1_NCIQ4
N = 220
Min = 2,780000000000000
Max = 95,000000000000000
Mean = 53,3723999999849000
Std. Dev. = 17,31147351031620000
Frequency
50
40
30
20
10
0
,0000000000000000
20,0000000000000000
40,0000000000000000
60,0000000000000000
80,0000000000000000
100,0000000000000000
1_NCIQ4

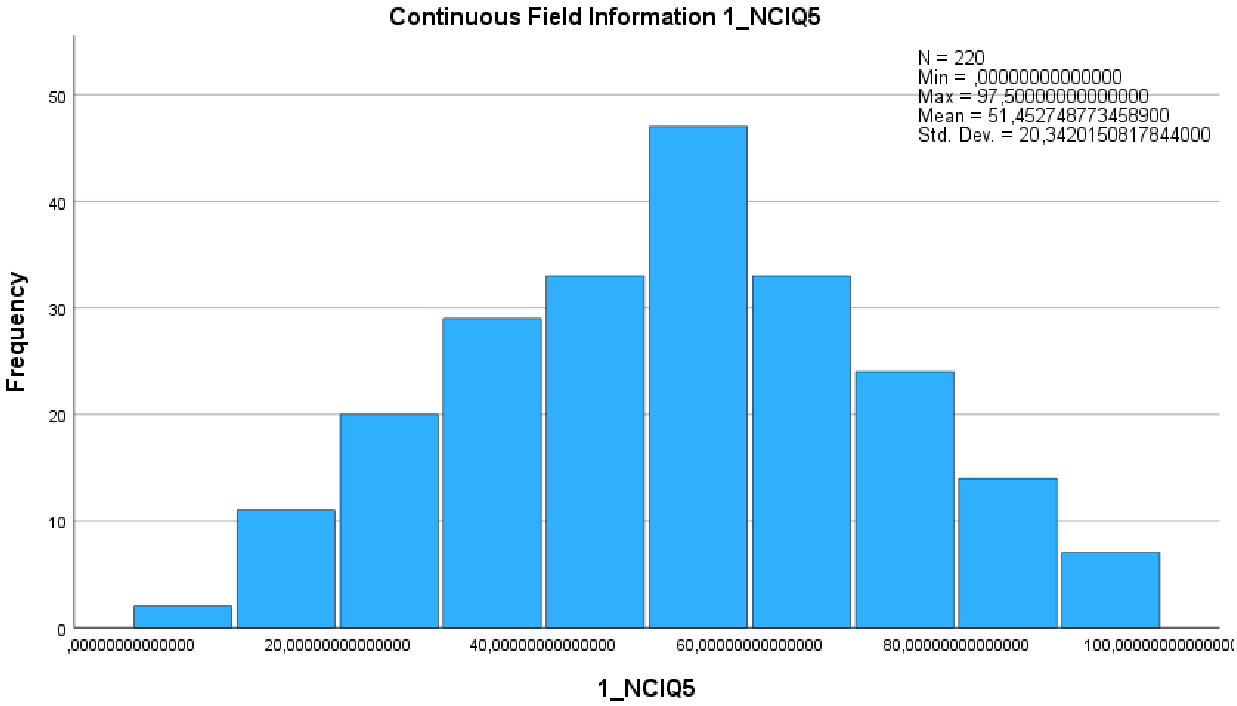
Continuous Field Information 1_NCIQ5
N = 220
Min = ,00000000000000
Max = 97,50000000000000
Mean = 51,452748773458900
Std. Dev. = 20,3420150817844000
Frequency
50
40
30
20
10
0
,00000000000000
20,00000000000000
40,00000000000000
60,00000000000000
80,00000000000000
1_NCIQ5

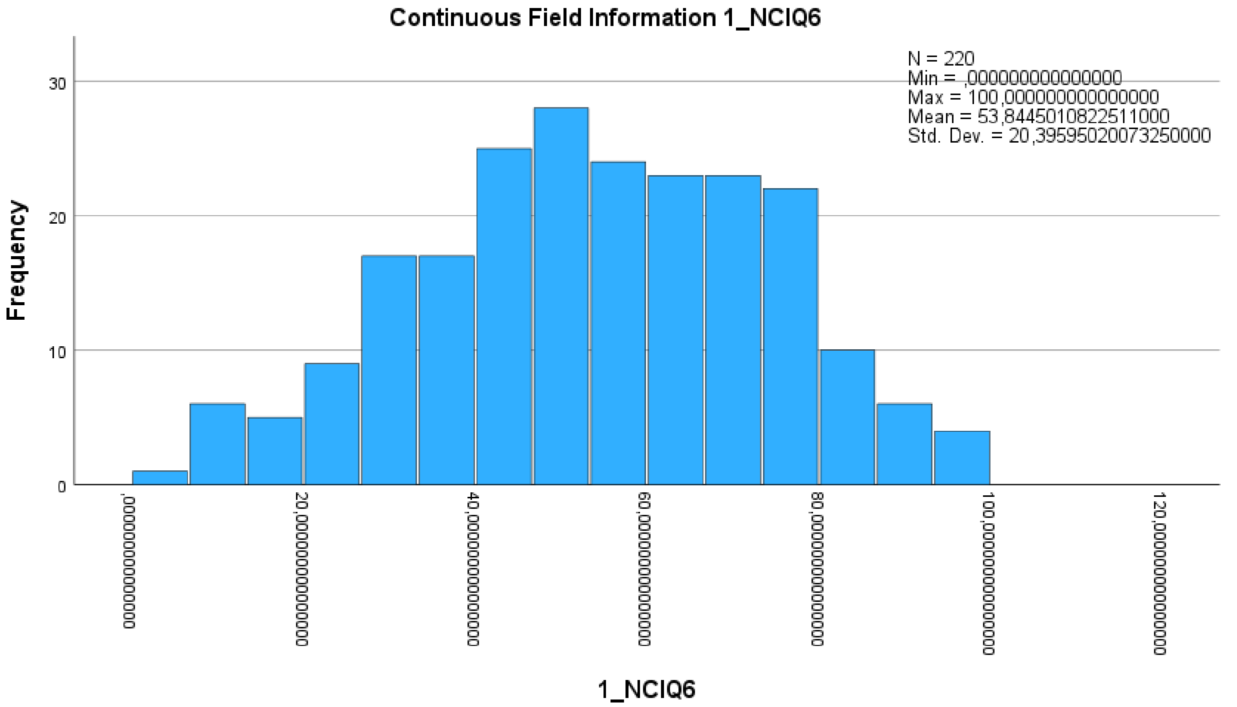
Continuous Field Information 1_NCIQ6
N = 220
Min = ,000000000000000
Max = 100,000000000000000
Mean = 53,8445010822511000
Std. Dev. = 20,39595020073250000
Frequency
30
20
10
0
1_NCIQ6

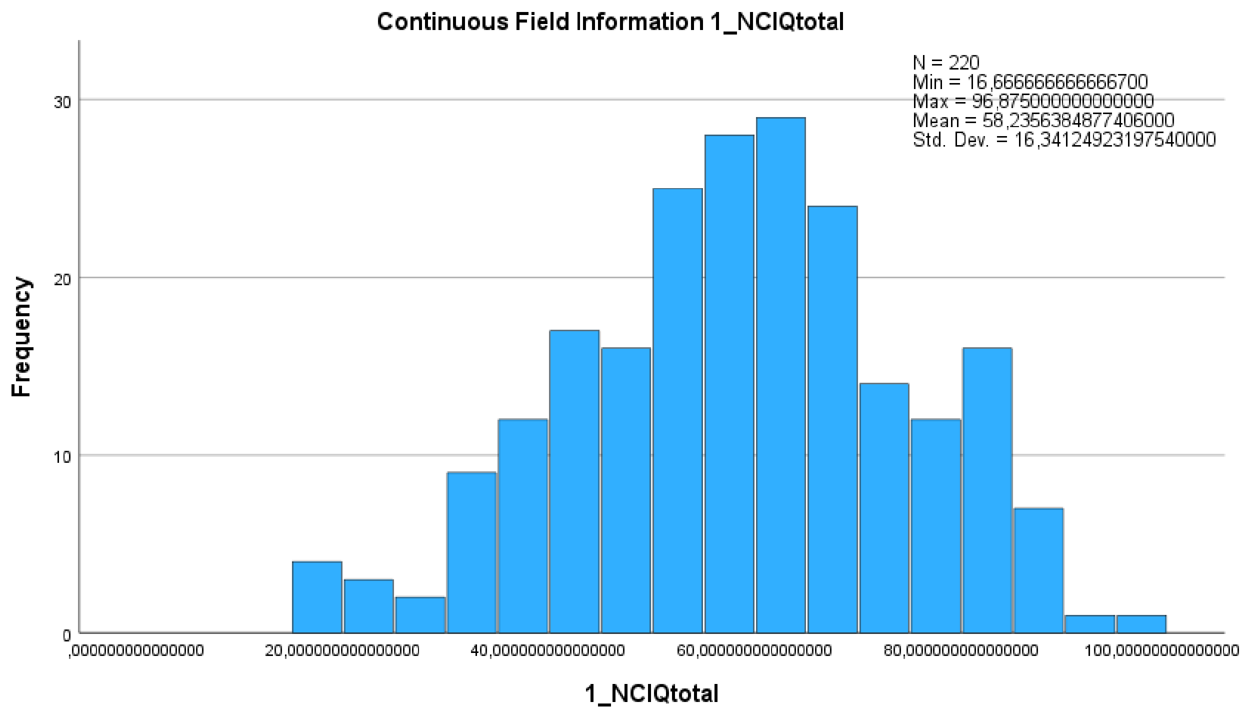
Continuous Field Information 1_NCIQtotal
N = 220
Min = 16,666666666666700
Max = 96,875000000000000
Mean = 58,2356384877406000
Std. Dev. = 16,34124923197540000
Frequency
0
10
20
30
,0000000000000000
20,0000000000000000
40,0000000000000000
60,0000000000000000
80,0000000000000000
100,000000000000000
1_NCIQtotal

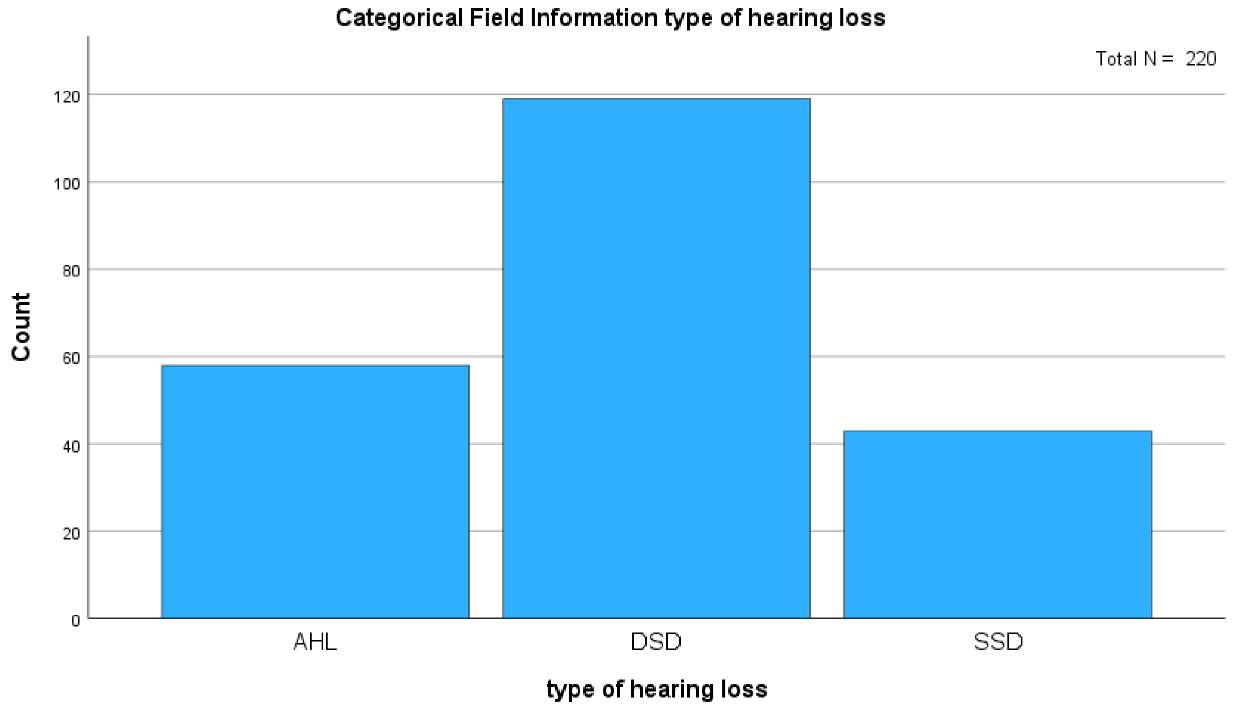
Categorical Field Information type of hearing loss
Total N = 220
Count
0
20
40
60
80
100
120
AHL
DSD
SSD
type of hearing loss
