# Supplementary material for: Changes in Speech Intelligibility, Health-Related Quality of Life, Depressive Symptoms, Anxiety, Perceived Stress, and Tinnitus-Induced Distress, in a Cohort of 227 Adults One Year After Cochlear Implantation: A Decade of Experience from a Single Tertiary Center
Source: J Clin Med. 2025 Nov 17;14(22):8143. doi: 10.3390/jcm14228143 (PMC12653197; doi:10.3390/jcm14228143)
Supplement: Supplementary file 1 [file jcm-14-08143-s001.zip › S2.pdf]

S2: Kruskal-Wallis Test: the comparison between the pre- and post-scores of ADSL, GAD and PSQ for AHL, DSD, and SSD groups.

Hypothesis Test Summary

	Null Hypothesis	Test	Sig.[a,b]	Decision
1	The distribution of ADSL is the same across categories of type of hearing loss.	Independent-Samples Kruskal-Wallis Test	,825	Retain the null hypothesis.
2	The distribution of 1_ADSL is the same across categories of type of hearing loss.	Independent-Samples Kruskal-Wallis Test	,809	Retain the null hypothesis.
3	The distribution of GAD is the same across categories of type of hearing loss.	Independent-Samples Kruskal-Wallis Test	,214	Retain the null hypothesis.
4	The distribution of 1_GAD is the same across categories of type of hearing loss.	Independent-Samples Kruskal-Wallis Test	,748	Retain the null hypothesis.
5	The distribution of PSQ is the same across categories of type of hearing loss.	Independent-Samples Kruskal-Wallis Test	,092	Retain the null hypothesis.
6	The distribution of 1_PSQ is the same across categories of type of hearing loss.	Independent-Samples Kruskal-Wallis Test	,868	Retain the null hypothesis.

a. The significance level is ,050.

b. Asymptotic significance is displayed.

Independent-Samples Kruskal-Wallis Test

ADSL across type of hearing loss

Independent-Samples Kruskal-Wallis Test Summary

Total N	218

Test Statistic	,385[a]
Degree Of Freedom	2
Asymptotic Sig.(2-sided test)	,825

a. The test statistic is adjusted for ties.

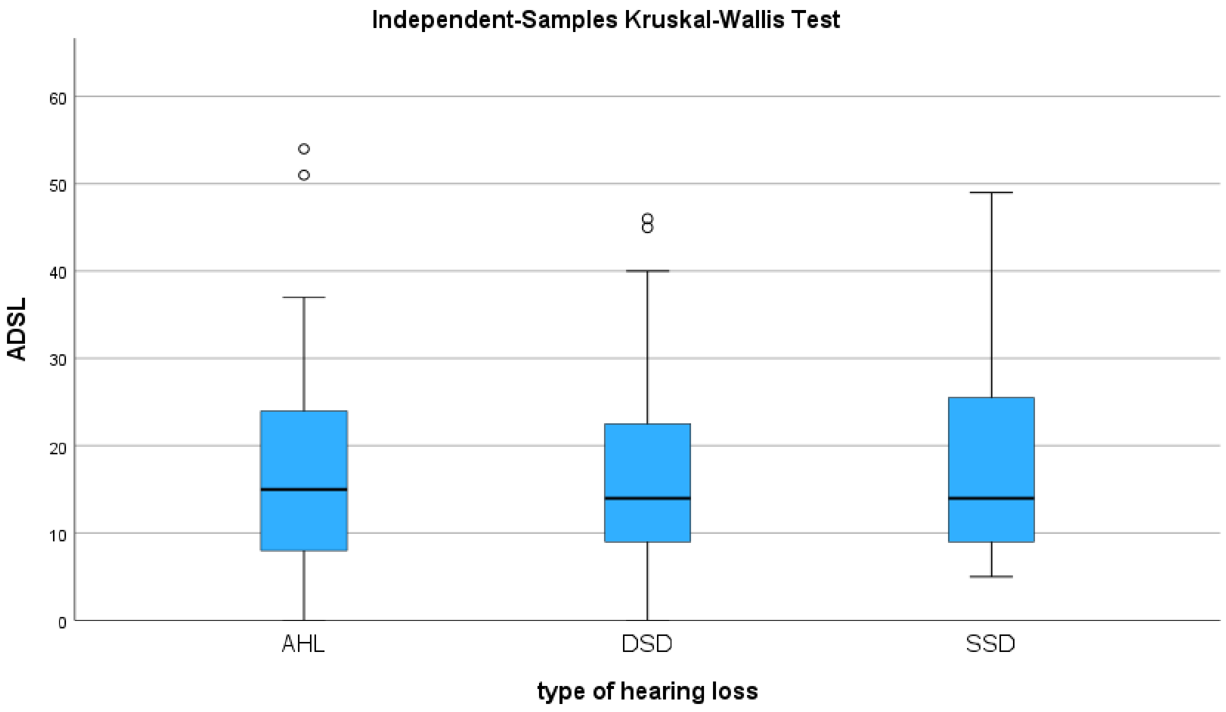

Pairwise Comparisons of type of hearing loss

Sample 1-Sample 2	Test Statistic	Std. Error	Std. Test Statistic	Sig.	Adj. Sig.[a]
DSD-AHL	1,533	10,213	,150	,881	1,000
DSD-SSD	-6,951	11,214	-,620	,535	1,000
AHL-SSD	-5,418	12,779	-,424	,672	1,000

Each row tests the null hypothesis that the Sample 1 and Sample 2 distributions are the same.

Asymptotic significances (2-sided tests) are displayed. The significance level is ,050.

a. Significance values have been adjusted by the Bonferroni correction for multiple tests.

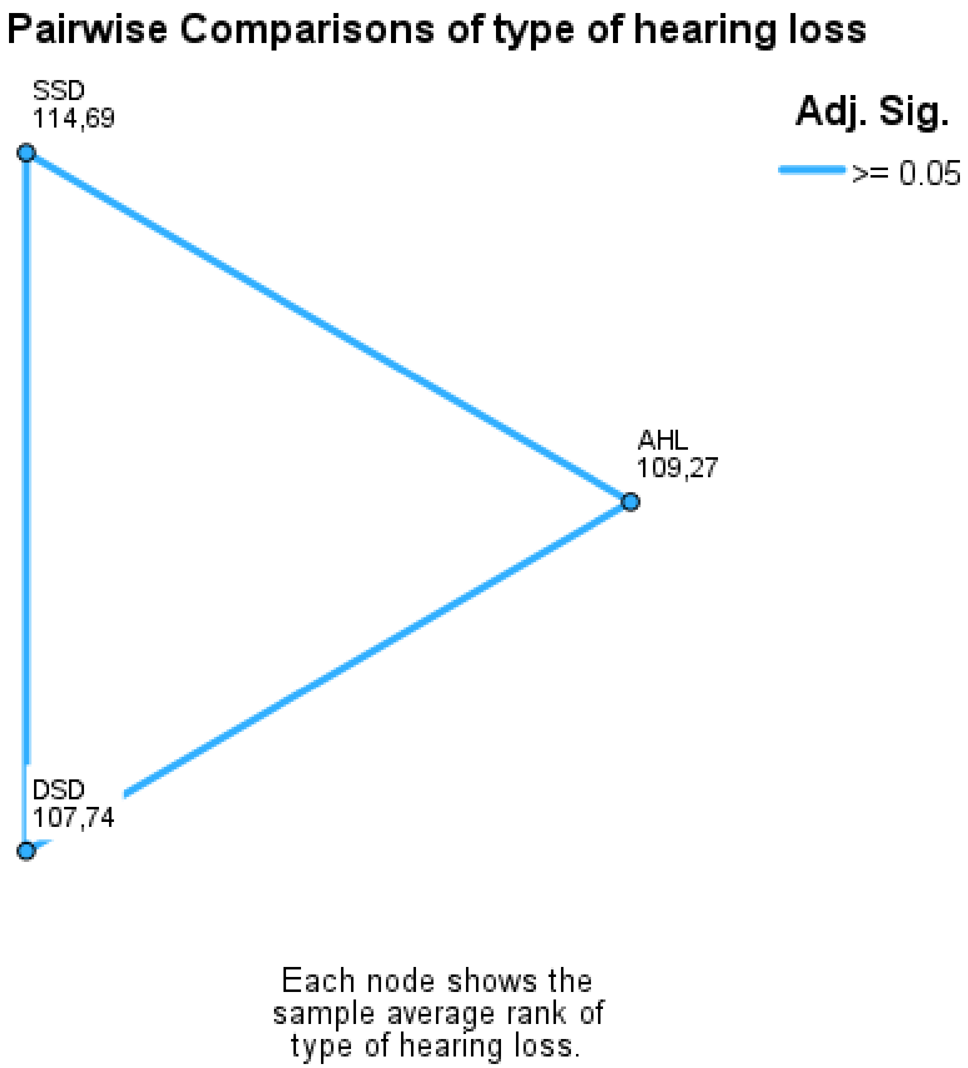

1_ADSL across type of hearing loss

Independent-Samples Kruskal-Wallis Test Summary

Total N	216

Test Statistic	,424[a]
Degree Of Freedom	2
Asymptotic Sig.(2-sided test)	,809

a. The test statistic is adjusted for ties.

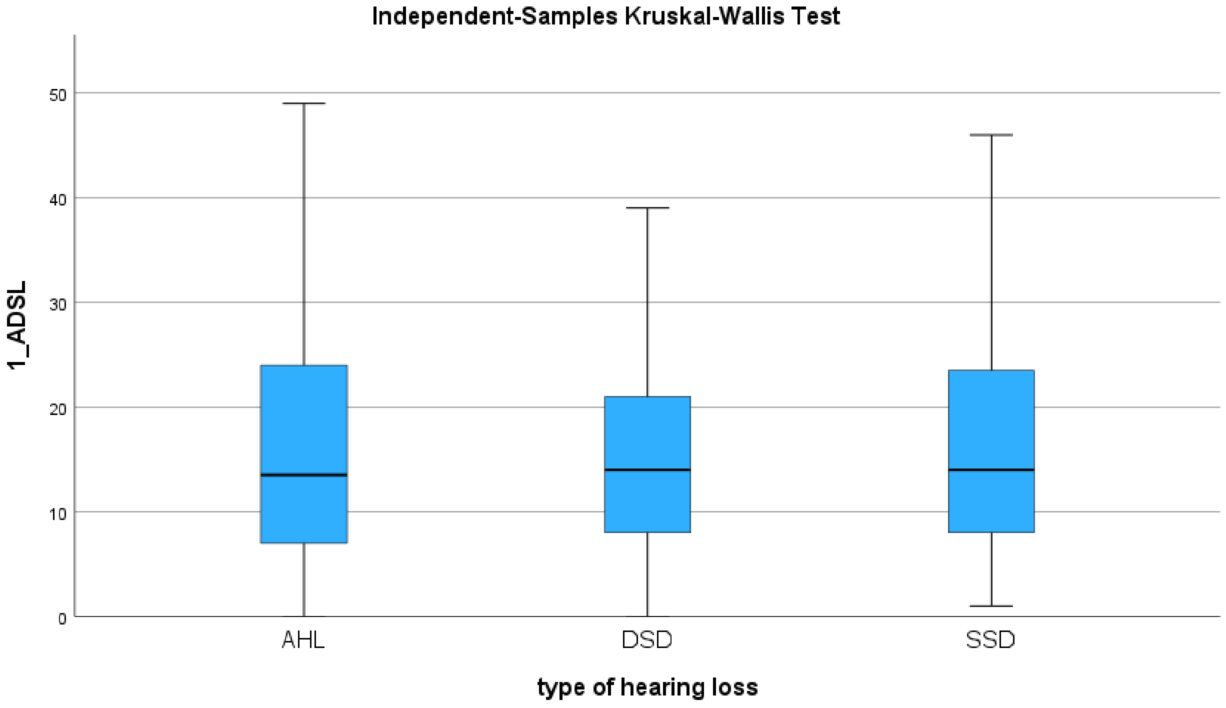

Pairwise Comparisons of type of hearing loss

Sample 1-Sample 2	Test Statistic	Std. Error	Std. Test Statistic	Sig.	Adj. Sig.[a]
AHL-DSD	-4,725	10,056	-,470	,638	1,000
AHL-SSD	-7,932	12,566	-,631	,528	1,000
DSD-SSD	-3,207	11,162	-,287	,774	1,000

Each row tests the null hypothesis that the Sample 1 and Sample 2 distributions are the same.

Asymptotic significances (2-sided tests) are displayed. The significance level is ,050.

a. Significance values have been adjusted by the Bonferroni correction for multiple tests.

Pairwise Comparisons of type of hearing loss

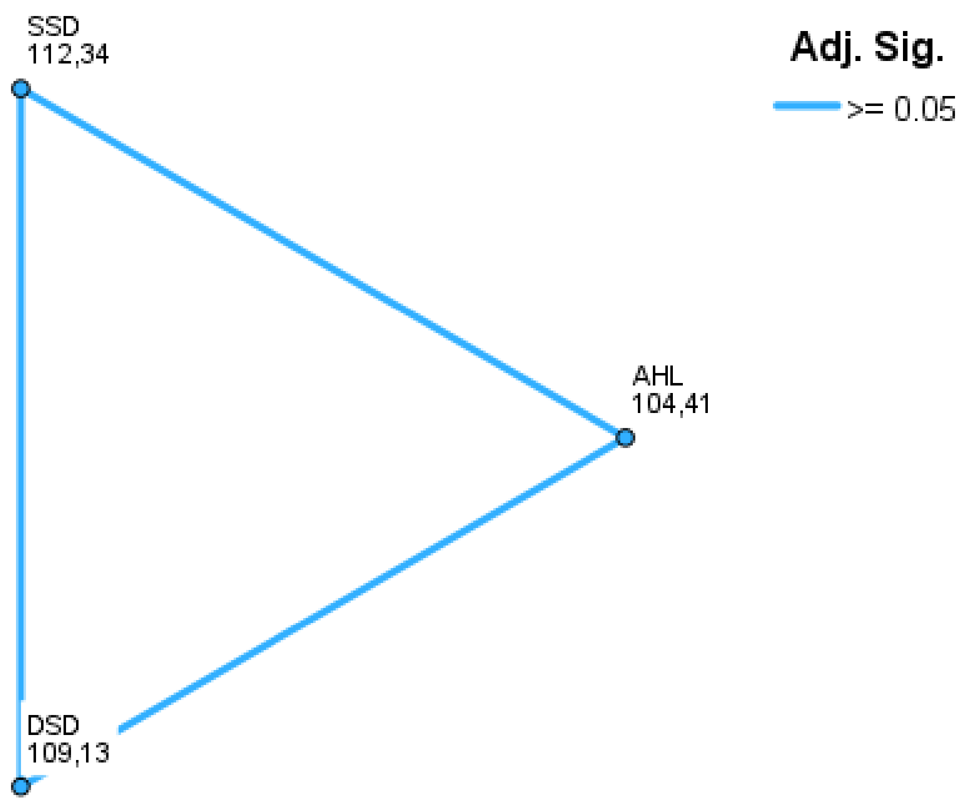

Each node shows the sample average rank of type of hearing loss.

GAD across type of hearing loss

Independent-Samples Kruskal-Wallis Test Summary

Total N	218

Test Statistic	3,079[a]
Degree Of Freedom	2
Asymptotic Sig.(2-sided test)	,214

a. The test statistic is adjusted for ties.

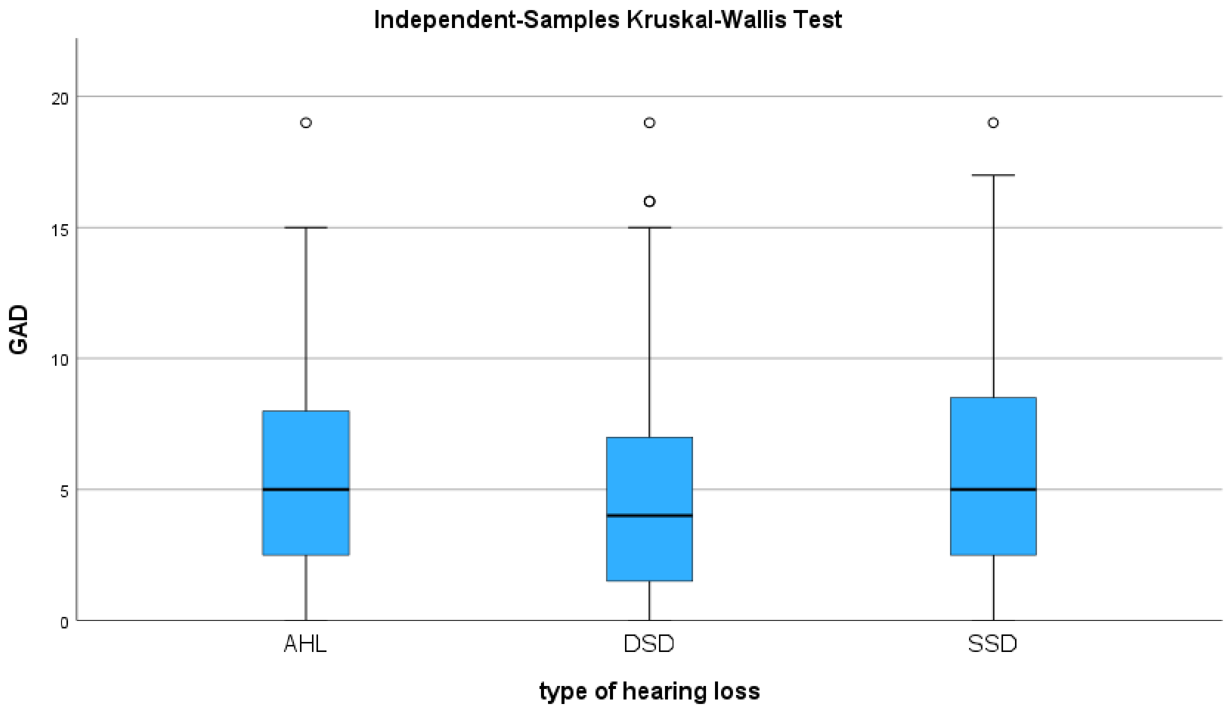

Pairwise Comparisons of type of hearing loss

Sample 1-Sample 2	Test Statistic	Std. Error	Std. Test Statistic	Sig.	Adj. Sig.[a]
DSD-AHL	10,977	10,181	1,078	,281	,843
DSD-SSD	-18,316	11,179	-1,639	,101	,304
AHL-SSD	-7,339	12,739	-,576	,565	1,000

Each row tests the null hypothesis that the Sample 1 and Sample 2 distributions are the same.

Asymptotic significances (2-sided tests) are displayed. The significance level is ,050.

a. Significance values have been adjusted by the Bonferroni correction for multiple tests.

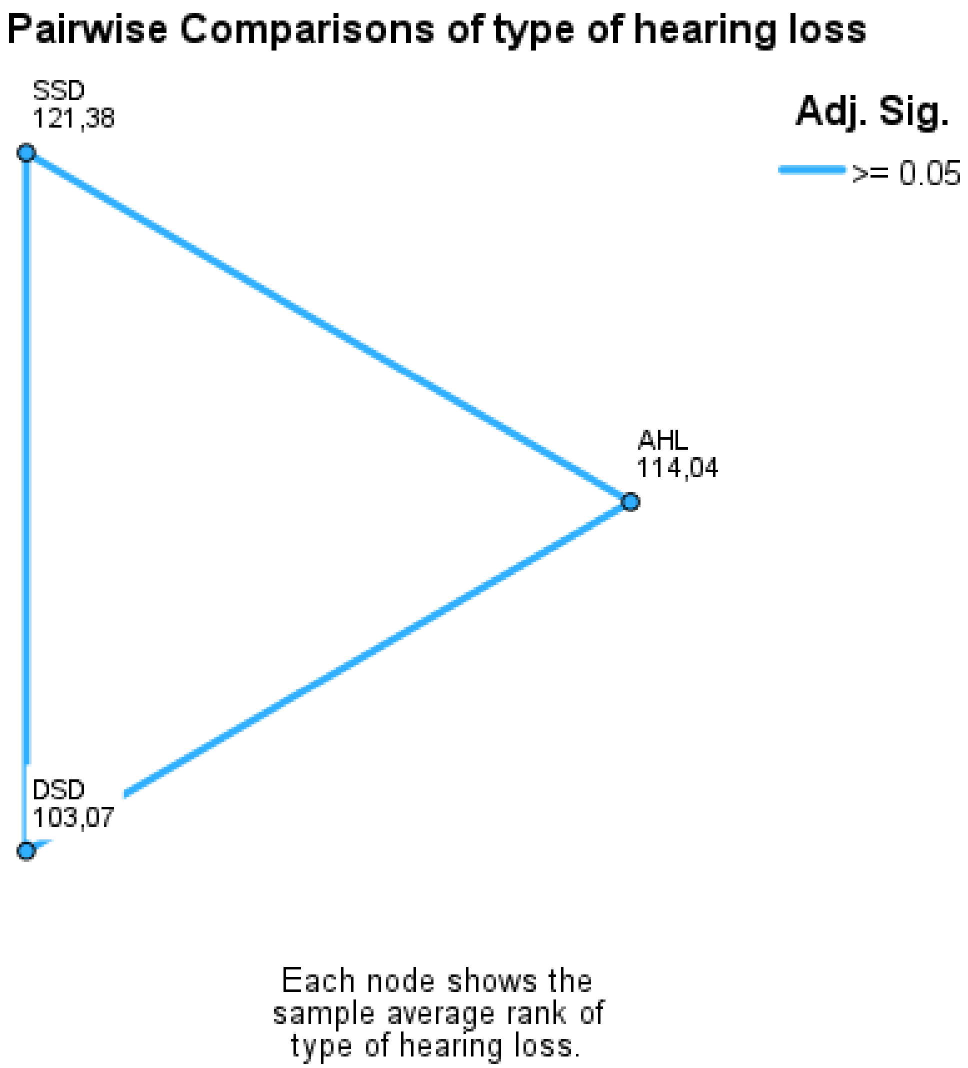

1_GAD across type of hearing loss

Independent-Samples Kruskal-Wallis Test Summary

Total N	219

Test Statistic	,580[a]
Degree Of Freedom	2
Asymptotic Sig.(2-sided test)	,748

a. The test statistic is adjusted for ties.

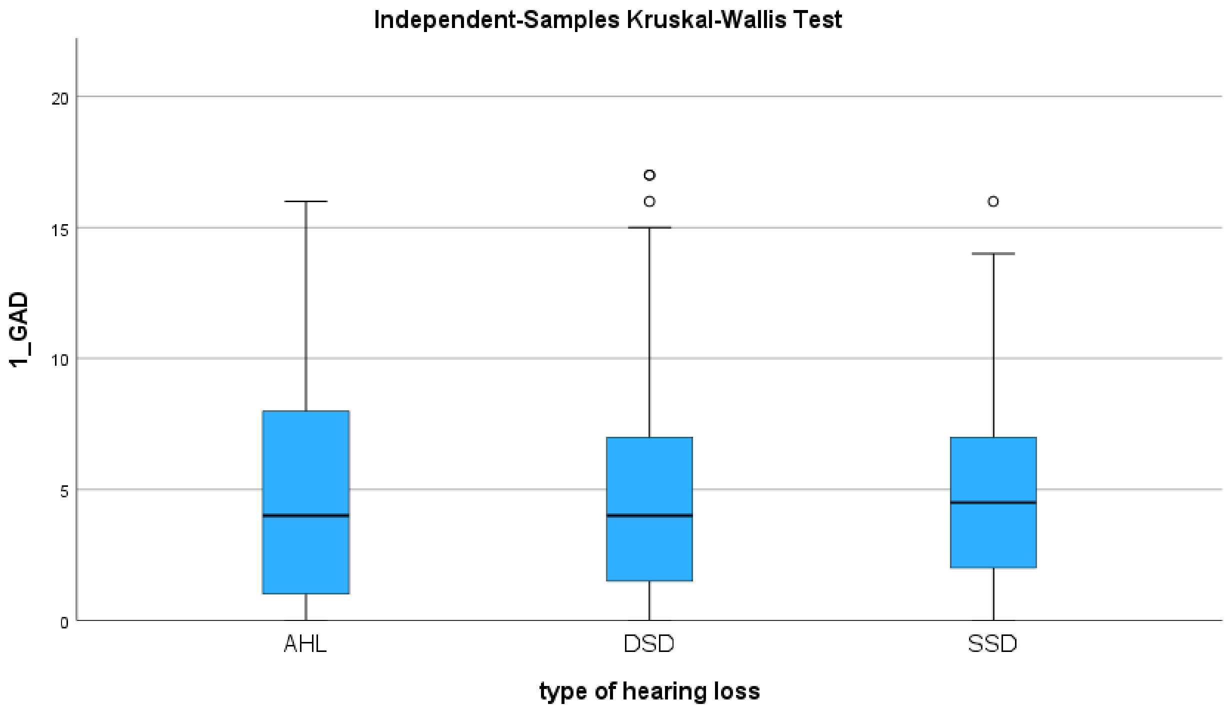

Pairwise Comparisons of type of hearing loss

Sample 1-Sample 2	Test Statistic	Std. Error	Std. Test Statistic	Sig.	Adj. Sig.[a]
DSD-AHL	,323	10,099	,032	,975	1,000
DSD-SSD	-8,340	11,319	-,737	,461	1,000
AHL-SSD	-8,017	12,778	-,627	,530	1,000

Each row tests the null hypothesis that the Sample 1 and Sample 2 distributions are the same.
Asymptotic significances (2-sided tests) are displayed. The significance level is ,050.
a. Significance values have been adjusted by the Bonferroni correction for multiple tests.

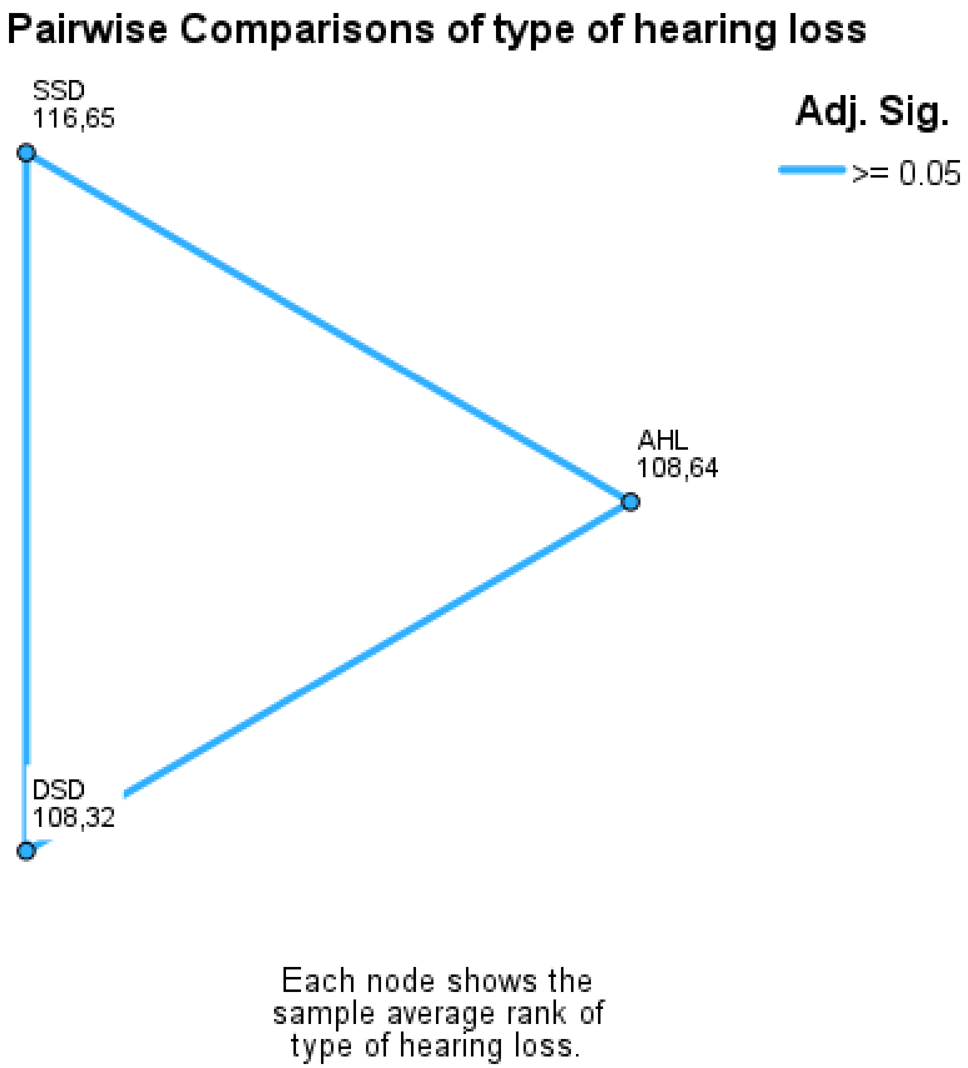

PSQ across type of hearing loss

Independent-Samples Kruskal-Wallis Test Summary

Total N	220

Test Statistic	4,766[a]
Degree Of Freedom	2
Asymptotic Sig.(2-sided test)	,092

a. The test statistic is adjusted for ties.

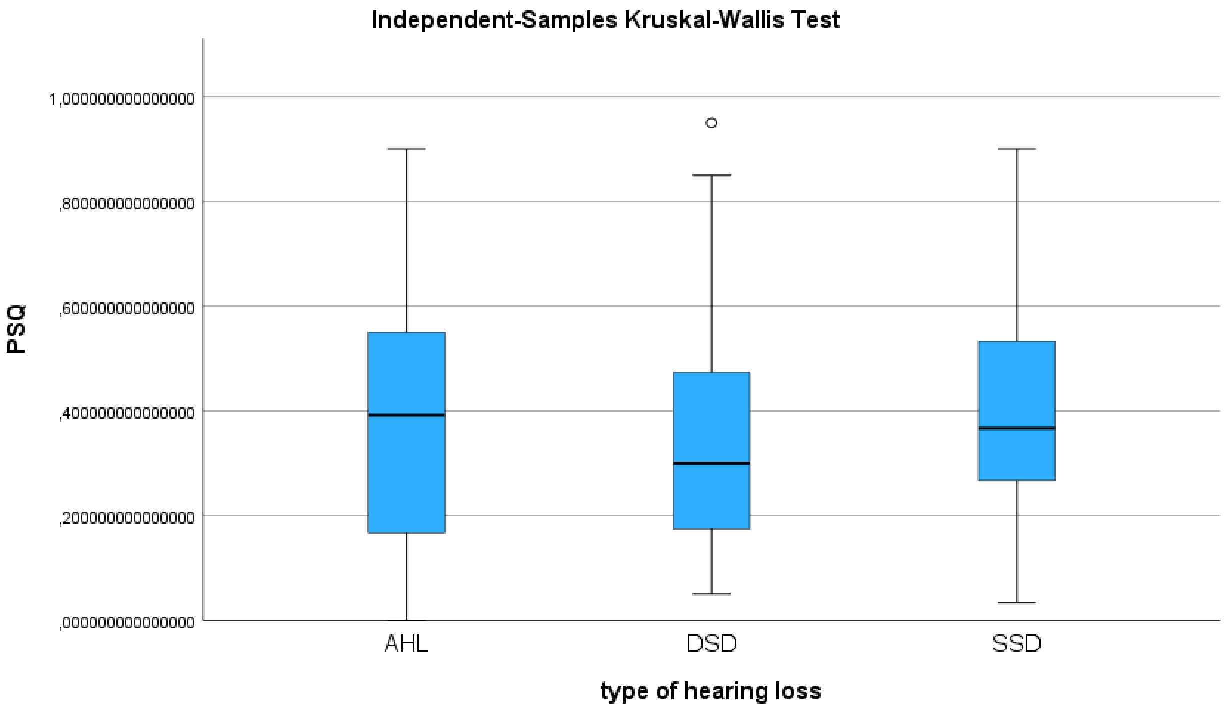

Pairwise Comparisons of type of hearing loss

Sample 1-Sample 2	Test Statistic	Std. Error	Std. Test Statistic	Sig.	Adj. Sig.[a]
DSD-AHL	13,871	10,192	1,361	,174	,521
DSD-SSD	-23,016	11,325	-2,032	,042	,126
AHL-SSD	-9,145	12,808	-,714	,475	1,000

Each row tests the null hypothesis that the Sample 1 and Sample 2 distributions are the same.
Asymptotic significances (2-sided tests) are displayed. The significance level is ,050.
a. Significance values have been adjusted by the Bonferroni correction for multiple tests.

Pairwise Comparisons of type of hearing loss

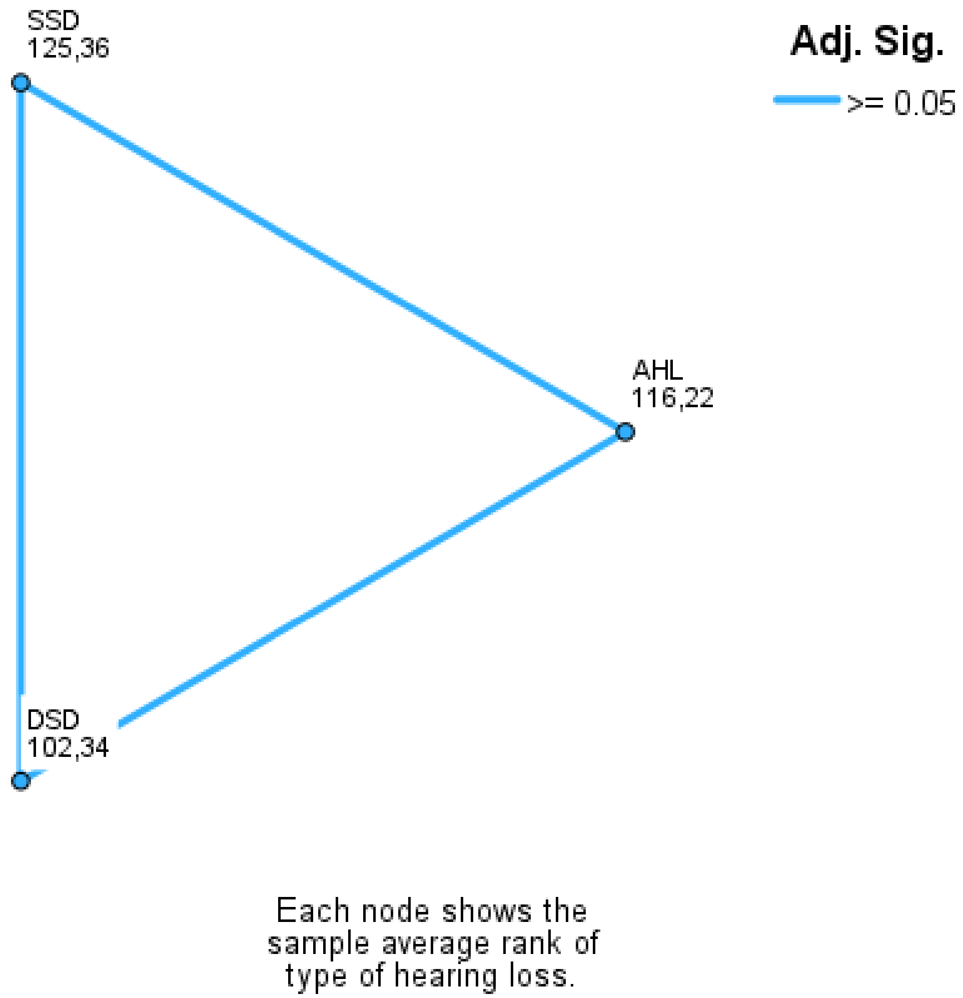

1_PSQ across type of hearing loss

Independent-Samples Kruskal-Wallis Test Summary

Total N	219

Test Statistic	,283[a]
Degree Of Freedom	2
Asymptotic Sig.(2-sided test)	,868

a. The test statistic is adjusted for ties.

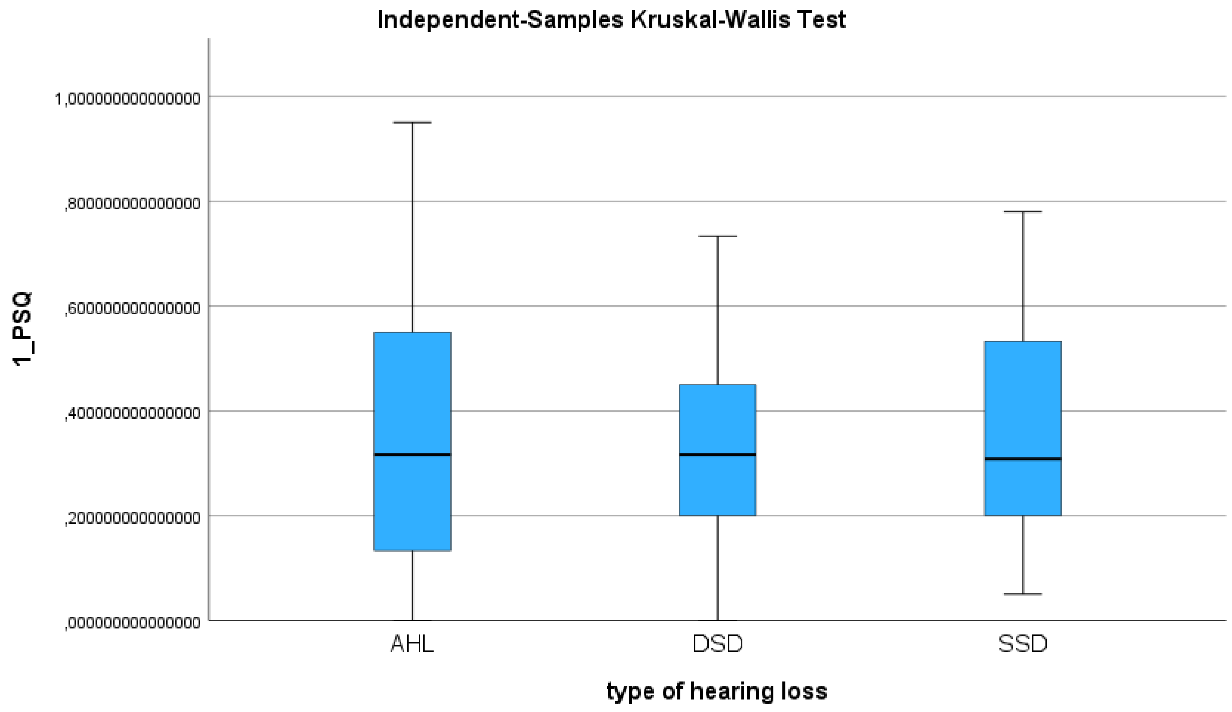

Pairwise Comparisons of type of hearing loss

Sample 1-Sample 2	Test Statistic	Std. Error	Std. Test Statistic	Sig.	Adj. Sig.[a]
DSD-AHL	2,947	10,145	,290	,771	1,000
DSD-SSD	-5,812	11,370	-,511	,609	1,000
AHL-SSD	-2,865	12,836	-,223	,823	1,000

Each row tests the null hypothesis that the Sample 1 and Sample 2 distributions are the same.

Asymptotic significances (2-sided tests) are displayed. The significance level is ,050.

a. Significance values have been adjusted by the Bonferroni correction for multiple tests.

Pairwise Comparisons of type of hearing loss

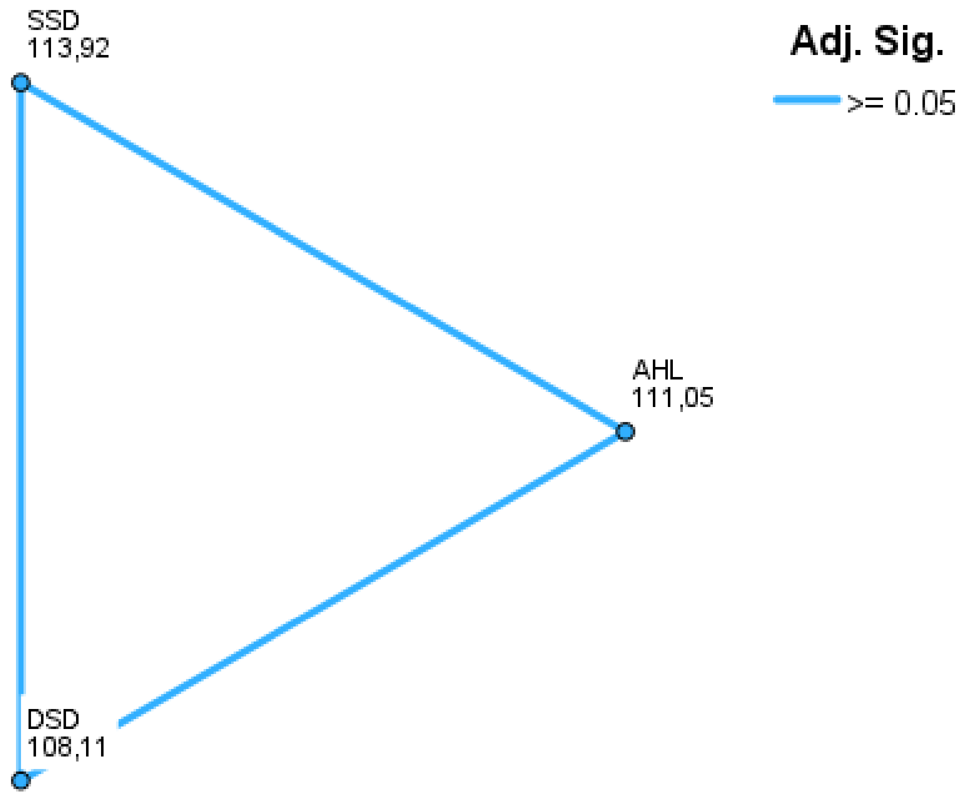

Each node shows the sample average rank of type of hearing loss.

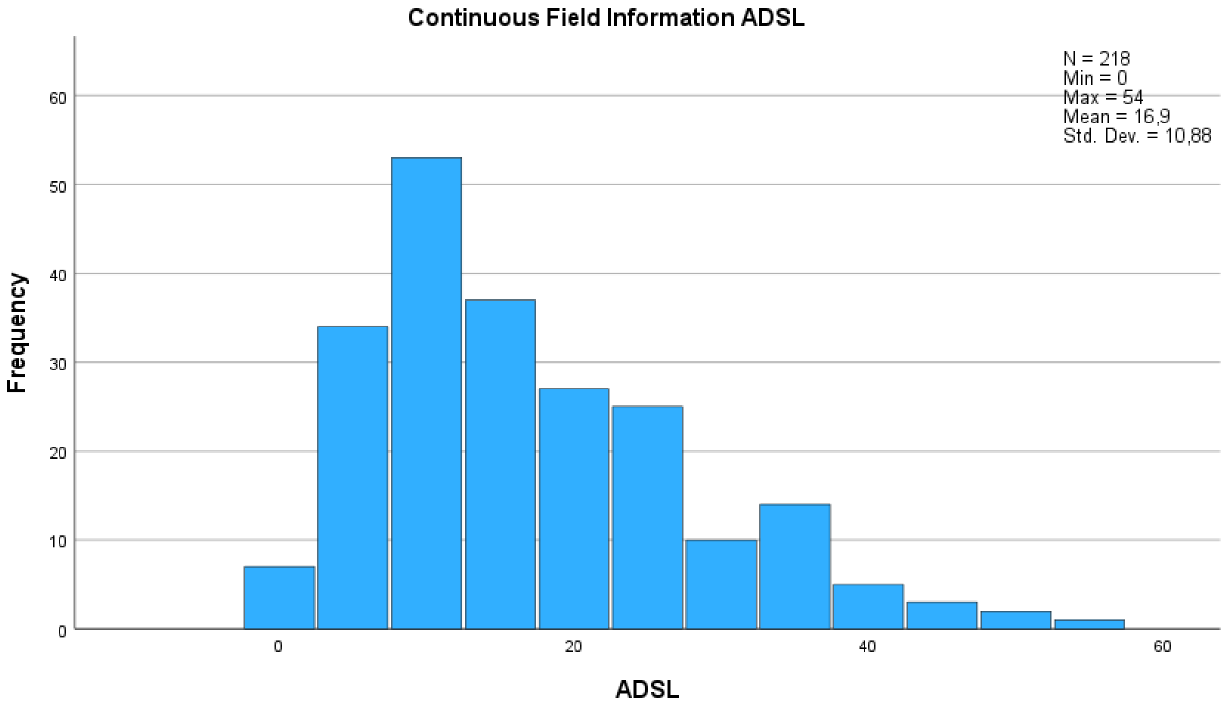
Continuous Field Information ADSL
N = 218
Min = 0
Max = 54
Mean = 16,9
Std. Dev. = 10,88
Frequency
0
10
20
30
40
50
60
0
20
40
60
ADSL

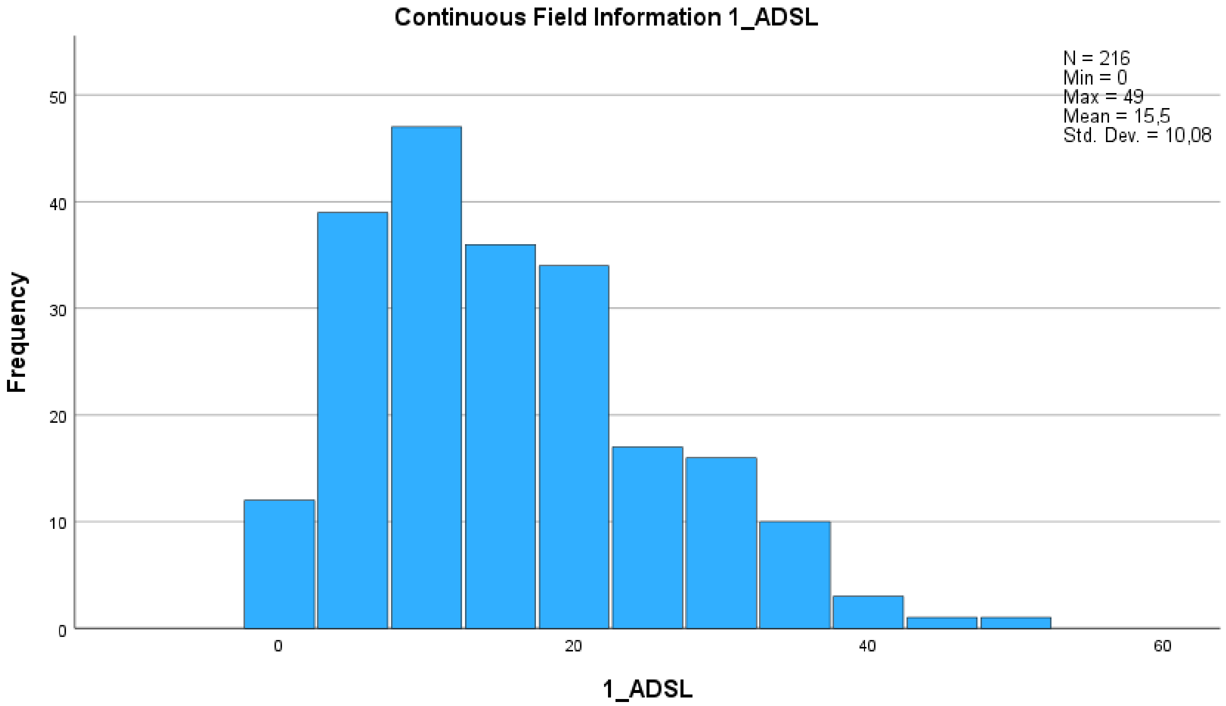
Continuous Field Information 1_ADSL
N = 216
Min = 0
Max = 49
Mean = 15,5
Std. Dev. = 10,08
Frequency
0
10
20
30
40
50
0
20
40
60
1_ADSL

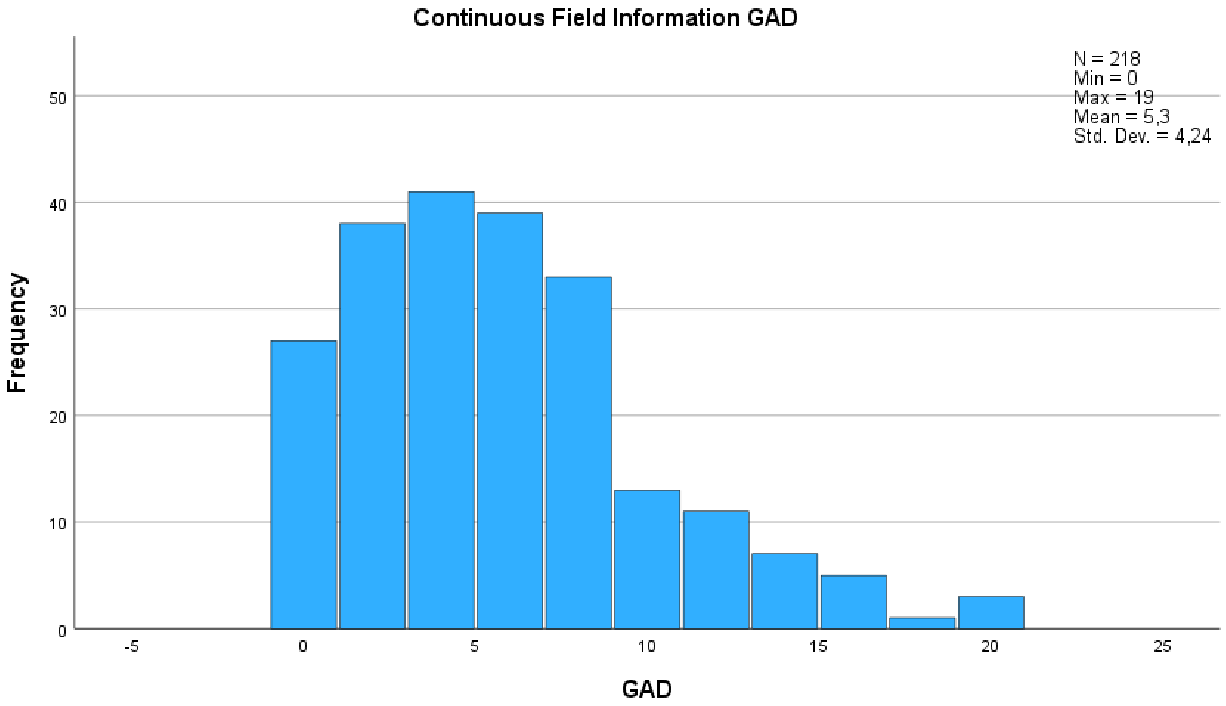
Continuous Field Information GAD
N = 218
Min = 0
Max = 19
Mean = 5,3
Std. Dev. = 4,24
Frequency
GAD

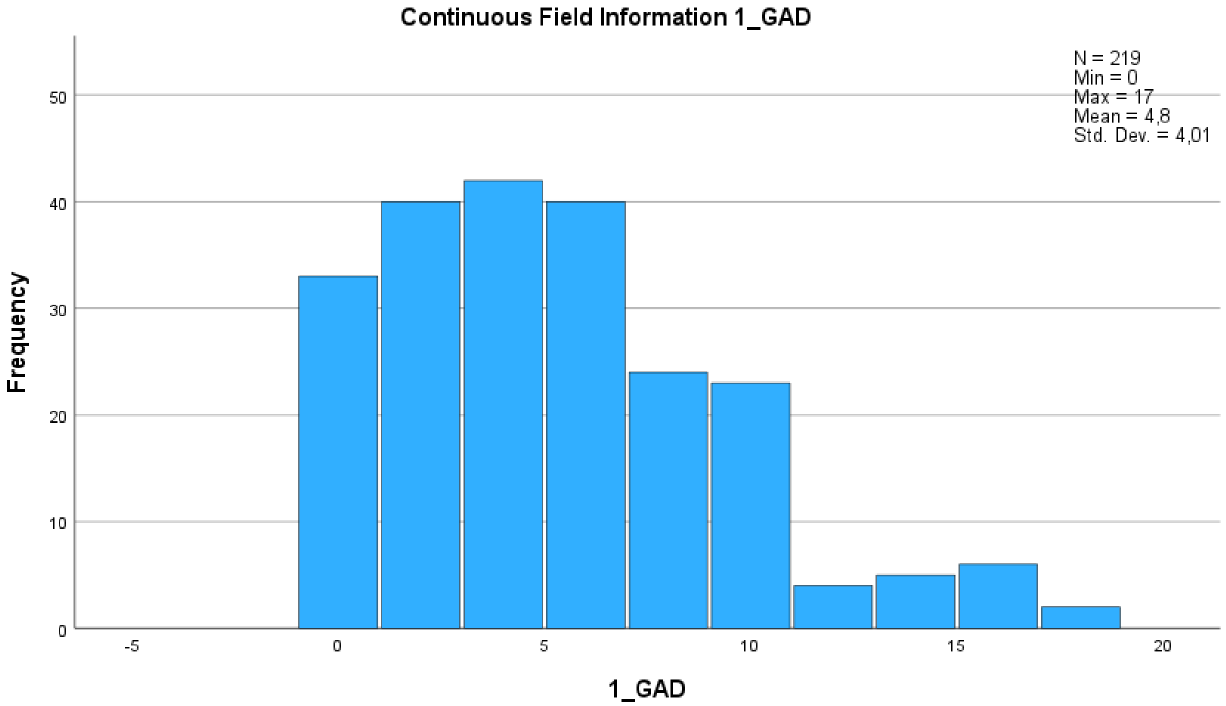
Continuous Field Information 1_GAD
N = 219
Min = 0
Max = 17
Mean = 4,8
Std. Dev. = 4,01
Frequency
1_GAD

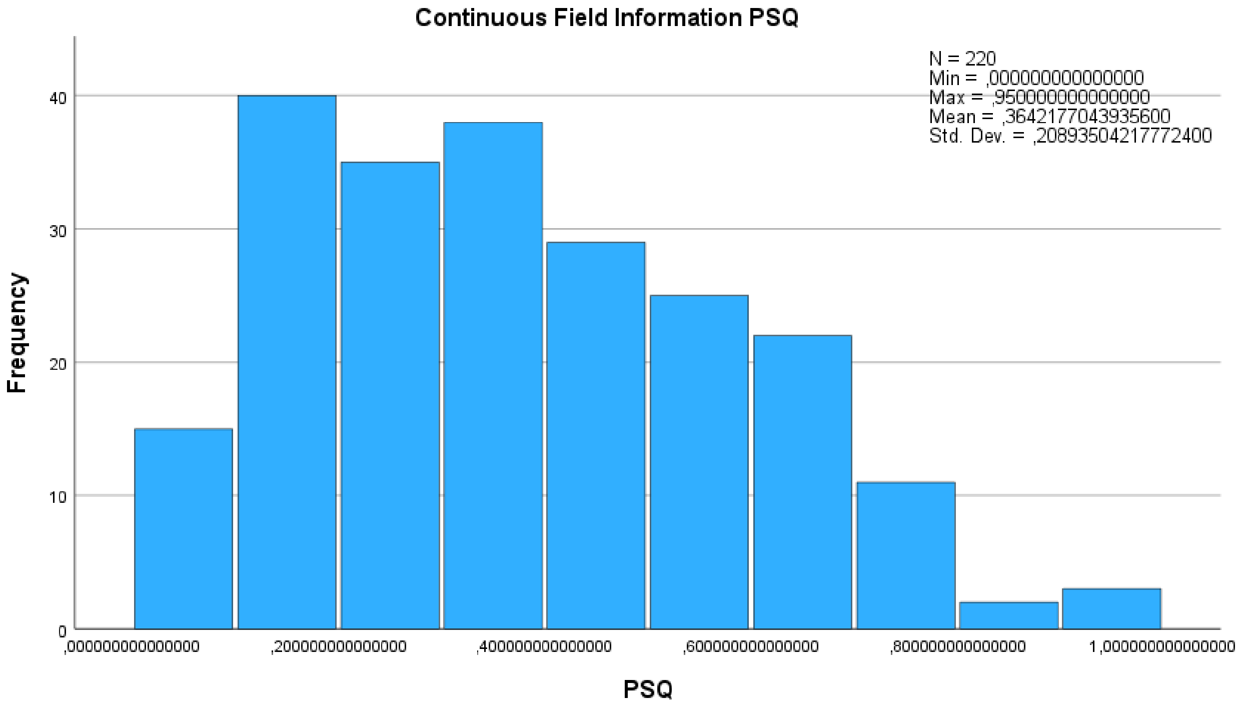
Continuous Field Information PSQ
N = 220
Min = ,000000000000000
Max = ,950000000000000
Mean = ,3642177043935600
Std. Dev. = ,2089350421772400
Frequency
0
10
20
30
40
,000000000000000
,200000000000000
,400000000000000
,600000000000000
,800000000000000
1,000000000000000
PSQ

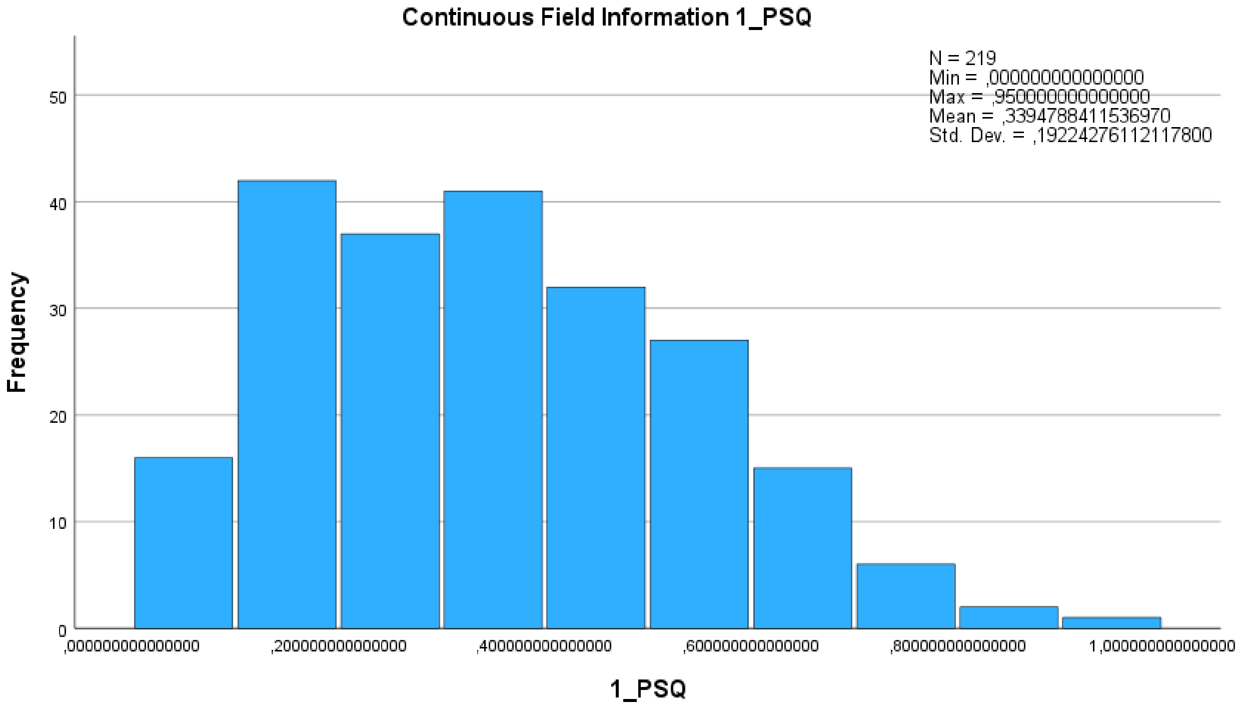
Continuous Field Information 1_PSQ
N = 219
Min = ,000000000000000
Max = ,950000000000000
Mean = ,3394788411536970
Std. Dev. = ,1922427611211780 0
Frequency
0
10
20
30
40
50
,000000000000000
,200000000000000
,400000000000000
,600000000000000
,800000000000000
1,000000000000000
1_PSQ

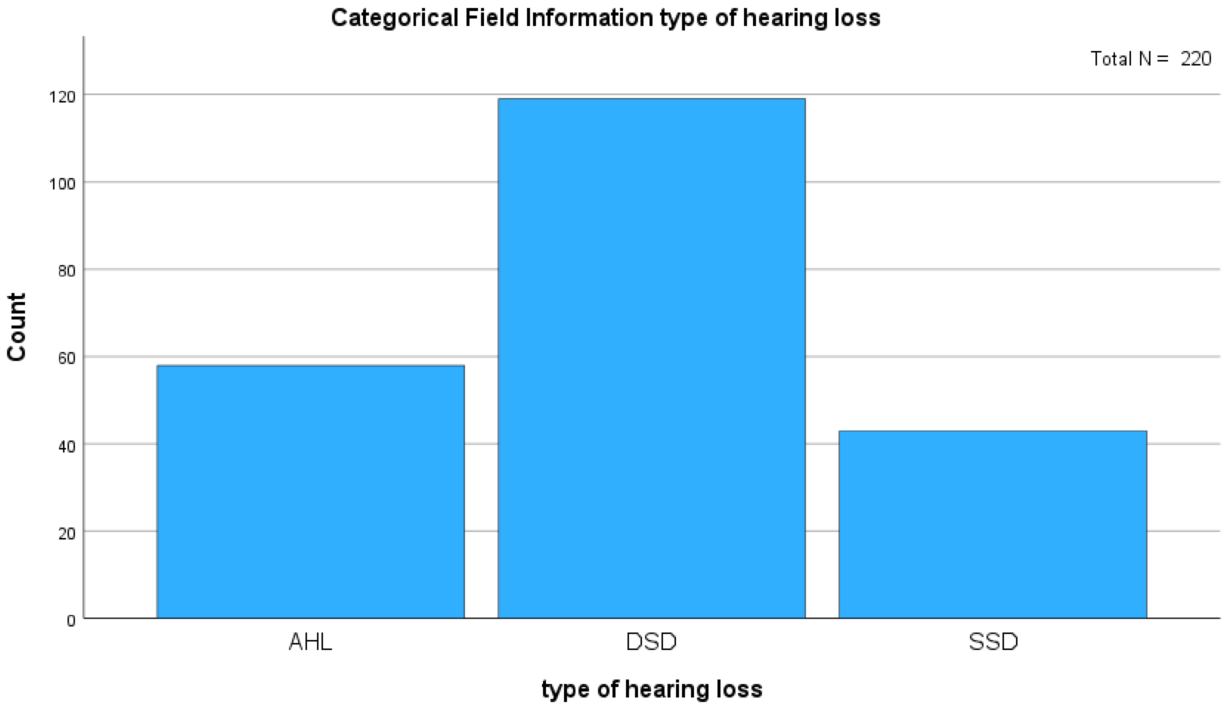

Categorical Field Information type of hearing loss
Total N = 220
Count
120
100
80
60
40
20
0
AHL
DSD
SSD
type of hearing loss
