# Supplementary material for: Changes in Speech Intelligibility, Health-Related Quality of Life, Depressive Symptoms, Anxiety, Perceived Stress, and Tinnitus-Induced Distress, in a Cohort of 227 Adults One Year After Cochlear Implantation: A Decade of Experience from a Single Tertiary Center
Source: J Clin Med. 2025 Nov 17;14(22):8143. doi: 10.3390/jcm14228143 (PMC12653197; doi:10.3390/jcm14228143)
Supplement: Supplementary file 1 [file jcm-14-08143-s001.zip › S3.pdf]

S3: Kruskal-Wallis Test: the comparison between the pre- and post-scores of TQ for AHL, DSD, and SSD groups.

Hypothesis Test Summary

	Null Hypothesis	Test	Sig.[a,b]	Decision
1	The distribution of TQEC is the same across categories of type of hearing loss.	Independent-Samples Kruskal-Wallis Test	,065	Retain the null hypothesis.
2	The distribution of TQI is the same across categories of type of hearing loss.	Independent-Samples Kruskal-Wallis Test	,023	Reject the null hypothesis.
3	The distribution of TQA is the same across categories of type of hearing loss.	Independent-Samples Kruskal-Wallis Test	,296	Retain the null hypothesis.
4	The distribution of TQSI is the same across categories of type of hearing loss.	Independent-Samples Kruskal-Wallis Test	,709	Retain the null hypothesis.
5	The distribution of TQSO is the same across categories of type of hearing loss.	Independent-Samples Kruskal-Wallis Test	,177	Retain the null hypothesis.
6	The distribution of TQTotal is the same across categories of type of hearing loss.	Independent-Samples Kruskal-Wallis Test	,070	Retain the null hypothesis.
7	The distribution of 1_TQEC is the same across categories of type of hearing loss.	Independent-Samples Kruskal-Wallis Test	,665	Retain the null hypothesis.
8	The distribution of 1_TQI is the same across categories of type of hearing loss.	Independent-Samples Kruskal-Wallis Test	,409	Retain the null hypothesis.
9	The distribution of 1_TQA is the same across categories of type of hearing loss.	Independent-Samples Kruskal-Wallis Test	,479	Retain the null hypothesis.
10	The distribution of 1_TQSI is the same across categories of type of hearing loss.	Independent-Samples Kruskal-Wallis Test	,949	Retain the null hypothesis.
11	The distribution of 1_TQSO is the same across categories of type of hearing loss.	Independent-Samples Kruskal-Wallis Test	,224	Retain the null hypothesis.

12	The distribution of 1_TQTotal is the same across categories of type of hearing loss.	Independent-Samples Kruskal-Wallis Test	,699	Retain the null hypothesis.

a. The significance level is ,050.
b. Asymptotic significance is displayed.

Independent-Samples Kruskal-Wallis Test

TQEC across type of hearing loss

Independent-Samples Kruskal-Wallis Test Summary

Total N	220
Test Statistic	5,465[a]
Degree Of Freedom	2
Asymptotic Sig.(2-sided test)	,065

a. The test statistic is adjusted for ties.

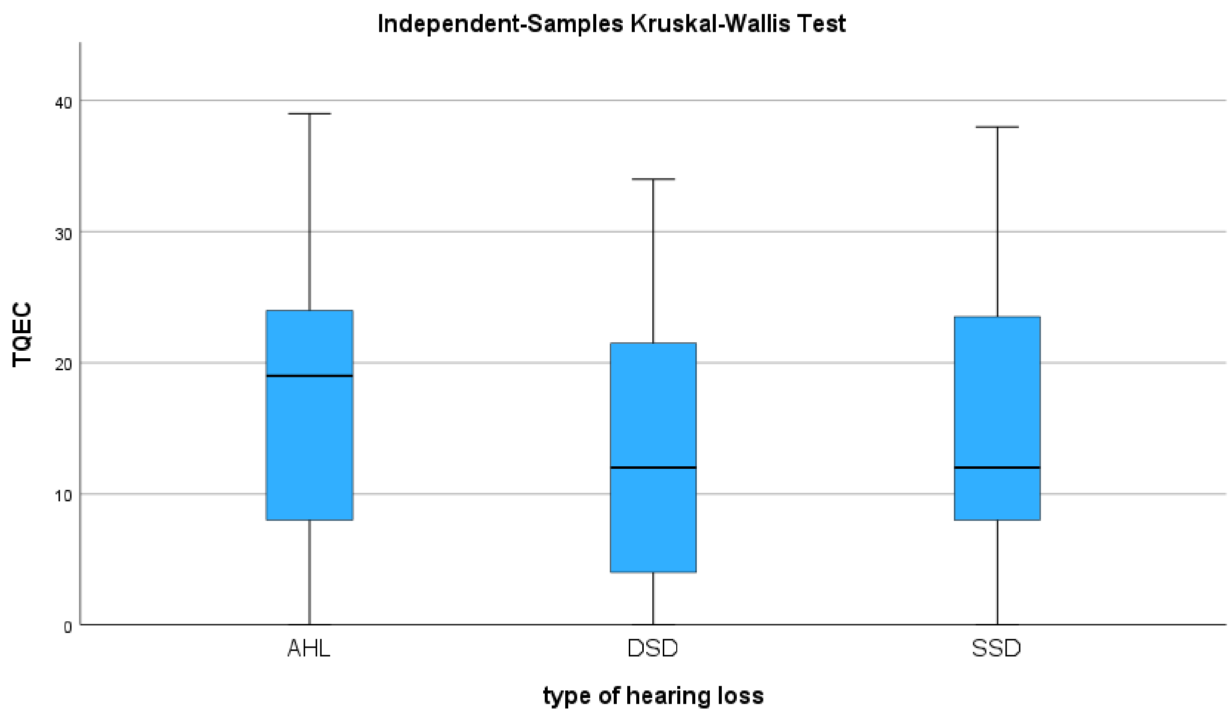

Pairwise Comparisons of type of hearing loss

Sample 1-Sample 2	Test Statistic	Std. Error	Std. Test Statistic	Sig.	Adj. Sig.[a]
DSD-SSD	-12,904	11,314	-1,140	,254	,762
DSD-AHL	23,280	10,183	2,286	,022	,067
SSD-AHL	10,377	12,797	,811	,417	1,000

Each row tests the null hypothesis that the Sample 1 and Sample 2 distributions are the same.

Asymptotic significances (2-sided tests) are displayed. The significance level is ,050.

a. Significance values have been adjusted by the Bonferroni correction for multiple tests.

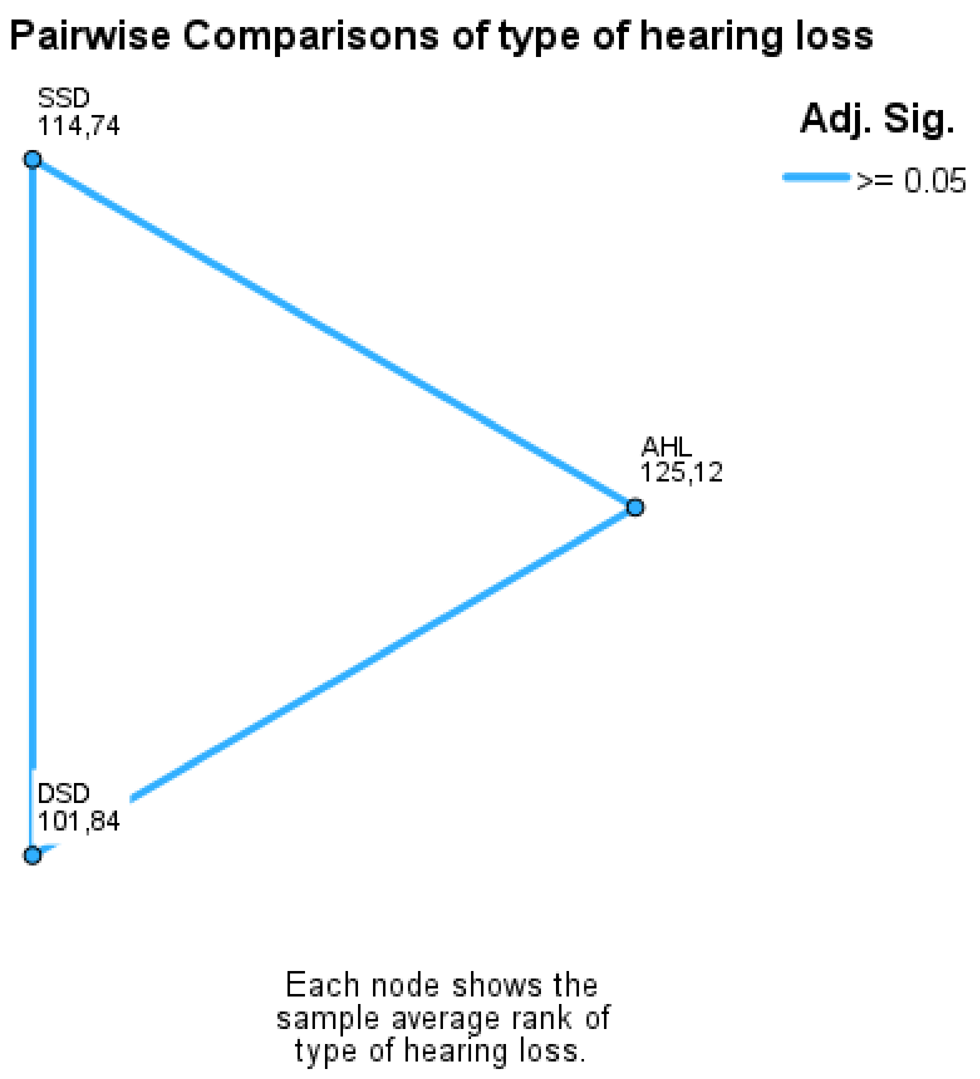

TQI across type of hearing loss

Independent-Samples Kruskal-Wallis Test Summary

Total N	220

Test Statistic	7,557[a]
Degree Of Freedom	2
Asymptotic Sig.(2-sided test)	,023

a. The test statistic is adjusted for ties.

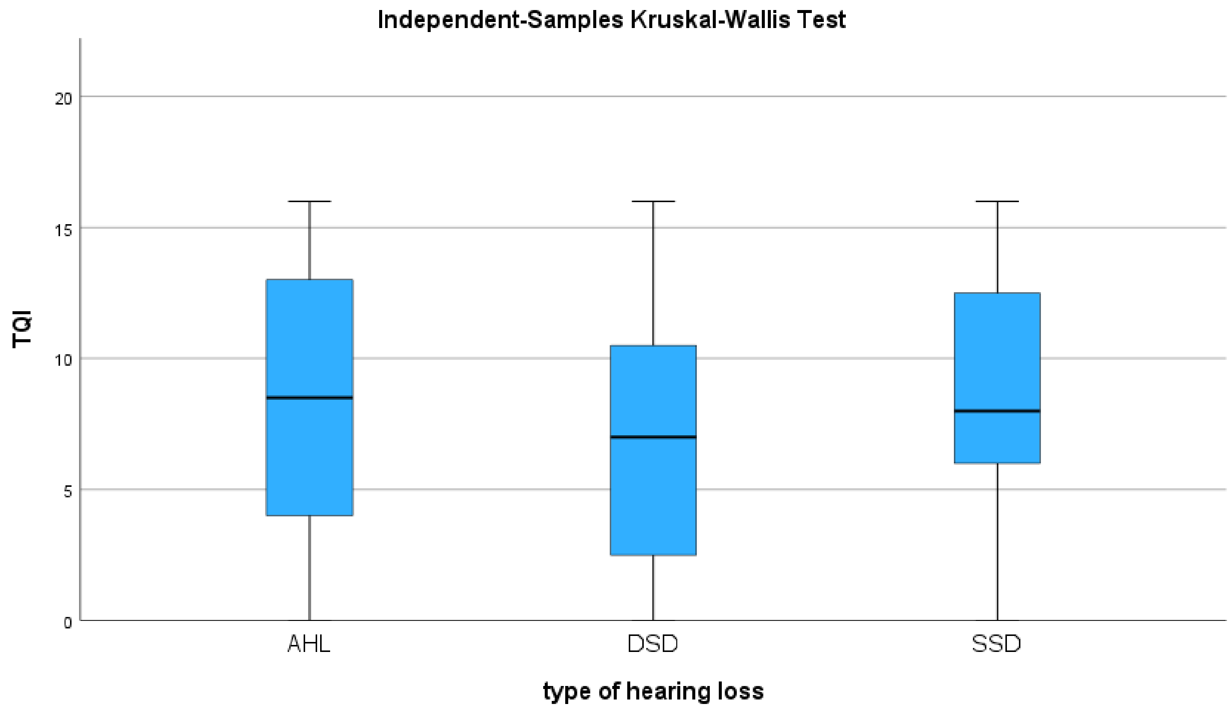

Pairwise Comparisons of type of hearing loss

Sample 1-Sample 2	Test Statistic	Std. Error	Std. Test Statistic	Sig.	Adj. Sig.[a]
DSD-AHL	21,511	10,167	2,116	,034	,103
DSD-SSD	-25,996	11,297	-2,301	,021	,064
AHL-SSD	-4,486	12,777	-,351	,726	1,000

Each row tests the null hypothesis that the Sample 1 and Sample 2 distributions are the same.
Asymptotic significances (2-sided tests) are displayed. The significance level is ,050.
a. Significance values have been adjusted by the Bonferroni correction for multiple tests.

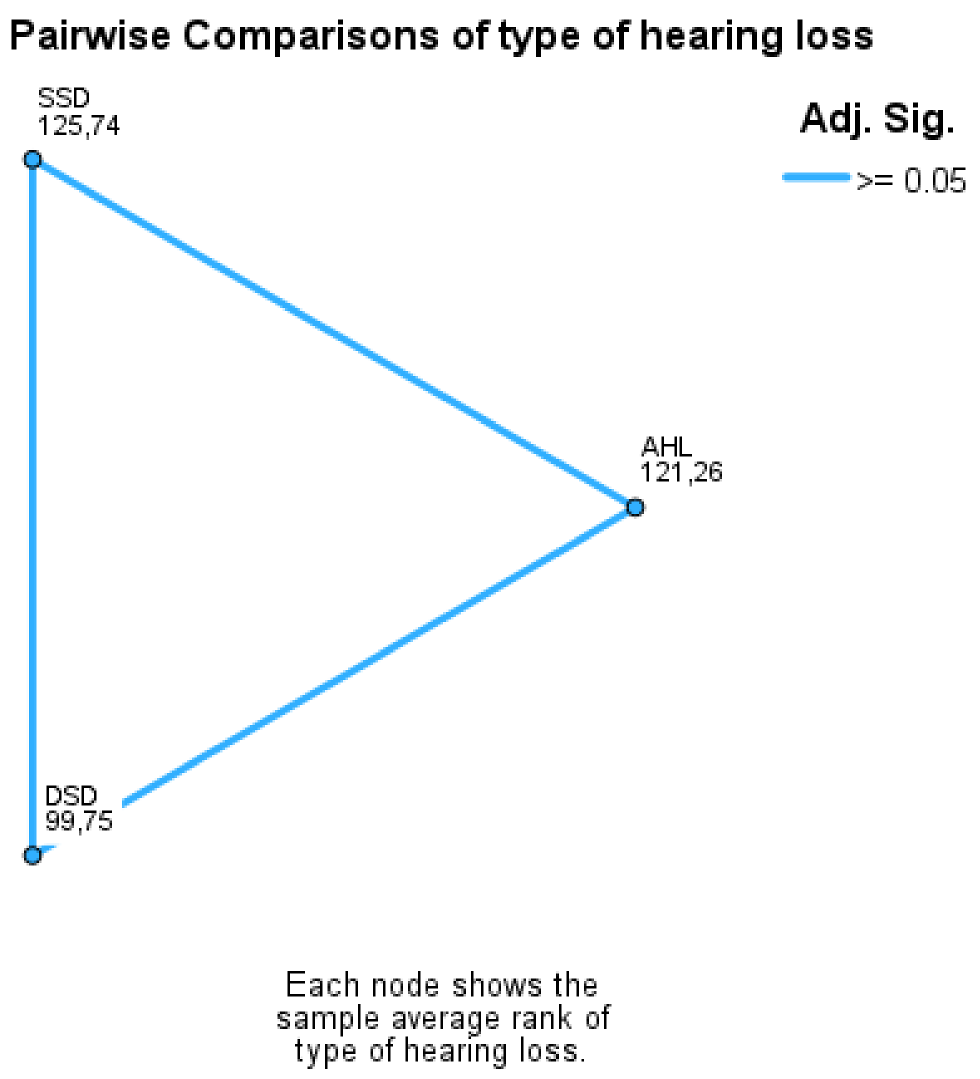

TQA across type of hearing loss

Independent-Samples Kruskal-Wallis Test Summary

Total N	220

Test Statistic	2,433[a]
Degree Of Freedom	2
Asymptotic Sig.(2-sided test)	,296

a. The test statistic is adjusted for ties.

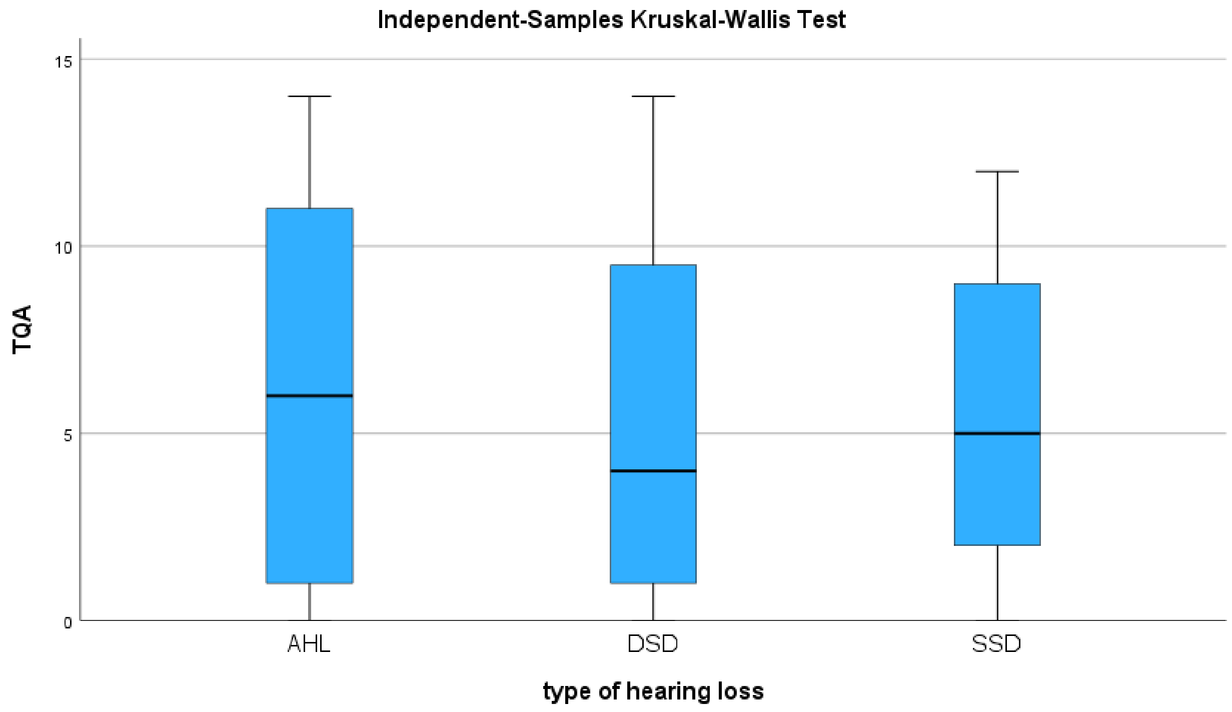

Pairwise Comparisons of type of hearing loss

Sample 1-Sample 2	Test Statistic	Std. Error	Std. Test Statistic	Sig.	Adj. Sig.[a]
DSD-SSD	-4,744	11,257	-,421	,673	1,000
DSD-AHL	15,799	10,131	1,559	,119	,357
SSD-AHL	11,056	12,732	,868	,385	1,000

Each row tests the null hypothesis that the Sample 1 and Sample 2 distributions are the same.

Asymptotic significances (2-sided tests) are displayed. The significance level is ,050.

a. Significance values have been adjusted by the Bonferroni correction for multiple tests.

Pairwise Comparisons of type of hearing loss

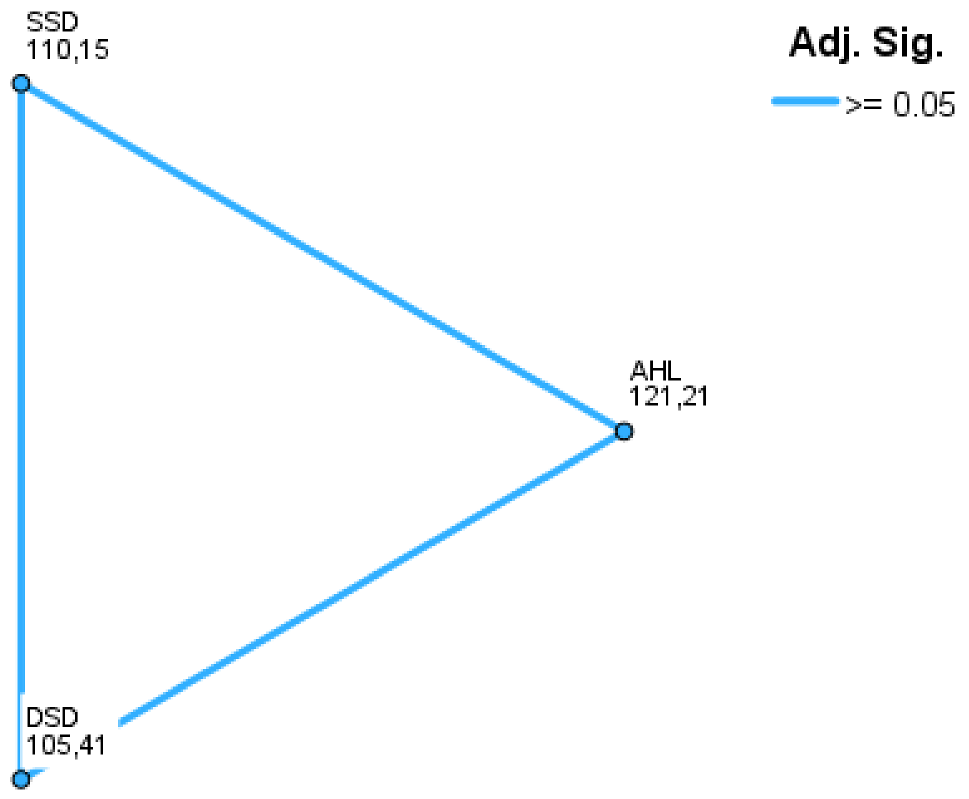

Each node shows the sample average rank of type of hearing loss.

TQSI across type of hearing loss

Independent-Samples Kruskal-Wallis Test Summary

Total N	220

Test Statistic	,687[a]
Degree Of Freedom	2
Asymptotic Sig.(2-sided test)	,709

a. The test statistic is adjusted for ties.

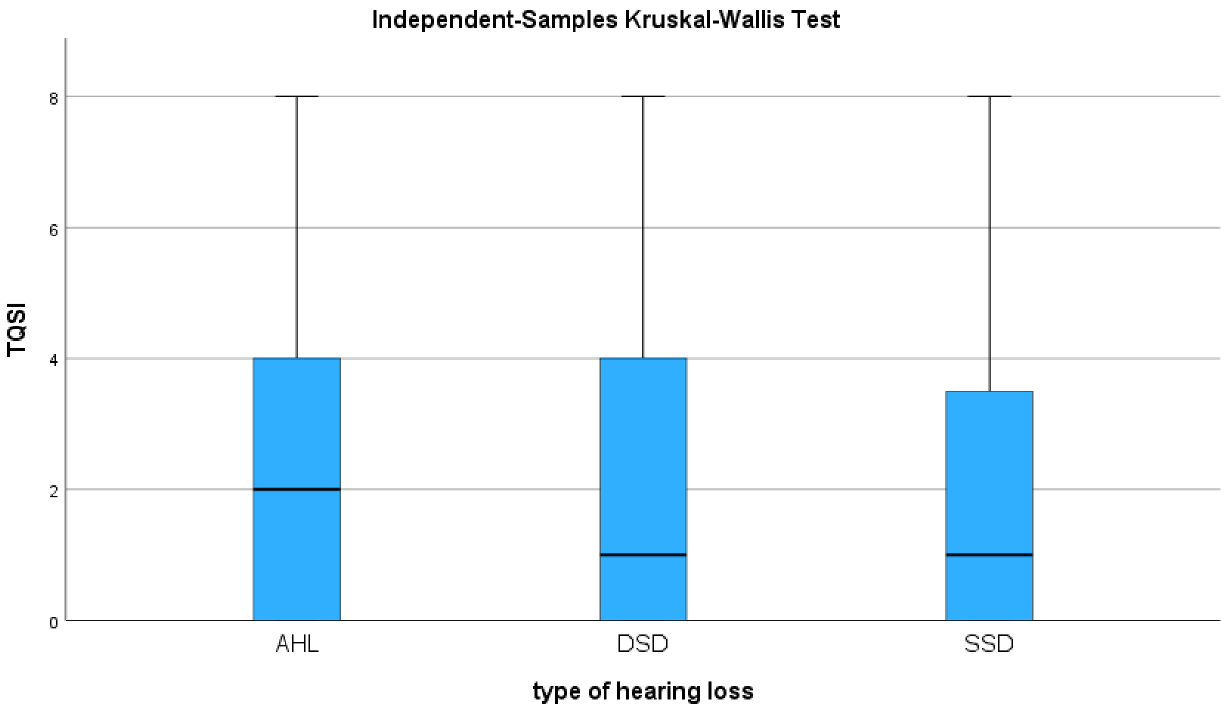

Pairwise Comparisons of type of hearing loss

Sample 1-Sample 2	Test Statistic	Std. Error	Std. Test Statistic	Sig.	Adj. Sig.[a]
DSD-SSD	-1,317	10,952	-,120	,904	1,000
DSD-AHL	8,076	9,857	,819	,413	1,000
SSD-AHL	6,759	12,387	,546	,585	1,000

Each row tests the null hypothesis that the Sample 1 and Sample 2 distributions are the same.
Asymptotic significances (2-sided tests) are displayed. The significance level is ,050.
a. Significance values have been adjusted by the Bonferroni correction for multiple tests.

Pairwise Comparisons of type of hearing loss

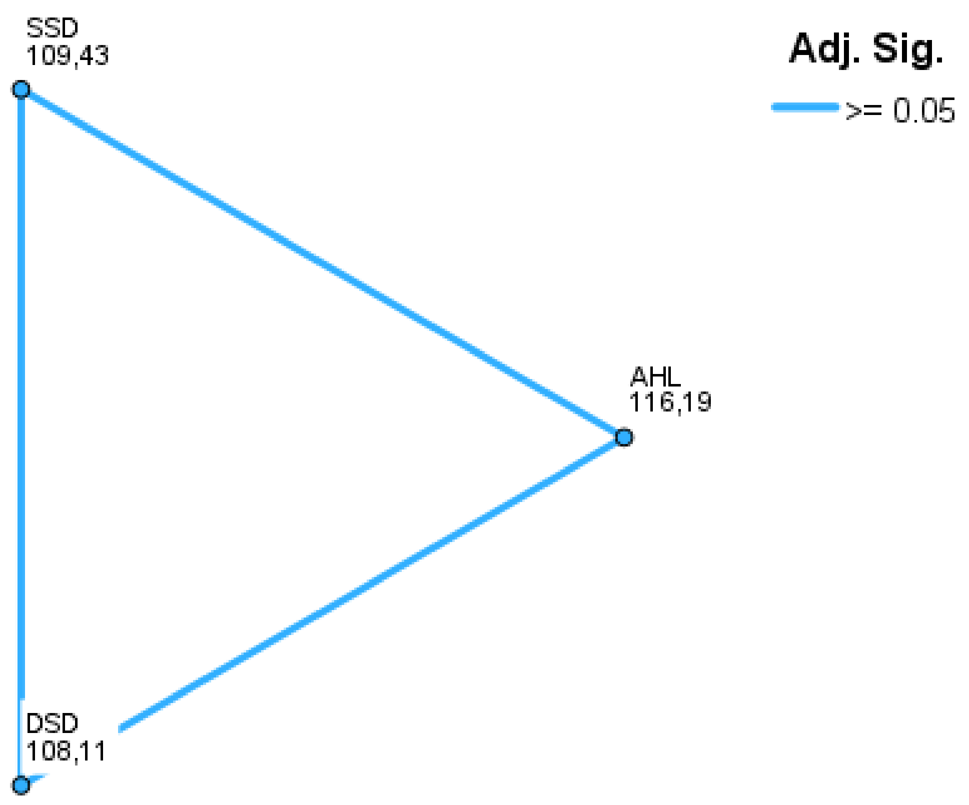

Each node shows the sample average rank of type of hearing loss.

TQSO across type of hearing loss

Independent-Samples Kruskal-Wallis Test Summary

Total N	220

Test Statistic	3,463[a]
Degree Of Freedom	2
Asymptotic Sig.(2-sided test)	,177

a. The test statistic is adjusted for ties.

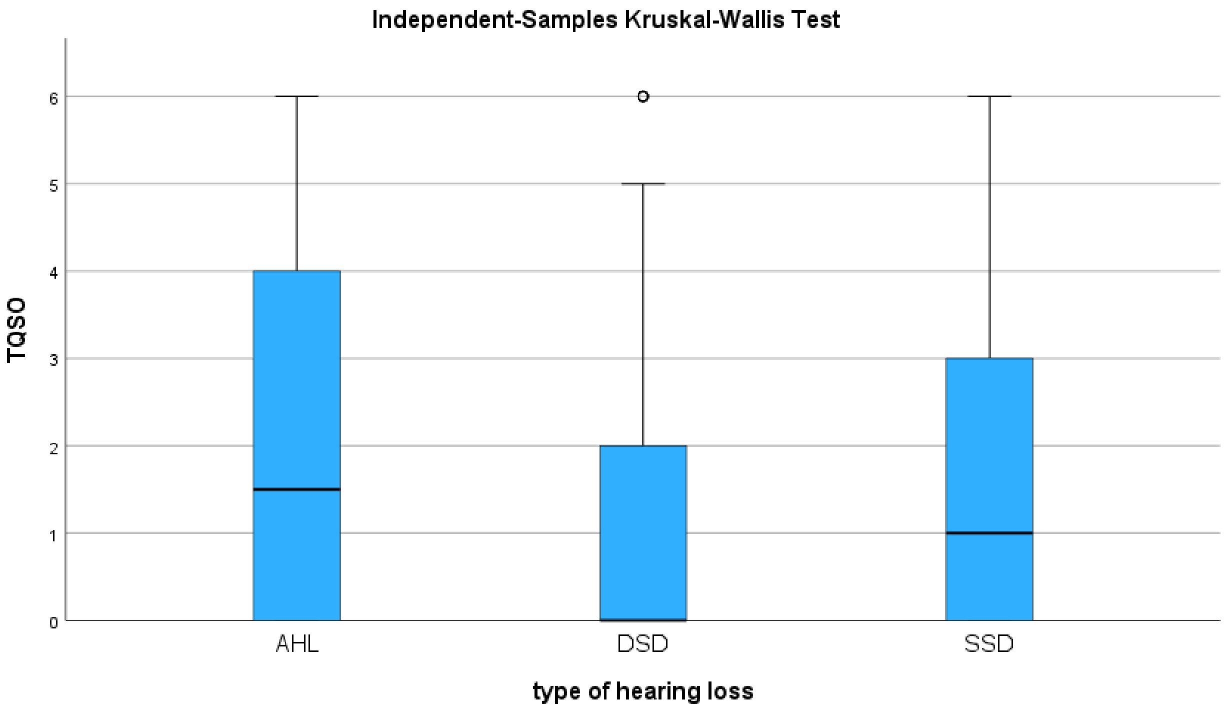

Pairwise Comparisons of type of hearing loss

Sample 1-Sample 2	Test Statistic	Std. Error	Std. Test Statistic	Sig.	Adj. Sig.[a]
DSD-SSD	-3,808	10,659	-,357	,721	1,000
DSD-AHL	17,752	9,594	1,850	,064	,193
SSD-AHL	13,944	12,056	1,157	,247	,742

Each row tests the null hypothesis that the Sample 1 and Sample 2 distributions are the same.
Asymptotic significances (2-sided tests) are displayed. The significance level is ,050.
a. Significance values have been adjusted by the Bonferroni correction for multiple tests.

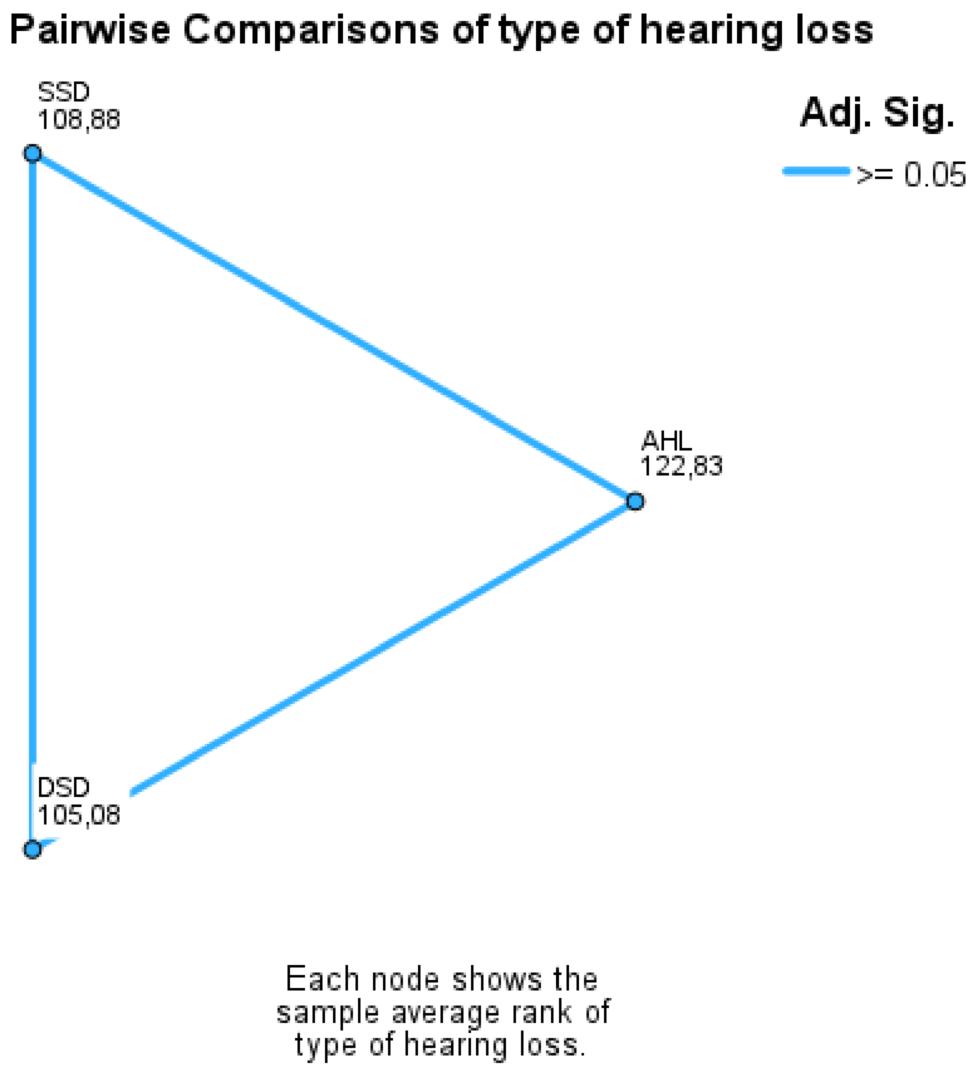

TQTotal across type of hearing loss

Independent-Samples Kruskal-Wallis Test Summary

Total N	220

Test Statistic	5,314[a]
Degree Of Freedom	2
Asymptotic Sig.(2-sided test)	,070

a. The test statistic is adjusted for ties.

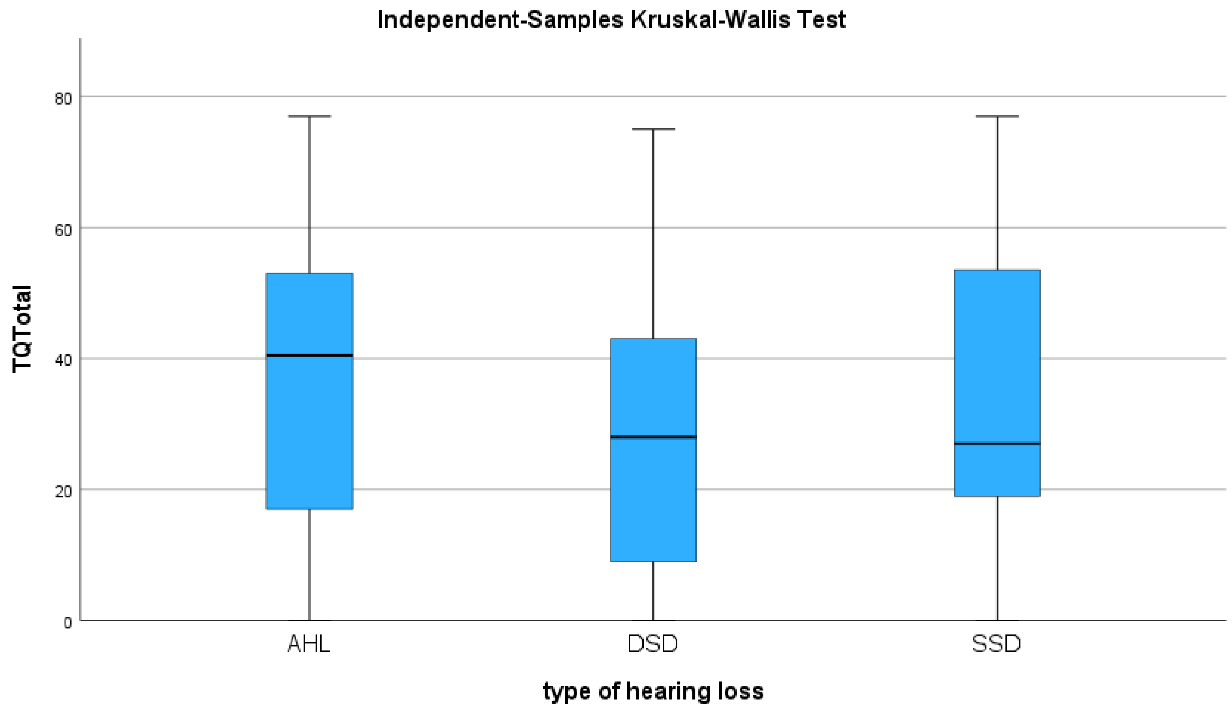

Pairwise Comparisons of type of hearing loss

Sample 1-Sample 2	Test Statistic	Std. Error	Std. Test Statistic	Sig.	Adj. Sig.[a]
DSD-SSD	-14,079	11,320	-1,244	,214	,641
DSD-AHL	22,632	10,188	2,221	,026	,079
SSD-AHL	8,553	12,803	,668	,504	1,000

Each row tests the null hypothesis that the Sample 1 and Sample 2 distributions are the same.
Asymptotic significances (2-sided tests) are displayed. The significance level is ,050.
a. Significance values have been adjusted by the Bonferroni correction for multiple tests.

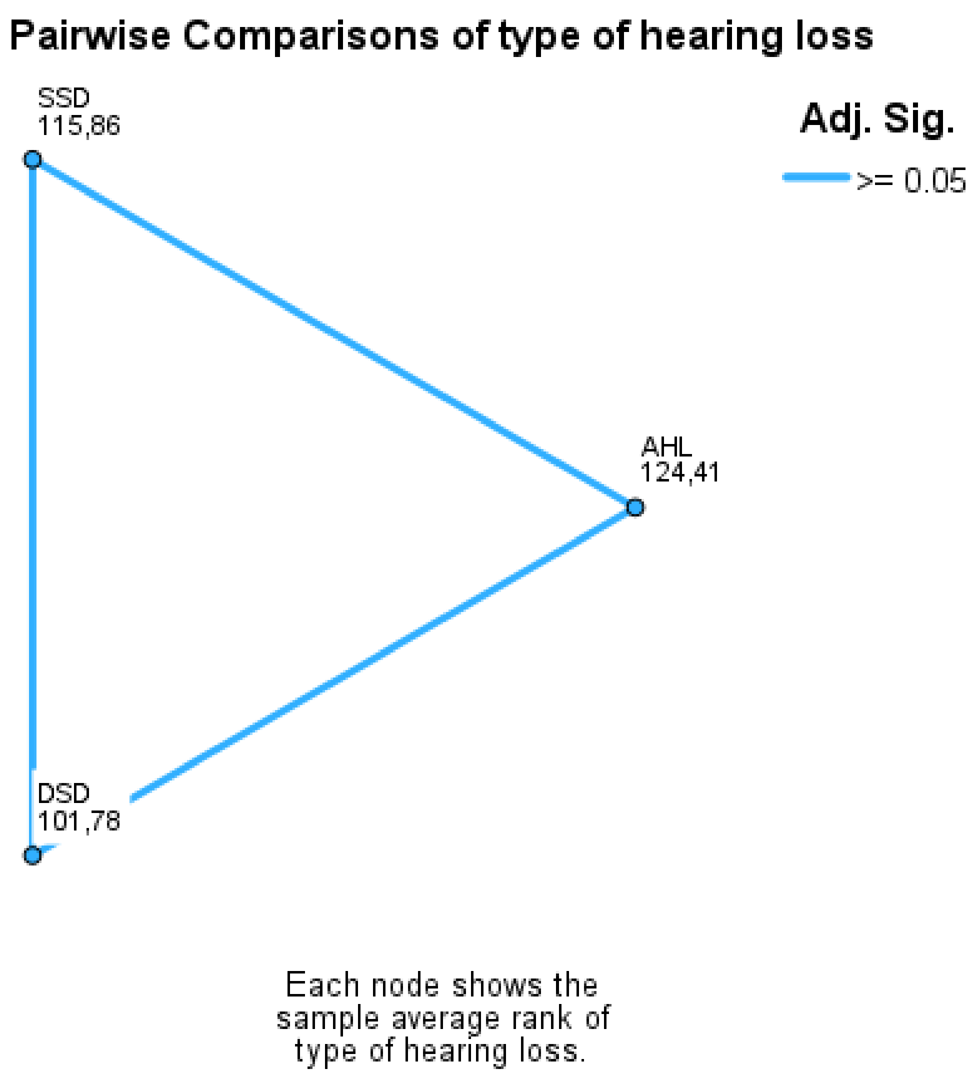

1_TQEC across type of hearing loss

Independent-Samples Kruskal-Wallis Test Summary

Total N	220

Test Statistic	,816[a]
Degree Of Freedom	2
Asymptotic Sig.(2-sided test)	,665

a. The test statistic is adjusted for ties.

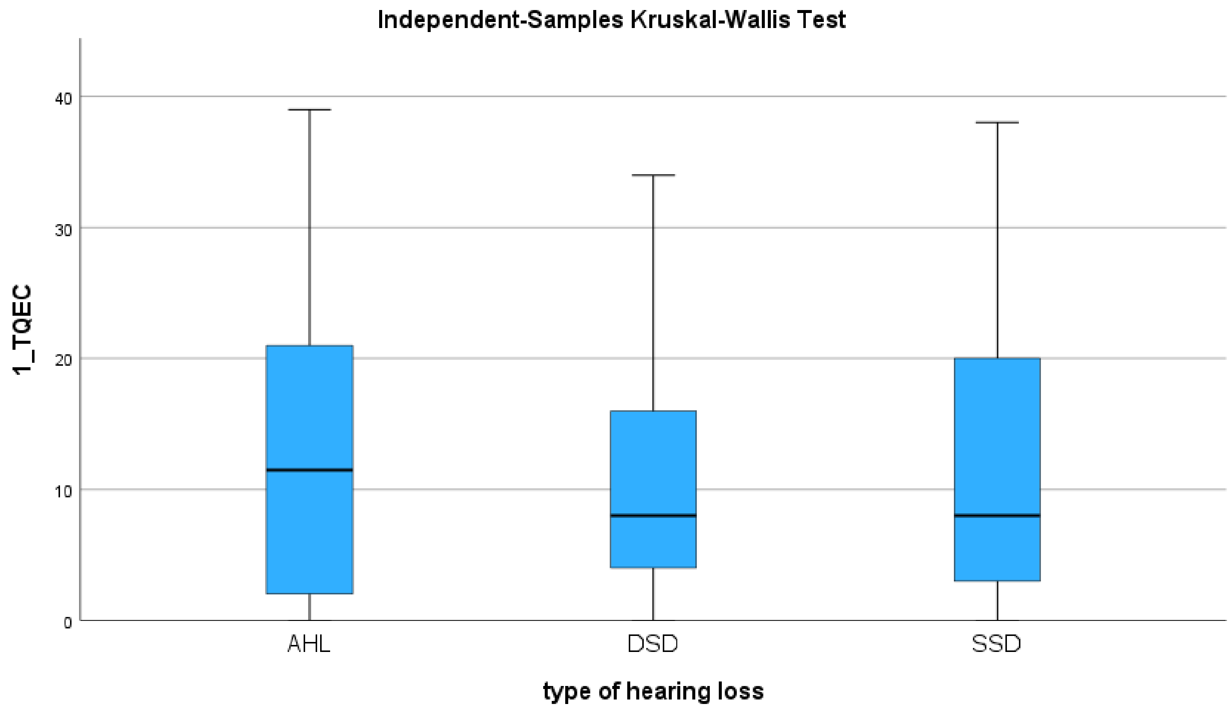

Pairwise Comparisons of type of hearing loss

Sample 1-Sample 2	Test Statistic	Std. Error	Std. Test Statistic	Sig.	Adj. Sig.[a]
DSD-SSD	-5,321	11,312	-,470	,638	1,000
DSD-AHL	8,917	10,181	,876	,381	1,000
SSD-AHL	3,596	12,794	,281	,779	1,000

Each row tests the null hypothesis that the Sample 1 and Sample 2 distributions are the same.

Asymptotic significances (2-sided tests) are displayed. The significance level is ,050.

a. Significance values have been adjusted by the Bonferroni correction for multiple tests.

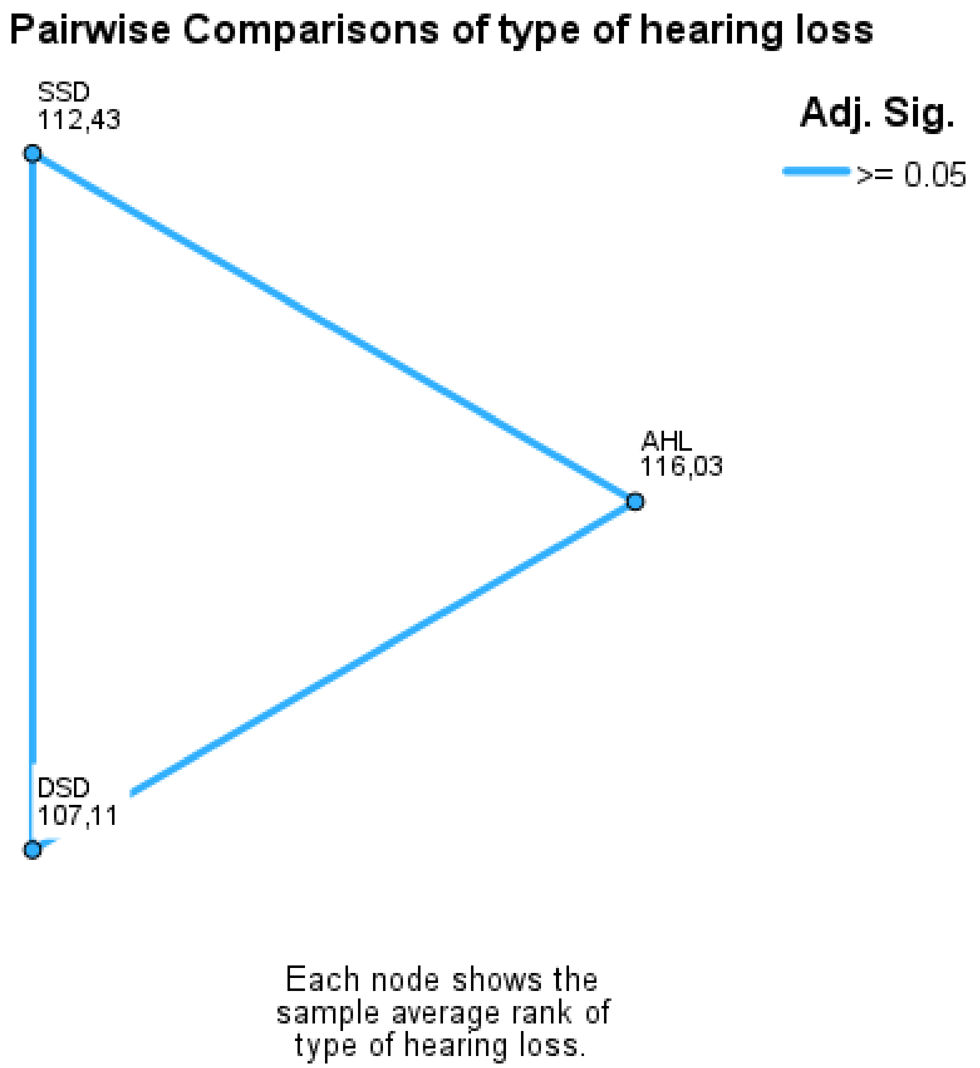

1_TQI across type of hearing loss

Independent-Samples Kruskal-Wallis Test Summary

Total N	220

Test Statistic	1,790[a]
Degree Of Freedom	2
Asymptotic Sig.(2-sided test)	,409

a. The test statistic is adjusted for ties.

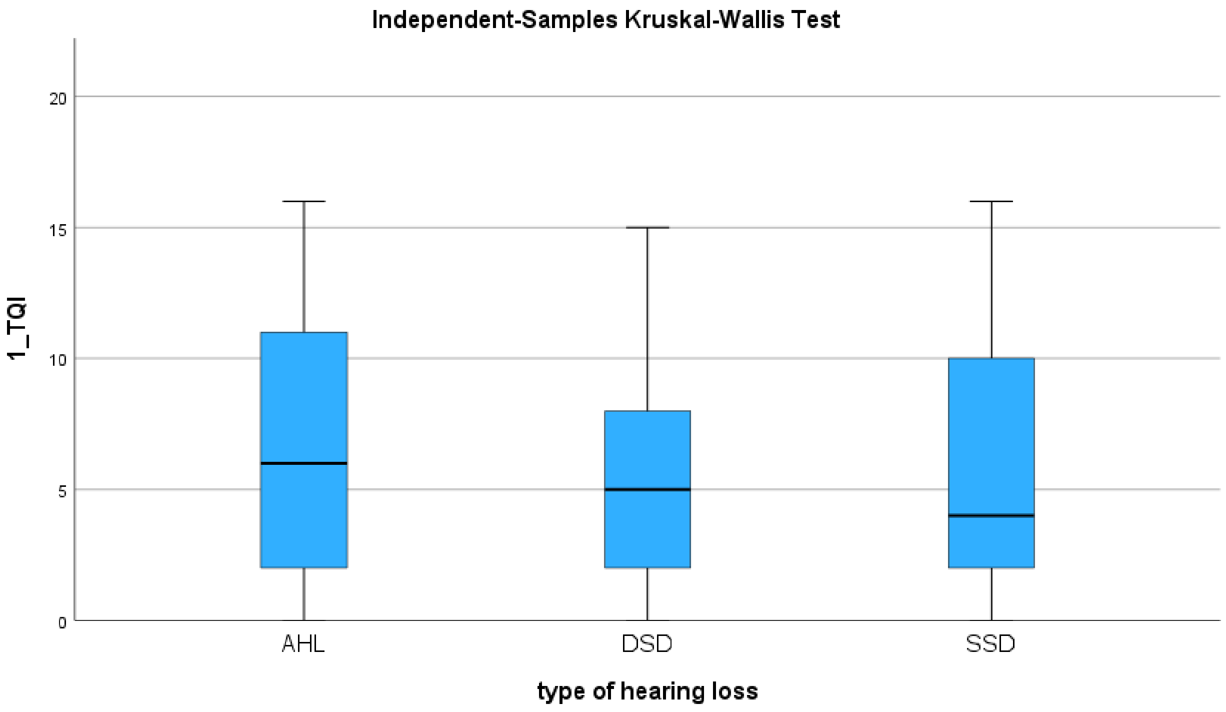

Pairwise Comparisons of type of hearing loss

Sample 1-Sample 2	Test Statistic	Std. Error	Std. Test Statistic	Sig.	Adj. Sig.[a]
DSD-SSD	-7,470	11,288	-,662	,508	1,000
DSD-AHL	13,268	10,159	1,306	,192	,575
SSD-AHL	5,799	12,767	,454	,650	1,000

Each row tests the null hypothesis that the Sample 1 and Sample 2 distributions are the same.
Asymptotic significances (2-sided tests) are displayed. The significance level is ,050.
a. Significance values have been adjusted by the Bonferroni correction for multiple tests.

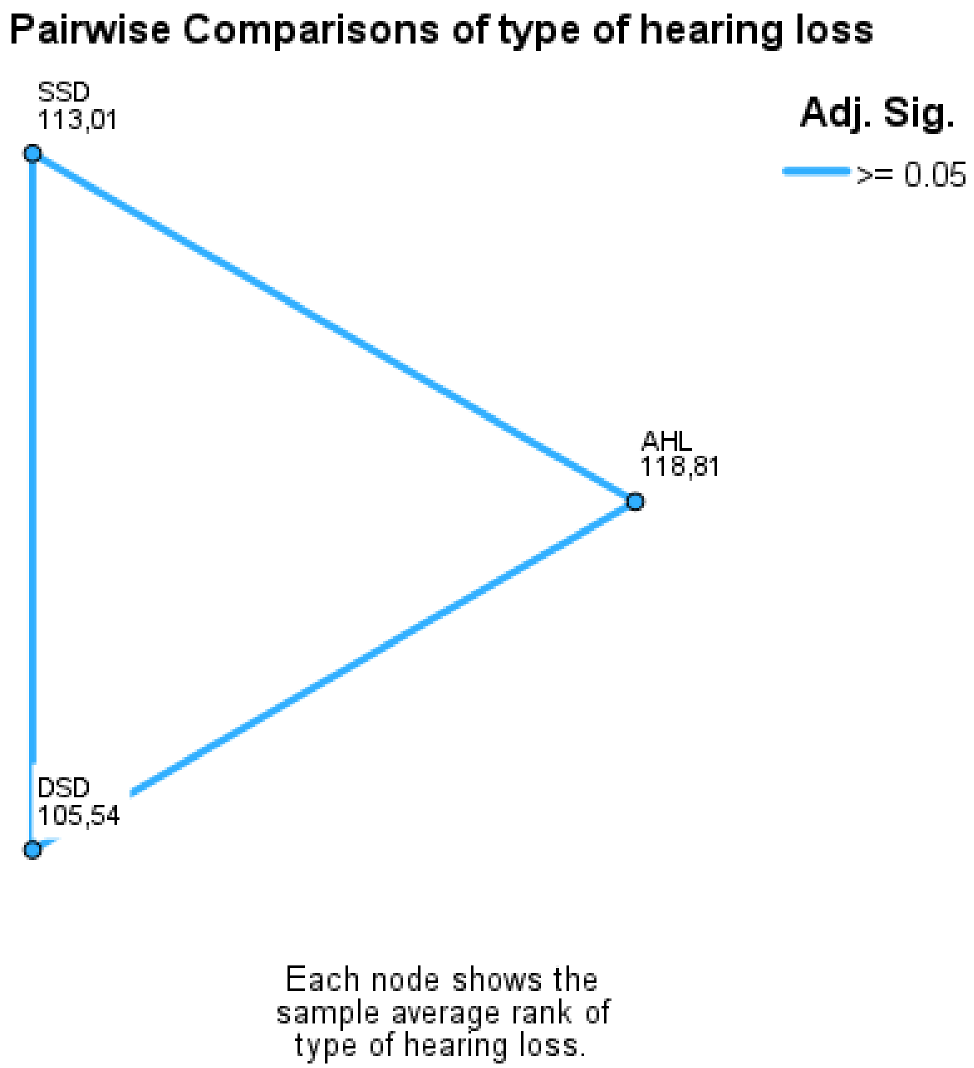

1_TQA across type of hearing loss

Independent-Samples Kruskal-Wallis Test Summary

Total N	220

Test Statistic	1,473[a]
Degree Of Freedom	2
Asymptotic Sig.(2-sided test)	,479

a. The test statistic is adjusted for ties.

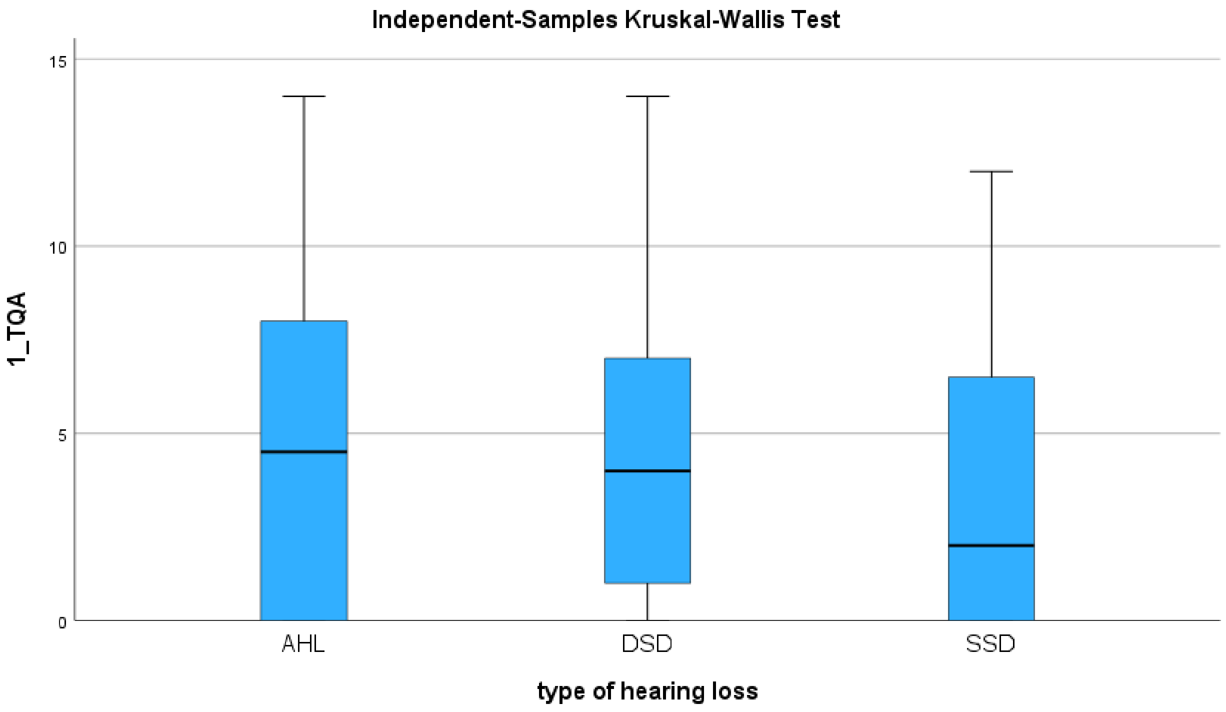

Pairwise Comparisons of type of hearing loss

Sample 1-Sample 2	Test Statistic	Std. Error	Std. Test Statistic	Sig.	Adj. Sig.[a]
SSD-DSD	12,195	11,209	1,088	,277	,830
SSD-AHL	14,145	12,677	1,116	,265	,794
DSD-AHL	1,949	10,088	,193	,847	1,000

Each row tests the null hypothesis that the Sample 1 and Sample 2 distributions are the same.
Asymptotic significances (2-sided tests) are displayed. The significance level is ,050.
a. Significance values have been adjusted by the Bonferroni correction for multiple tests.

Pairwise Comparisons of type of hearing loss

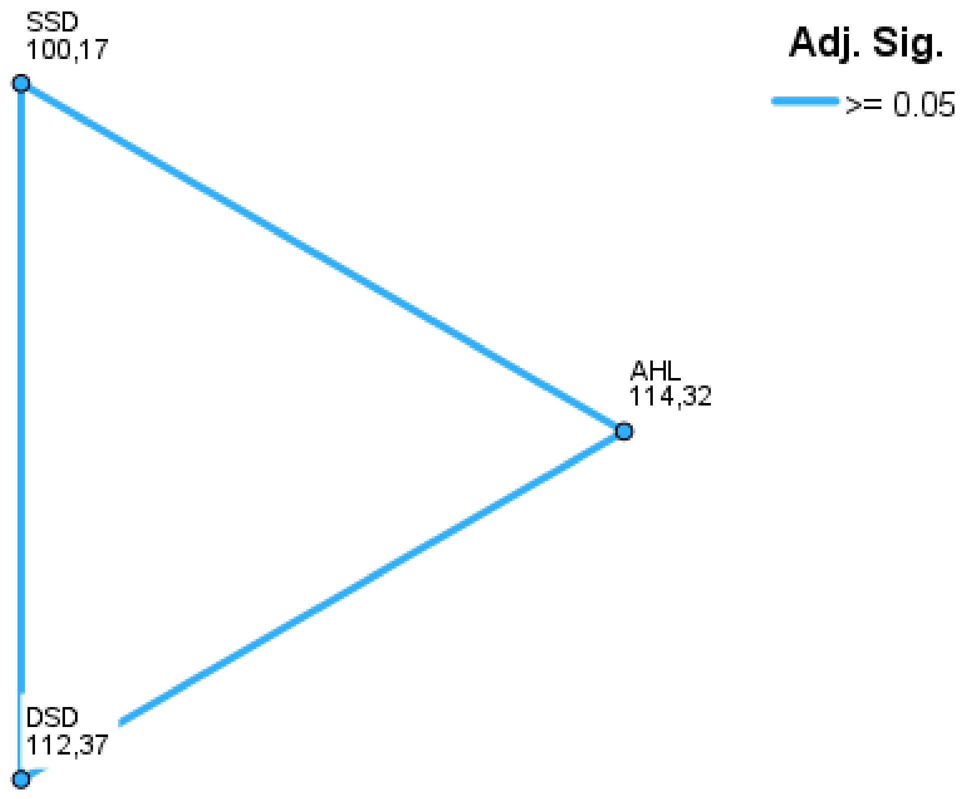

Each node shows the sample average rank of type of hearing loss.

1_TQSI across type of hearing loss

Independent-Samples Kruskal-Wallis Test Summary

Total N	220

Test Statistic	,104[a]
Degree Of Freedom	2
Asymptotic Sig.(2-sided test)	,949

a. The test statistic is adjusted for ties.

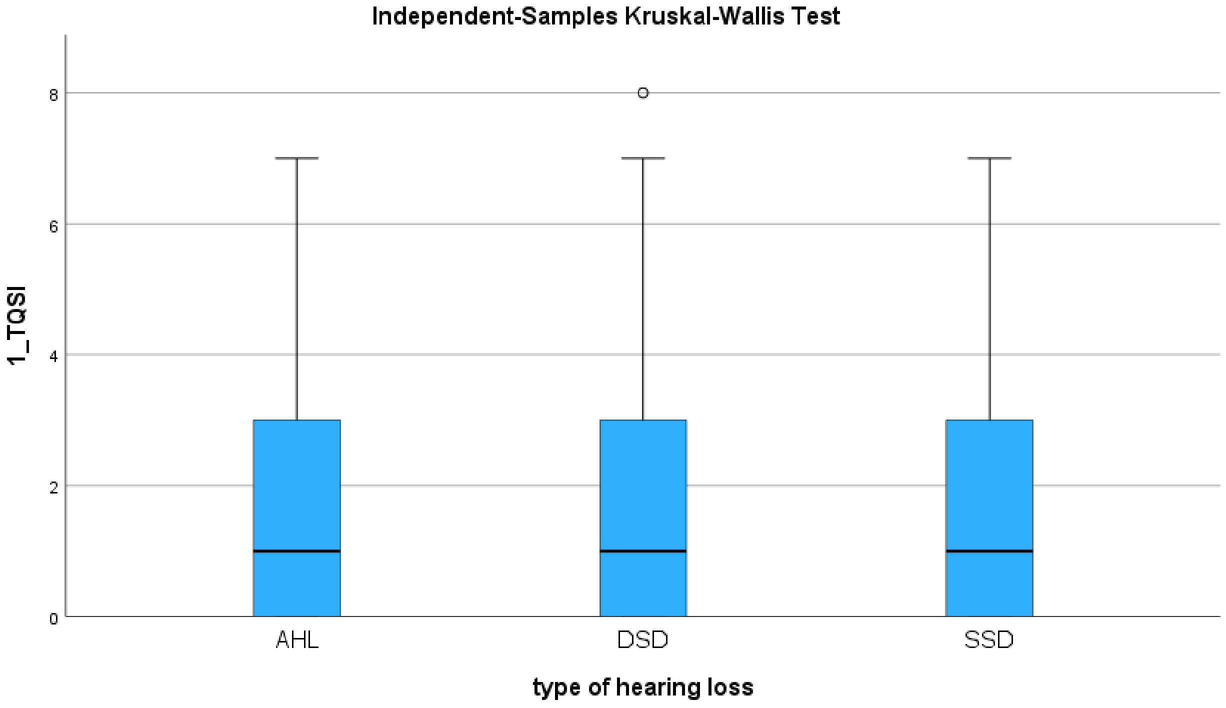

Pairwise Comparisons of type of hearing loss

Sample 1-Sample 2	Test Statistic	Std. Error	Std. Test Statistic	Sig.	Adj. Sig.[a]
SSD-DSD	2,332	10,809	,216	,829	1,000
SSD-AHL	3,949	12,225	,323	,747	1,000
DSD-AHL	1,617	9,728	,166	,868	1,000

Each row tests the null hypothesis that the Sample 1 and Sample 2 distributions are the same.

Asymptotic significances (2-sided tests) are displayed. The significance level is ,050.

a. Significance values have been adjusted by the Bonferroni correction for multiple tests.

Pairwise Comparisons of type of hearing loss

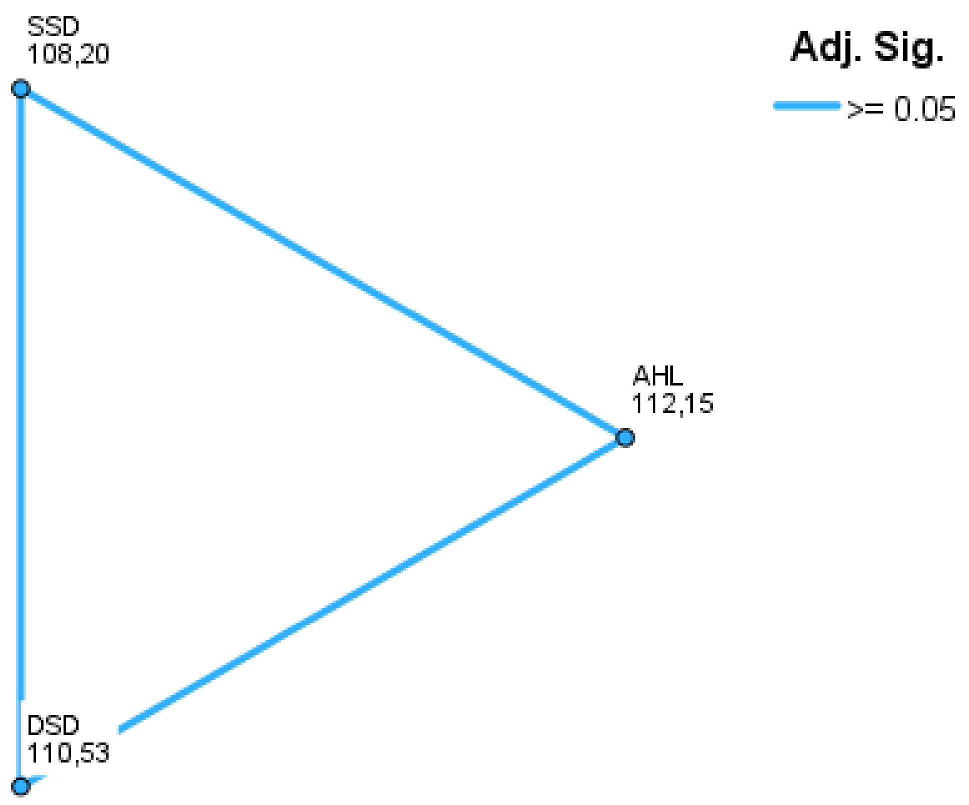

Each node shows the sample average rank of type of hearing loss.

1_TQSO across type of hearing loss

Independent-Samples Kruskal-Wallis Test Summary

Total N	220

Test Statistic	2,992[a]
Degree Of Freedom	2
Asymptotic Sig.(2-sided test)	,224

a. The test statistic is adjusted for ties.

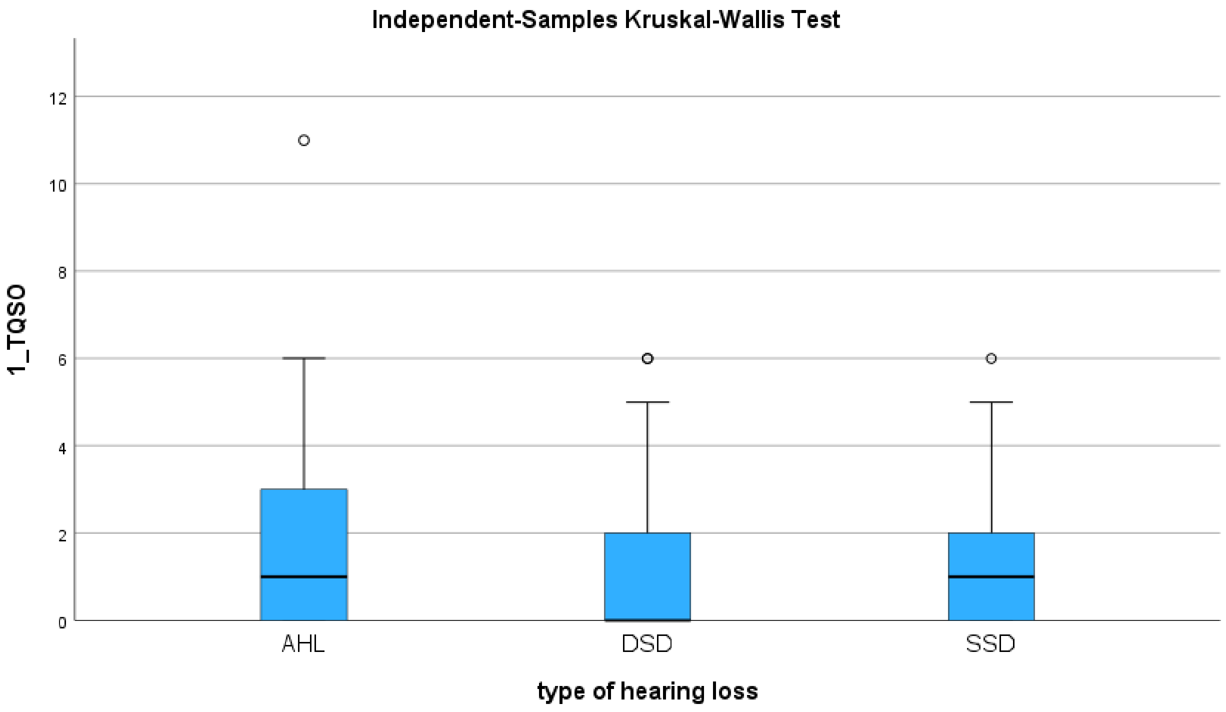

Pairwise Comparisons of type of hearing loss

Sample 1-Sample 2	Test Statistic	Std. Error	Std. Test Statistic	Sig.	Adj. Sig.[a]
DSD-SSD	-12,194	10,519	-1,159	,246	,739
DSD-AHL	14,850	9,467	1,569	,117	,350
SSD-AHL	2,656	11,897	,223	,823	1,000

Each row tests the null hypothesis that the Sample 1 and Sample 2 distributions are the same.

Asymptotic significances (2-sided tests) are displayed. The significance level is ,050.

a. Significance values have been adjusted by the Bonferroni correction for multiple tests.

Pairwise Comparisons of type of hearing loss

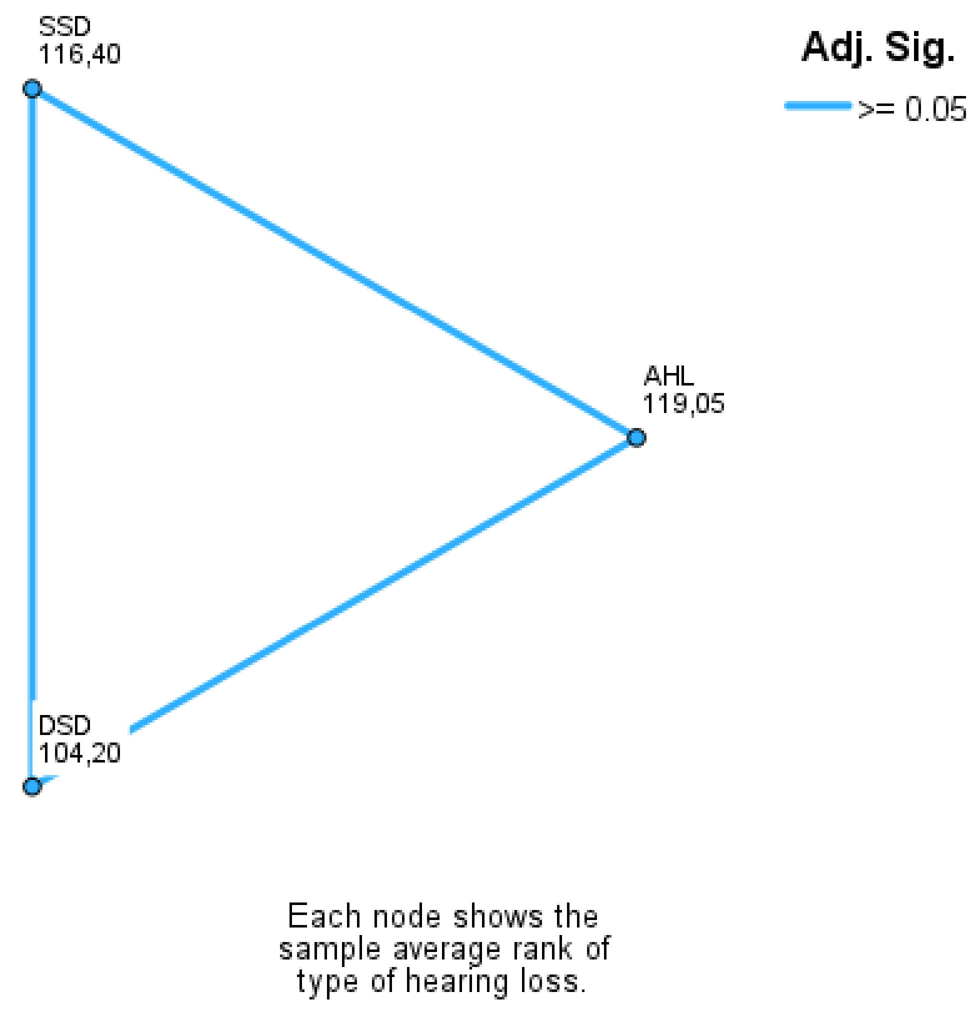

1_TQTotal across type of hearing loss

Independent-Samples Kruskal-Wallis Test Summary

Total N	220

Test Statistic	,715[a]
Degree Of Freedom	2
Asymptotic Sig.(2-sided test)	,699

a. The test statistic is adjusted for ties.

Independent-Samples Kruskal-Wallis Test

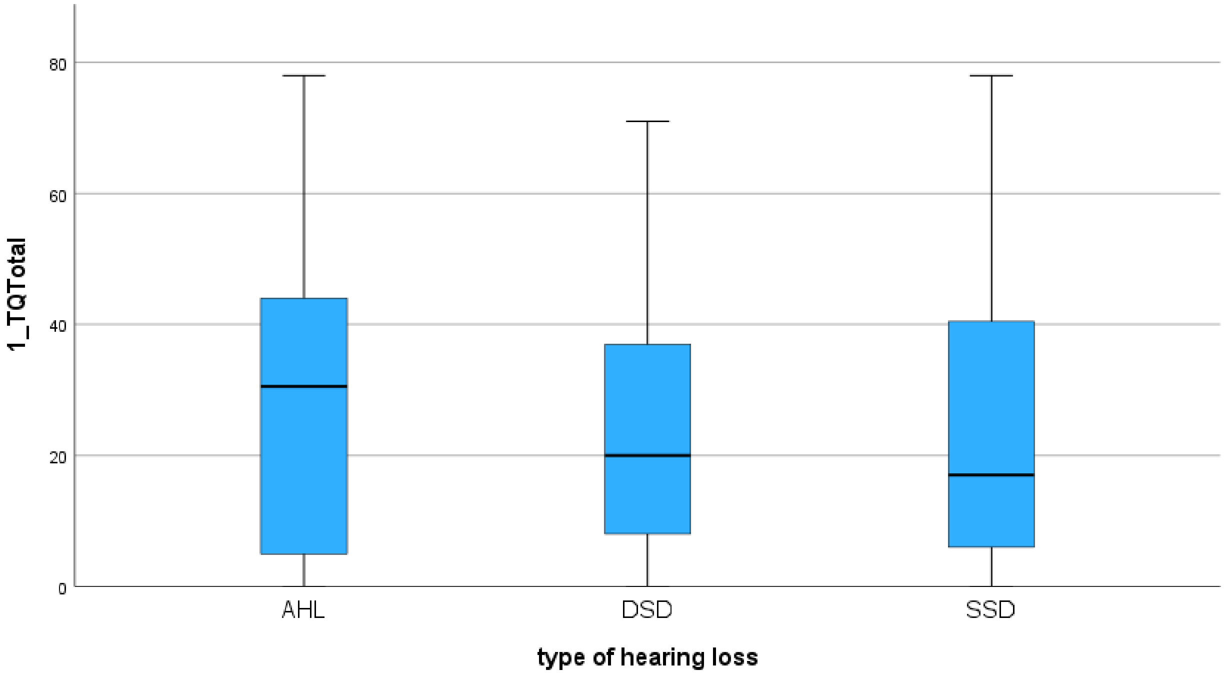

Pairwise Comparisons of type of hearing loss

Sample 1-Sample 2	Test Statistic	Std. Error	Std. Test Statistic	Sig.	Adj. Sig.[a]
DSD-SSD	-1,779	11,321	-,157	,875	1,000
DSD-AHL	8,562	10,189	,840	,401	1,000
SSD-AHL	6,782	12,804	,530	,596	1,000

Each row tests the null hypothesis that the Sample 1 and Sample 2 distributions are the same.

Asymptotic significances (2-sided tests) are displayed. The significance level is ,050.

a. Significance values have been adjusted by the Bonferroni correction for multiple tests.

Pairwise Comparisons of type of hearing loss

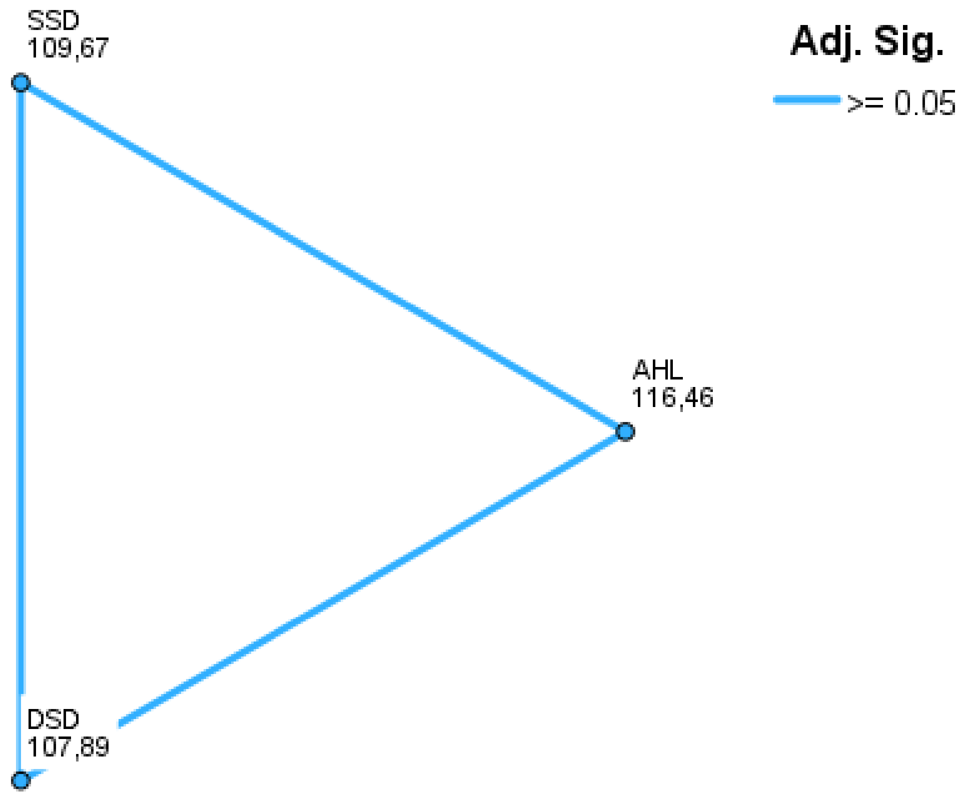

Each node shows the sample average rank of type of hearing loss.

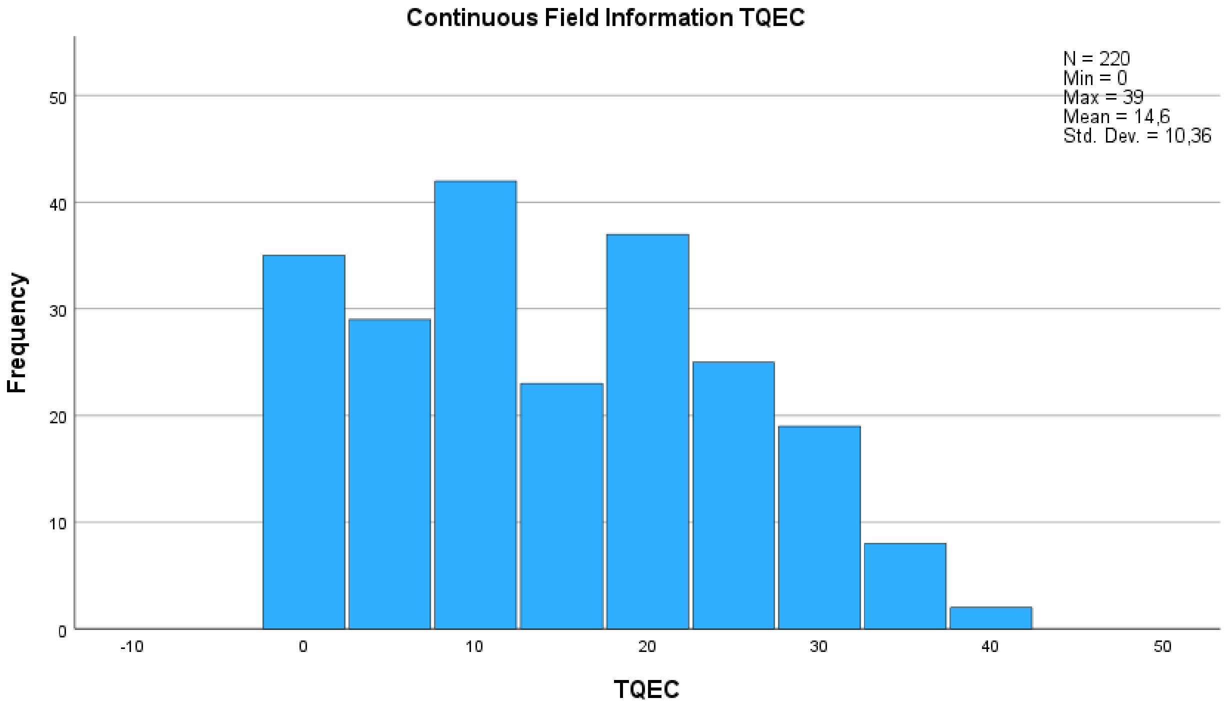
Continuous Field Information TQEC
N = 220
Min = 0
Max = 39
Mean = 14,6
Std. Dev. = 10,36
Frequency
TQEC
0
10
20
30
40
50
-10

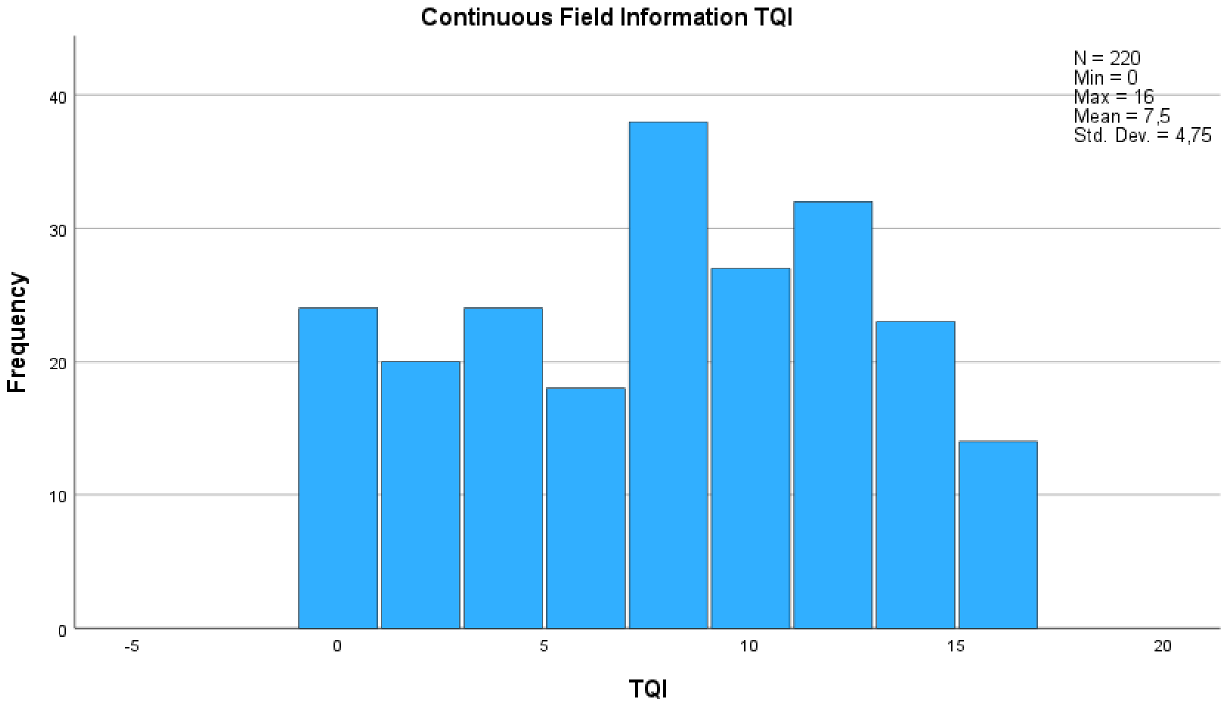
Continuous Field Information TQI
N = 220
Min = 0
Max = 16
Mean = 7,5
Std. Dev. = 4,75
Frequency
TQI
0
10
20
30
40
-5
5
15

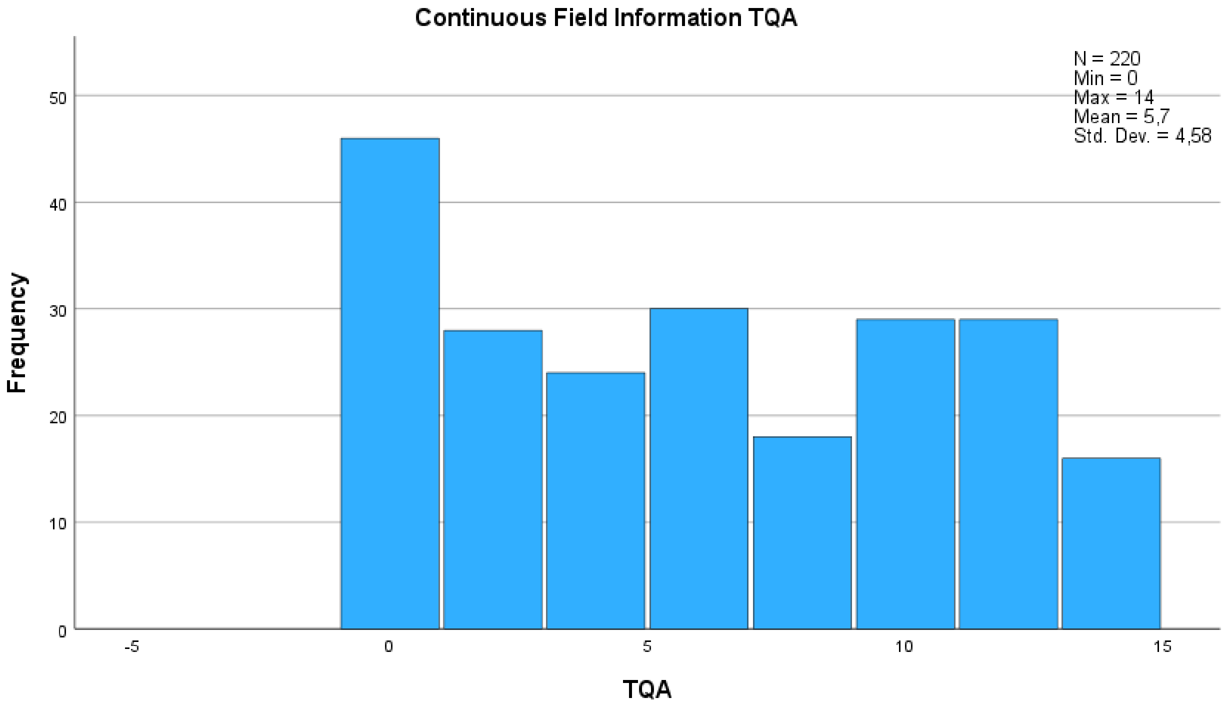
Continuous Field Information TQA
N = 220
Min = 0
Max = 14
Mean = 5,7
Std. Dev. = 4,58
Frequency
TQA

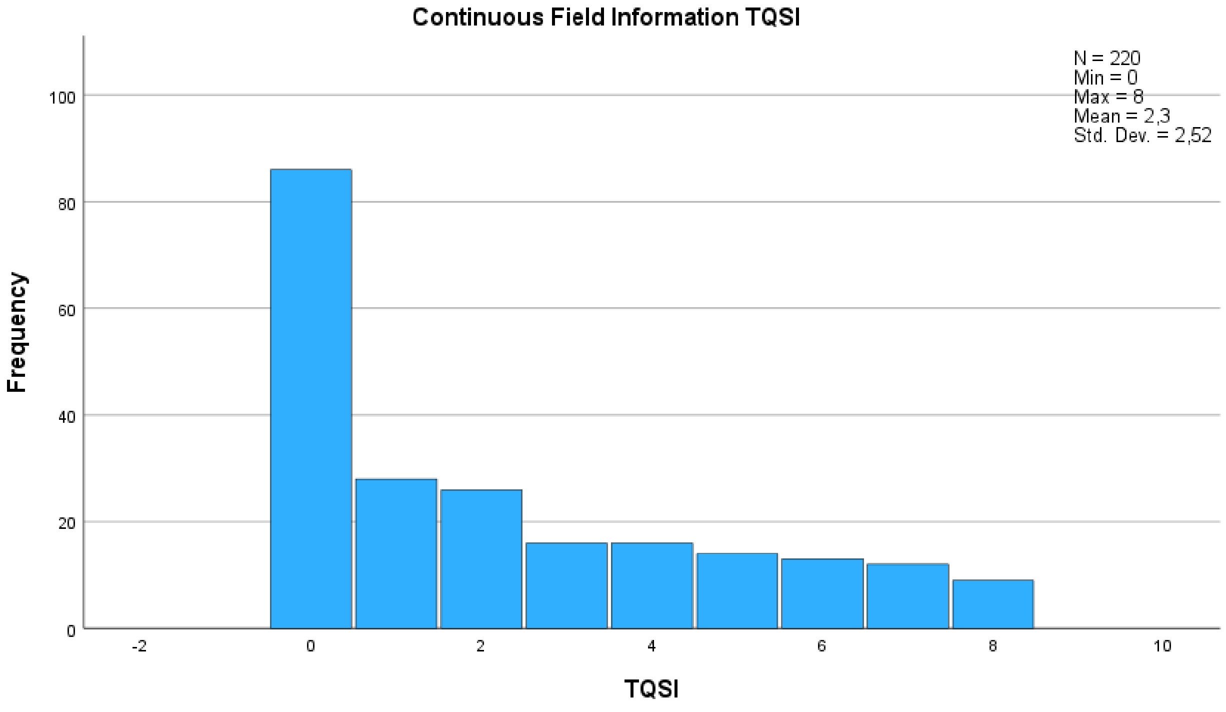
Continuous Field Information TQSI
N = 220
Min = 0
Max = 8
Mean = 2,3
Std. Dev. = 2,52
Frequency
TQSI

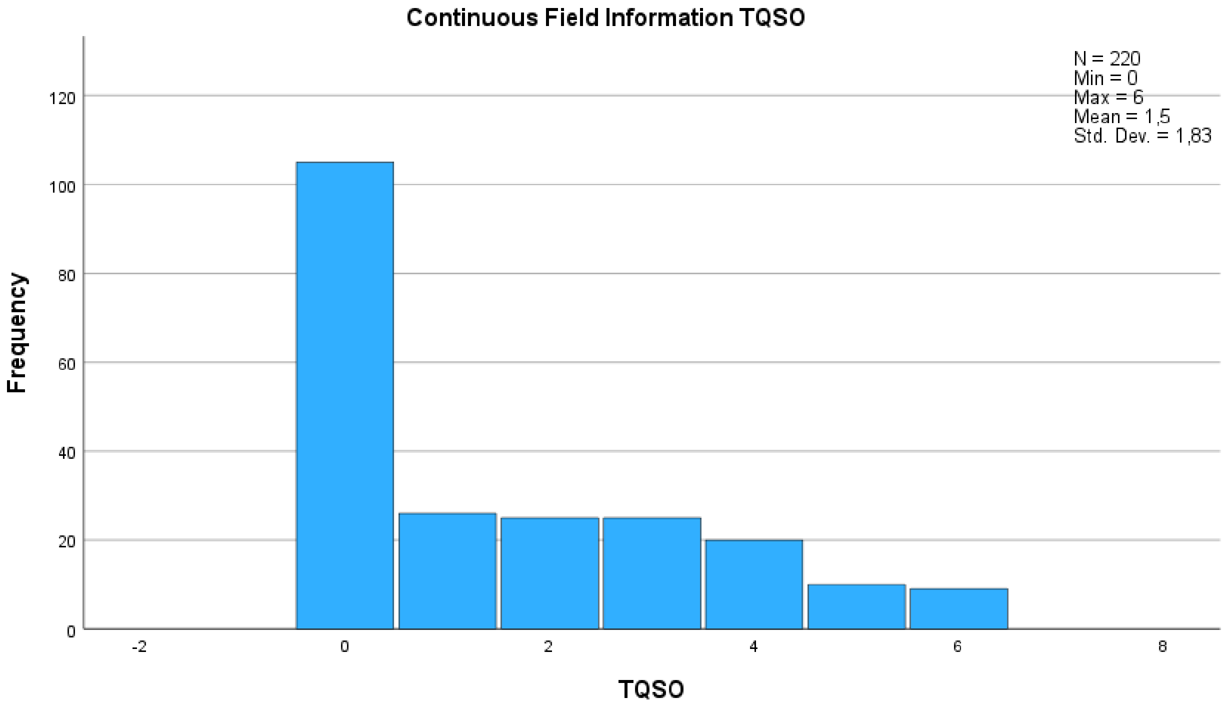
Continuous Field Information TQSO
N = 220
Min = 0
Max = 6
Mean = 1,5
Std. Dev. = 1,83
Frequency
120
100
80
60
40
20
0
-2
0
2
4
6
8
TQSO

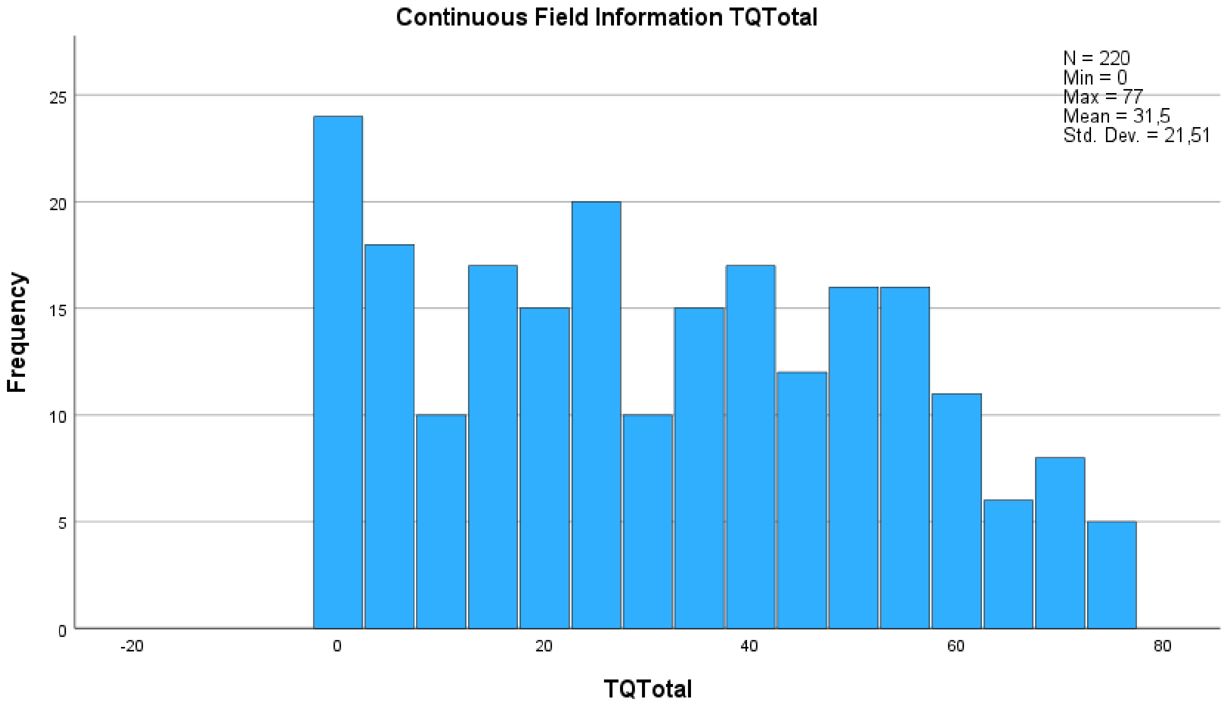
Continuous Field Information TQTotal
N = 220
Min = 0
Max = 77
Mean = 31,5
Std. Dev. = 21,51
Frequency
25
20
15
10
5
0
-20
0
20
40
60
80
TQTotal

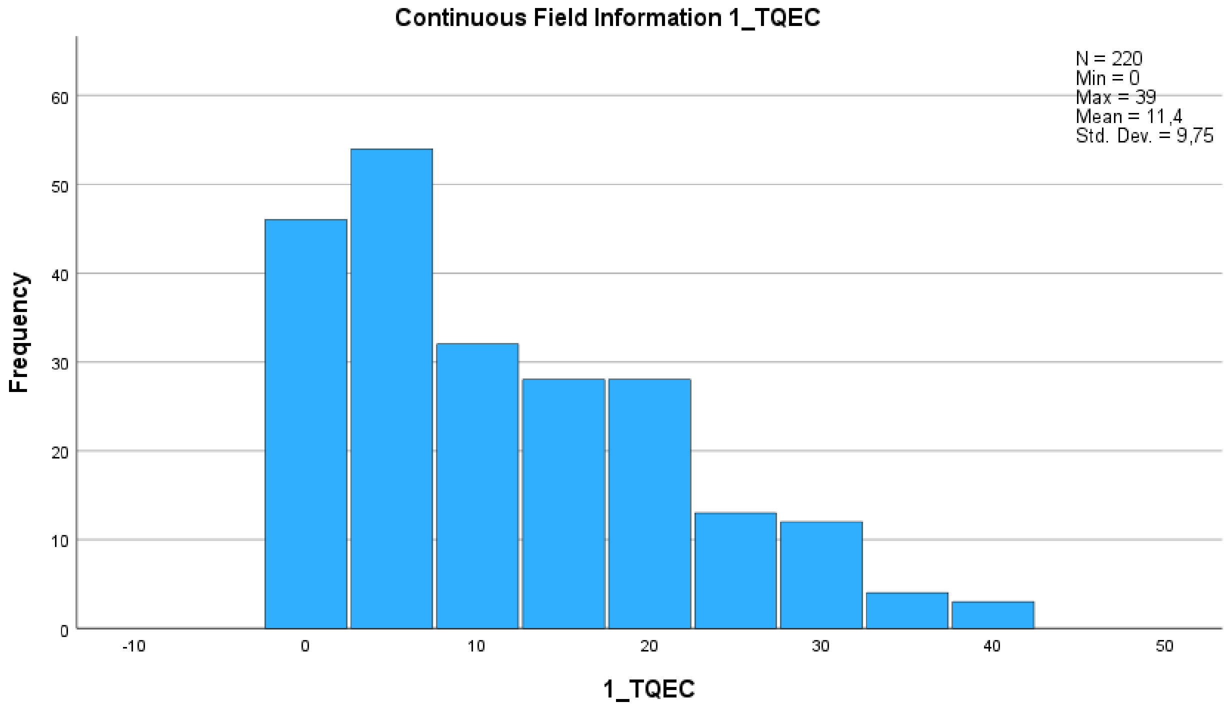
Continuous Field Information 1_TQEC
N = 220
Min = 0
Max = 39
Mean = 11,4
Std. Dev. = 9,75
Frequency
1_TQEC

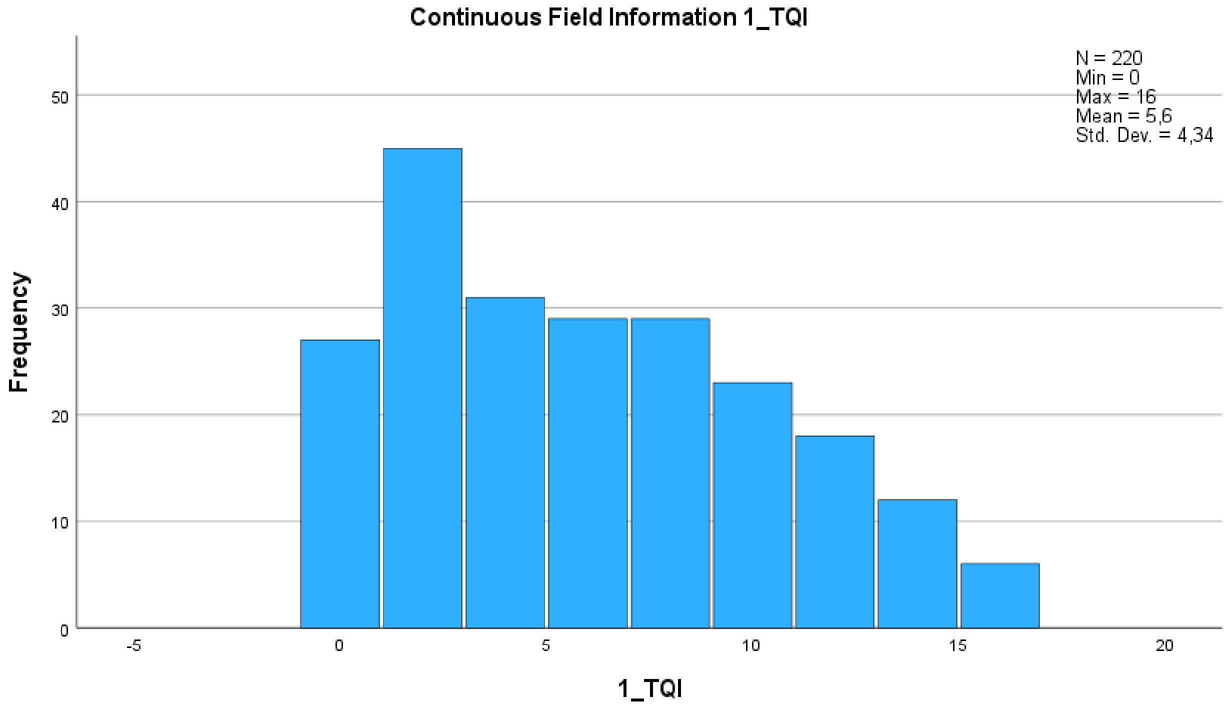
Continuous Field Information 1_TQI
N = 220
Min = 0
Max = 16
Mean = 5,6
Std. Dev. = 4,34
Frequency
1_TQI

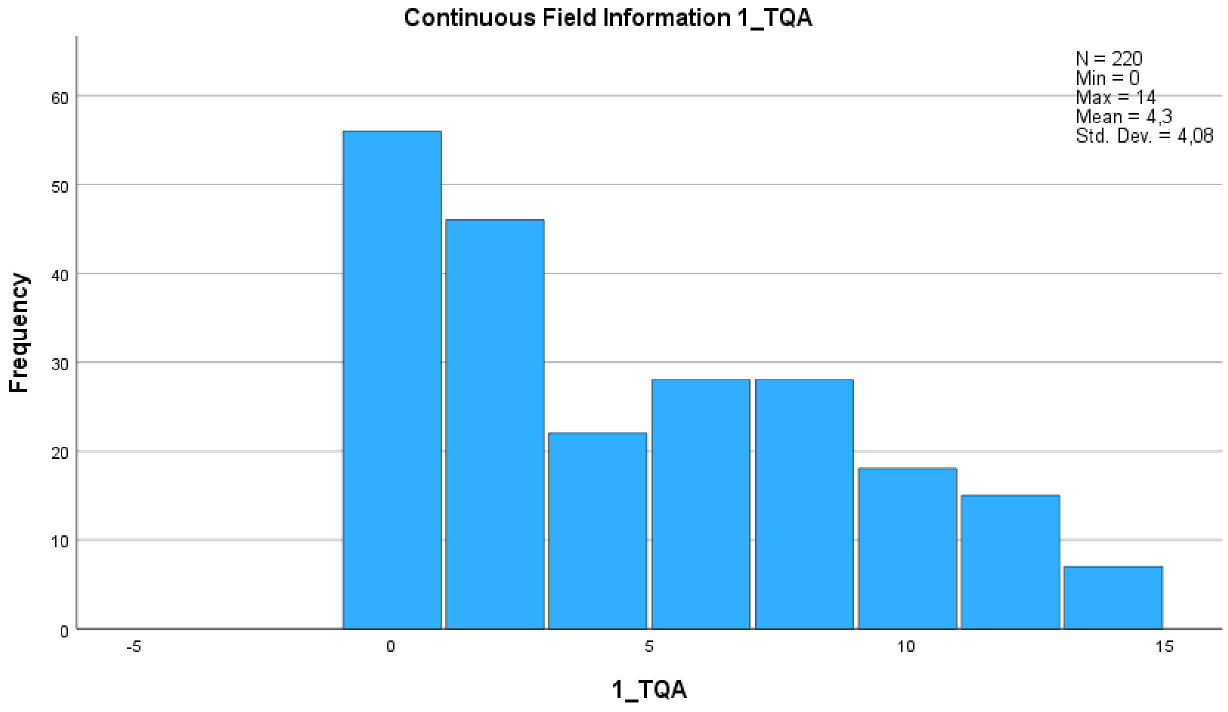
Continuous Field Information 1_TQA
N = 220
Min = 0
Max = 14
Mean = 4,3
Std. Dev. = 4,08
Frequency
0
10
20
30
40
50
60
-5
0
5
10
15
1_TQA

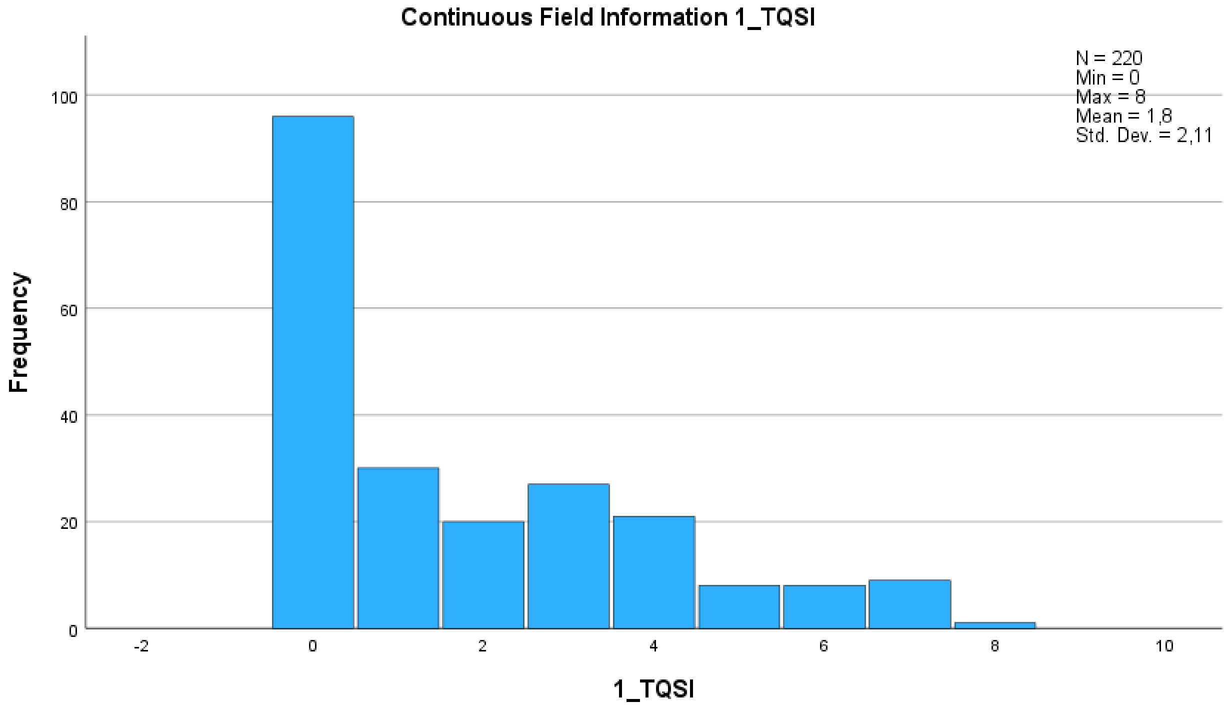
Continuous Field Information 1_TQSI
N = 220
Min = 0
Max = 8
Mean = 1,8
Std. Dev. = 2,11
Frequency
0
20
40
60
80
100
-2
0
2
4
6
8
10
1_TQSI

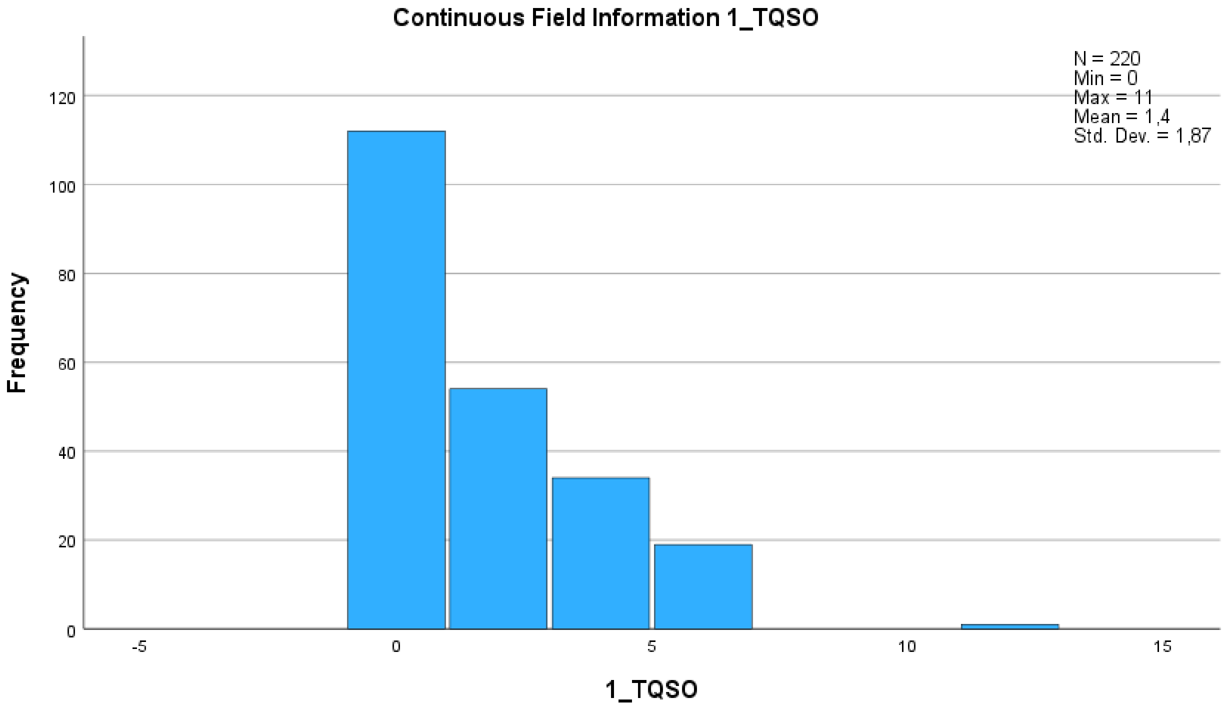

Continuous Field Information 1_TQSO
N = 220
Min = 0
Max = 11
Mean = 1,4
Std. Dev. = 1,87
Frequency
120
100
80
60
40
20
0
-5
0
5
10
15
1_TQSO

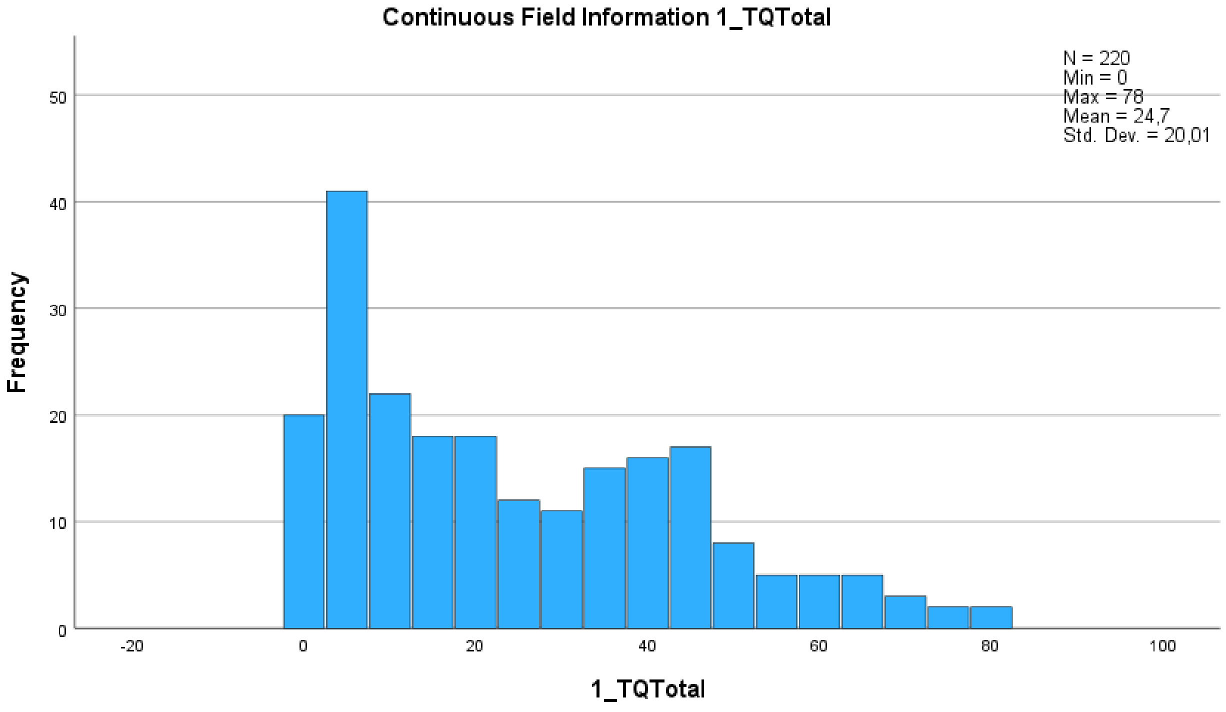

Continuous Field Information 1_TQTotal
N = 220
Min = 0
Max = 78
Mean = 24,7
Std. Dev. = 20,01
Frequency
50
40
30
20
10
0
-20
0
20
40
60
80
100
1_TQTotal

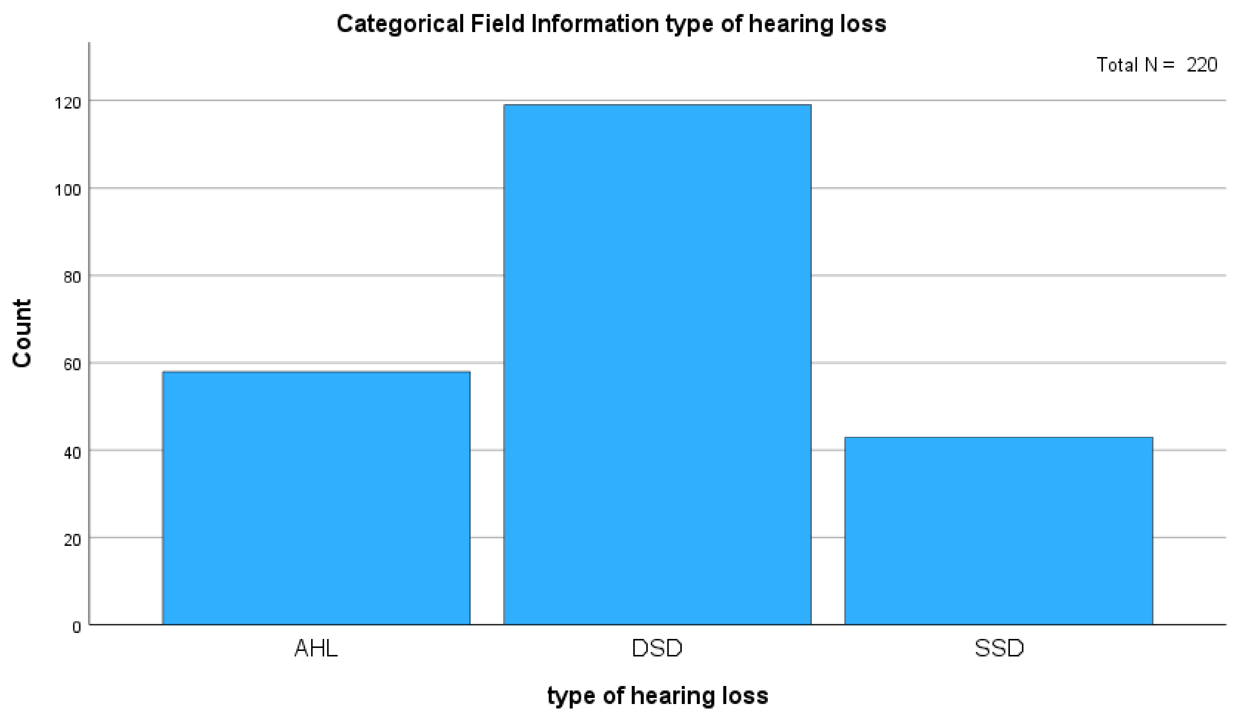
Categorical Field Information type of hearing loss
Total N = 220
Count
120
100
80
60
40
20
0
AHL
DSD
SSD
type of hearing loss
