# Supplementary material for: Changes in Speech Intelligibility, Health-Related Quality of Life, Depressive Symptoms, Anxiety, Perceived Stress, and Tinnitus-Induced Distress, in a Cohort of 227 Adults One Year After Cochlear Implantation: A Decade of Experience from a Single Tertiary Center
Source: J Clin Med. 2025 Nov 17;14(22):8143. doi: 10.3390/jcm14228143 (PMC12653197; doi:10.3390/jcm14228143)
Supplement: Supplementary file 1 [file jcm-14-08143-s001.zip › S4.pdf]

S4: Nonparametric Correlations in groups AHL, DSD, SSD before and after implantation

BEFORE IMPLANTATION

Correlations

type of hearing loss				NCIQtotal	OITotal	ADSL	GAD	PSQ
AHL	Spearman's rho	OITotal	Correlation Coefficient	,700**				
			Sig. (2-tailed)	<,001				
			N	58				
		ADSL	Correlation Coefficient	-,425**	-,354**			
			Sig. (2-tailed)	,001	,007			
			N	56	56			
		GAD	Correlation Coefficient	-,402**	-,254	,811**		
			Sig. (2-tailed)	,002	,058	<,001		
			N	56	56	54		
		PSQ	Correlation Coefficient	-,405**	-,256	,768**	,825**	
			Sig. (2-tailed)	,002	,053	<,001	<,001	
			N	58	58	56	56	
		TQTotal	Correlation Coefficient	-,521**	-,408**	,653**	,691**	,631**
			Sig. (2-tailed)	<,001	,001	<,001	<,001	<,001
			N	58	58	56	56	58
DSD	Spearman's rho	OITotal	Correlation Coefficient	,670**				
			Sig. (2-tailed)	<,001				
			N	119				
		ADSL	Correlation Coefficient	-,331**	-,227*			
			Sig. (2-tailed)	<,001	,013			
			N	119	119			
		GAD	Correlation Coefficient	-,279**	-,153	,702**		
			Sig. (2-tailed)	,002	,097	<,001		
			N	119	119	119		
		PSQ	Correlation Coefficient	-,285**	-,193*	,744**	,673**	
			Sig. (2-tailed)	,002	,035	<,001	<,001	
			N	119	119	119	119	
		TQTotal	Correlation Coefficient	-,202*	-,177	,430**	,303**	,354**
			Sig. (2-tailed)	,028	,054	<,001	<,001	<,001
			N	119	119	119	119	119
SSD	Spearman's rho	OITotal	Correlation Coefficient	,695**				
			Sig. (2-tailed)	<,001				

		N	43				
	ADSL	Correlation Coefficient	-,182	-,184			
		Sig. (2-tailed)	,242	,238			
		N	43	43			
	GAD	Correlation Coefficient	-,351*	-,320*	,607**		
		Sig. (2-tailed)	,021	,037	<,001		
		N	43	43	43		
	PSQ	Correlation Coefficient	-,391**	-,389**	,676**	,739**	
		Sig. (2-tailed)	,009	,010	<,001	<,001	
		N	43	43	43	43	
	TQTotal	Correlation Coefficient	-,536**	-,360*	,293	,470**	,417**
		Sig. (2-tailed)	<,001	,018	,056	,001	,005
		N	43	43	43	43	43

**. Correlation is significant at the 0.01 level (2-tailed).

*. Correlation is significant at the 0.05 level (2-tailed).

AFTER IMPLANTATION

Nonparametric Correlations

Correlations

type of hearing loss				1_NCIQtotal	1_OITotal	1_ADSL	1_GAD	1_PSQ
AHL	Spearman's rho	1_OITotal	Correlation Coefficient	,785**				
			Sig. (2-tailed)	<,001				
			N	58				
		1_ADSL	Correlation Coefficient	-,486**	-,617**			
			Sig. (2-tailed)	<,001	<,001			
			N	58	58			
		1_GAD	Correlation Coefficient	-,428**	-,401**	,718**		
			Sig. (2-tailed)	<,001	,002	<,001		
			N	58	58	58		
		1_PSQ	Correlation Coefficient	-,454**	-,545**	,818**	,835**	
			Sig. (2-tailed)	<,001	<,001	<,001	<,001	
			N	58	58	58	58	
		1_TQTotal	Correlation Coefficient	-,492**	-,455**	,550**	,718**	,663**
			Sig. (2-tailed)	<,001	<,001	<,001	<,001	<,001
			N	58	58	58	58	58
DSD	Spearman's rho	1_OITotal	Correlation Coefficient	,712**				
			Sig. (2-tailed)	<,001				
			N	119				
		1_ADSL	Correlation Coefficient	-,527**	-,354**			
			Sig. (2-tailed)	<,001	<,001			
			N	115	115			
		1_GAD	Correlation Coefficient	-,345**	-,258**	,713**		
			Sig. (2-tailed)	<,001	,005	<,001		
			N	119	119	115		
		1_PSQ	Correlation Coefficient	-,450**	-,231*	,726**	,636**	
			Sig. (2-tailed)	<,001	,011	<,001	<,001	
			N	119	119	115	119	
		1_TQTotal	Correlation Coefficient	-,318**	-,264**	,317**	,190*	,191*
			Sig. (2-tailed)	<,001	,004	<,001	,038	,037
			N	119	119	115	119	119

SSD	Spearman's rho	1_OITotal	Correlation Coefficient	,540**				
			Sig. (2-tailed)	<,001				
			N	42				
		1_ADSL	Correlation Coefficient	-,581**	-,362*			
			Sig. (2-tailed)	<,001	,018			
			N	43	42			
		1_GAD	Correlation Coefficient	-,482**	-,064	,665**		
			Sig. (2-tailed)	,001	,692	<,001		
			N	42	41	42		
		1_PSQ	Correlation Coefficient	-,535**	-,217	,789**	,782**	
			Sig. (2-tailed)	<,001	,173	<,001	<,001	
			N	42	41	42	42	
		1_TQTotal	Correlation Coefficient	-,664**	-,442**	,470**	,480**	,461**
			Sig. (2-tailed)	<,001	,003	,001	,001	,002
			N	43	42	43	42	42

**. Correlation is significant at the 0.01 level (2-tailed).

*. Correlation is significant at the 0.05 level (2-tailed).
